# Synthesis and Evaluation of 2‑Substituted Quinazolin-4(3*H*)‑ones as Potential Antileukemic Agents

**DOI:** 10.1021/acsomega.5c04106

**Published:** 2025-08-01

**Authors:** Giorgio Antoniolli, Keli Lima, Gilberto Carlos Franchi, Carmen Silvia Passos Lima, João Agostinho Machado-Neto, Fernando Coelho

**Affiliations:** † Institute of Chemistry, 28132 University of Campinas, Campinas, SP 13083-862, Brazil; ‡ Faculty of Medicine, University of São Paulo, São Paulo, SP 01246-903, Brazil; § School of Medical Sciences, University of Campinas, Campinas, SP 13083-888, Brazil; ∥ Institute of Biomedical Sciences, University of São Paulo, São Paulo, SP 05508-000, Brazil

## Abstract

The increasing life expectancy and rising prevalence of cancer emphasize the need for innovative therapeutic strategies. Targeted therapies have revolutionized cancer treatment by offering greater specificity and reduced toxicity compared to traditional cytotoxic drugs. Acute leukemias, including acute lymphoblastic leukemia (ALL) and acute myeloid leukemia (AML), remain aggressive malignancies with poor outcomes, particularly in elderly patients. Despite advancements, resistance to chemotherapy and adverse effects necessitate the discovery of novel antitumor compounds. Quinazolines, a versatile class of heterocyclic compounds, exhibit diverse biological activities, including anticancer properties. In this study, 20 derivatives of 2-substituted quinazolin-4­(3*H*)-ones were synthesized via condensation of 2-aminobenzamide with aldehydes in dimethyl sulfoxide. The compounds were characterized using IR, ^1^H NMR, and ^13^C NMR spectroscopy. Biological evaluation revealed that compounds **6** and **17** exhibited potent cytotoxic effects against T cell ALL (jurkat cells) and AML of promyelocytic subtype (APL) NB4 cells, with compound **17** showing IC_50_ values below 5 μM in both cell types. Compound **6** demonstrated selectivity for Jurkat cells. Further *in vitro* analyses, including apoptosis/cycle cell assays and pharmacokinetic predictions, confirmed their therapeutic potential. The data open new perspectives for “*in vivo*” studies concerning the application of quinazolin-4­(3*H*)-ones in treatment of acute leukemias of lymphoid and myeloid origins.

## Graphical abstract

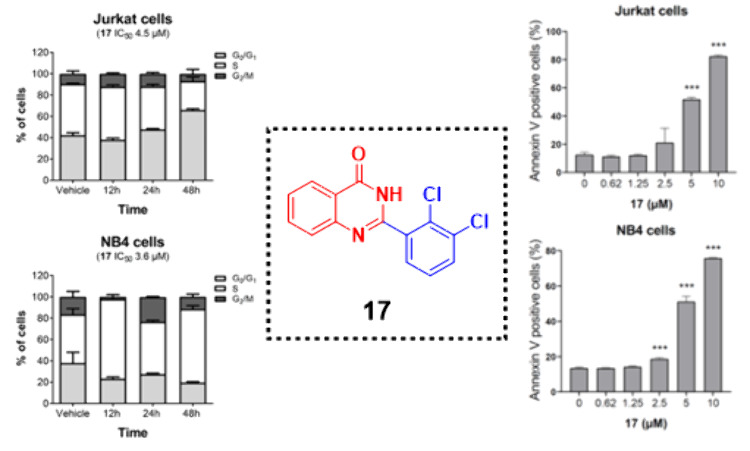

## Highlights

1. Twenty quinazolin-4­(3*H*)-ones were synthesized, four of which were unpublished.
2. Four compounds, **2**, **6**, **17**, and **20** showed cytotoxic activity in leukemia cells.
3. Compounds **6** and **17** were the most cytotoxic in Jurkat and NB4 cells, respectively.
4. Analysis of apoptosis showed that compound **17** was the most effective in Jurkat and NB4 cells.
5. The data indicate quinazolin-4-(3*H*)-ones as potential antileukemic agents.

## 1 Introduction

The population’s life expectancy has been increasing in recent years, surpassing the 70-year-old age group^1–3^ . At the same time, diseases such as neurodegenerative and cardiovascular disorders, diabetes and cancer are becoming increasingly common, raising global concerns about prevention and treatment.^4^ Therefore, one of the main challenges for science today is the molecular understanding of the mechanisms that induce cancer and the development and/or discovery of new substances with chemotherapeutic properties that eradicate or prevent the progression of this problematic disease.^5,6^

In the last two decades there has been a huge change in the way cancer is treated, from cytotoxic drugs to targeted drugs. The difference between these drugs is that targeted drugs can specifically target cancer cells and thus spare normal cells, ultimately with low toxicity and high potency. In the last 20 years, there has been a significant increase in targeted drugs approved by the FDA for the treatment of cancer^7,8^ .

Acute leukemias are aggressive malignancies characterized by the uncontrolled proliferation of hematopoietic progenitor cells in the bone marrow, leading to impaired production of normal blood cells. These cancers are broadly categorized into acute lymphoblastic leukemia (ALL) and acute myeloid leukemia (AML) and the incidence of ALL and AML are variable, with estimates in Western countries around 2.0 cases per 100.00 individuals (and 4 cases per 100,000 individuals annually),^9^ respectively. ALL represents about 20% of acute leukemia cases in adults and is associated with a poor prognosis, unlike the favorable outcomes often observed in pediatric ALL patients. The median age of AML patients at diagnosis is approximately 68 years, highlighting its predominance in older adults. Despite of progress in treatment strategies, outcomes remain particularly poor in elderly patients with acute leukemias, primarily due to reduced tolerance to aggressive therapies and the presence of multiple comorbid conditions.^10,11^

Given the variability of drugs used in chemotherapy, some problems arise during cancer treatment. It is important to note that the search for new antitumor drugs is based on the fact that cancer cells multiply much faster and have a higher replication rate than healthy cells (nontumor cells) and will suffer the cytotoxic effects of chemotherapy. However, chemotherapy is one of the main methods of treatment for cancer, but the effectiveness of the treatment is also limited by the fact that the drugs are resistant.

Nitrogen heterocycles have attracted the attention of researchers from various fields, with an extensive list of different biological activities. Among the heterocycles, quinazolines stand out, which have been widely investigated for the development of new drugs. It is important to note that this type of nucleus is and can be part of the pharmacophore for the development of new molecules. They have a huge range of different biological activities, such as anticancer,^12–15^ antimalarial,^16^ anti-inflammatory,^17,18^ antibacterial,^19^ antioxidant,^20^ antidiabetic,^21^ anticholinesterase activity,^22^ anti-β-secretase,^23^ anticonvulsant^24^ and antiviral.^25^

Figure 1 shows drugs with their respective biological targets. Quinazolines represent an extremely fascinating class of heterocyclic compounds that have attracted significant attention from researchers in the field of Medicinal Chemistry.

**Figure 1:**
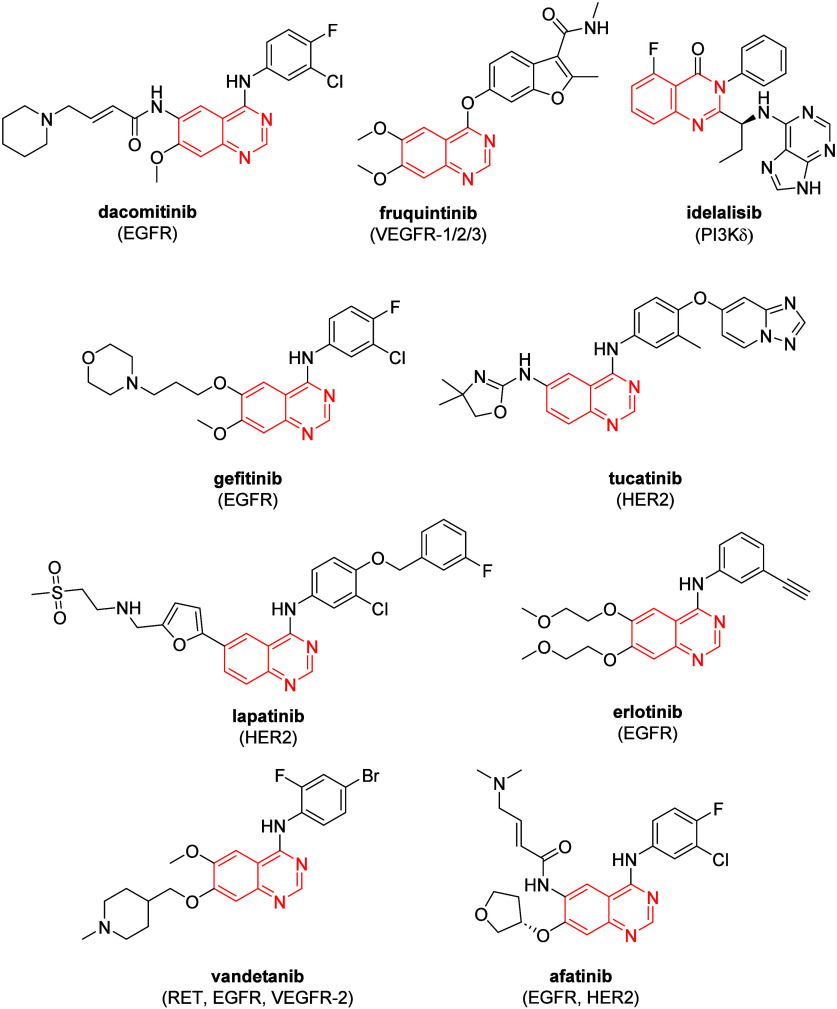
Drugs used in cancer treatment that have the quinazoline and quinazolin-4­(3*H*)-one rings.

In this paper, we present our contribution to the quinazoline class, reporting the synthesis, characterization and *in vitro* and *in silico* evaluations of 2-substituted quinazolin-4­(3*H*)-ones that can be said to be derivatives of quinazolines. This article describes the synthesis and biological evaluations for the potential discovery of molecules with potential antileukemic activity, given the importance of obtaining new anticancer agents. It is considerable to mention that quinazolines are effective anticancer agents and as only the drug idelalisb is a quinazolin-4­(3*H*)-one, the idea of searching for a new possible drug candidate based on this type of nucleus was considered. As far as we know and investigated, there have been no reports of this class of compounds being evaluated for acute leukemia cell lines, demonstrating that such compounds could become new agents against leukemia.

## 2 Materials and Methods

### 2.1 Synthesis of Compounds

The quinazolin-4­(3*H*)-ones substituted in position two were obtained by condensation between 2-aminobenzamide and different aldehydes. The reactions were carried out in dimethyl sulfoxide only. All reactions were heated to between 100 and 120 °C and conducted in an open flask.^26^

### 2.2 Cell Lines and Inhibitors

Jurkat cells were kindly provided by Prof. Sara Teresinha Olalla Saad (Hemocentro, State University of Campinas, Campinas, São Paulo, Brazil). NB4 cells were kindly provided by Prof. Eduardo Magalhães Rego (University of São Paulo, São Paulo, Brazil). Cells were cultured in RPMI-1640 medium supplemented with 10% fetal bovine serum (FBS, Sigma-Aldrich) according to the ATCC or DSMZ recommendations, plus 1% penicillin/streptomycin and maintained at 5% CO_2_ and 37 °C. All compounds were diluted to a 50 mM stock solution in DMSO.

### 2.3 Cell Viability Assay

In total 2 × 10^4^ cells per well were seeded in a 96-well plate in the appropriate medium in the presence of vehicle or different concentrations of compounds (ranged from 0.8 to 50 μM) for 72 h. For the compounds found to be of greatest interest (**6** and **17**), cells were exposed to the presence of vehicle or different concentrations of compounds (ranged from 0.8 to 50 μM) for 24, 48, and 72 h. Next, 10 μL methylthiazoletetrazolium (MTT, Sigma-Aldrich) solution (5 mg.mL^–1^) was added and incubated at 37 °C, 5% CO_2_ for 4 h. The reaction was stopped using 100 μL 0.1 N HCl in anhydrous isopropanol. Cell viability was evaluated by measuring the absorbance at 570 nm. IC_50_ values were calculated using nonlinear regression analysis in GraphPad Prism 5 (GraphPad Software, Inc., San Diego, CA, USA).^27,28^

### 2.4 Apoptosis Analysis

A total of 1 × 10^5^ cells per well were seeded in a 24-well plate in 10% FBS-containing RPMI-1640 medium in the presence of vehicle or **6** and **17** (0.6, 1.25, 2.5, 5, and 10 μM) for 48 h. The cells were then washed with ice-cold phosphate-buffered saline (PBS) and resuspended in a binding buffer containing 1 μg/mL propidium iodide and 1 μg.mL^–1^ APC-labeled annexin V. All specimens were analyzed by flow cytometry (FACSCalibur; Becton-Dickinson, San Jose, CA, USA) after incubation for 15 min at room temperature in a light-protected area. Ten thousand events were acquired for each sample.^27,28^

### 2.5 Cell-Cycle Analysis

A total of 2 × 10^6^ cells per well were seeded in a 24-well plate in RPMI-1640 medium containing 10% FBS in the presence of vehicle or **6** and **17** (with their respective IC_50_ μM) for 0, 12 24 and 48 h). Cells were fixed in 70% ethanol for at least 2 h at 4 °C and stained with 50 μg.mL^–1^ propidium iodide (PI) containing 10 μg.mL^–1^ RNaseA for 30 min at room temperature. Fluorescence analysis of cells was performed with a FACSCalibur (Becton-Dickinson, CA, USA). The resulting DNA distributions were analyzed by Modifit (Verify Software House Inc., ME, USA) for cell proportions during cell cycle phases.^29^

### 2.6 Statistical Analysis

Statistical analyses were performed using GraphPad Prism 8 (GraphPad Software, Inc., CA, USA). ANOVA and Bonferroni post-test was used for measurable factors. A value of *p* < 0.05 was considered statistically significant.

### 2.7 Pharmacokinetic and Physicochemical Properties Prediction

SwissADME and pkCSM software were used to predict the properties of the compounds.^30,31^ A wide list of properties was studied, such as permeability through Caco-2 cells, susceptibility to P-glycoprotein, inhibition of the principal CYP450 isoforms, gastrointestinal absorption and blood-brain barrier permeability. In addition, solubility, lipophilicity, molecular weight, number of rotatable bonds, number of hydrogen bond acceptors, number of hydrogen bond donors, molar refractivity and polar surface area. ProTox 3.0 software was used to predict the toxicity of the compounds.^32^

## 3 Results and Discussion

### 3.1 Synthesis and Characterization

There are various methodologies for the synthesis of quinazolin-4­(3*H*)-ones,^33–38^ but in this study we used a condensation between 2-aminobenzamide (anthranilamide) and different aldehydes, using dimethyl sulfoxide as solvent. The synthesis is shown in Figure 2.

**Figure 2:**
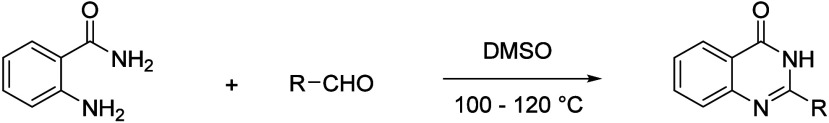
Schematic representation of the reaction to obtain 2-substituted quinazolin-4­(3*H*)-ones.

The products, **1**-**20**, showed low to excellent yields (26% to 94%). As the purification process is carried out by recrystallization in ethanol, the reason for the low yield of some products is due to loss during the process. It is important to note that this synthesis has a high atomic economy. Figure 3 shows the products with their respective yields. Compounds **11**, **16**, **19** and **20** are considered unpublished in scientific literature. The compounds that are not unpublished can be found in the literature cited and their spectroscopic data are consistent with those reported.^39–48^

**Figure 3:**
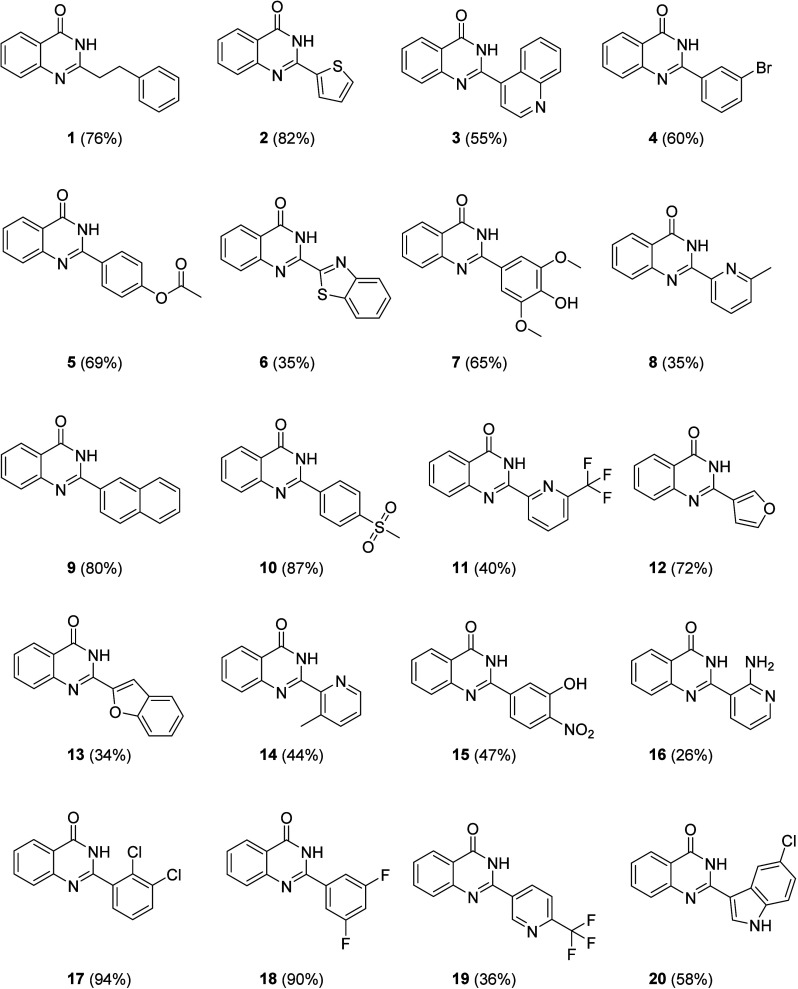
Structures and yields of the 2-substituted quinazolin-4­(3*H*)-ones obtained.

The main characteristic observed in the infrared spectra was the appearance of an absorption in the range between 1683 and 1657 cm^–1^, corresponding to the absorption of the amide carbonyl of the product. In the ^1^H NMR spectra, a singlet appears, absorbing in the region between 12.91 and 11.70 ppm, which is attributed to the NH hydrogen of the amide. In the ^13^C NMR spectra, the characteristic signal of the carbonyl amide was recorded in the range between 163.87 and 161.19 ppm (Figure 4). The other absorptions related to the other atoms depend on the substitutions and can be found in the experimental section. The spectra obtained for the 20 compounds synthesized can be found in the Supporting Information (Figures 1S to 80S).

**Figure 4:**
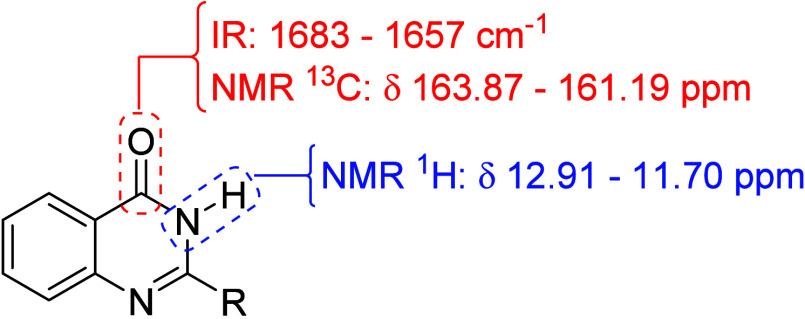
Range of values observed in IR, ^13^C NMR and ^1^H NMR spectra for the 2-substituted quinazolin-4-(3*H*)-ones.

### 3.2 Biological Evaluations *In Vitro*

#### 3.2.1 Cell Viability Assay Using MTT

Among the compounds synthesized, only four (**2**, **6**, **17**, and **20**) presented cytotoxic activity in acute leukemia cell lines (Table 1). Compound **20** exhibited IC_50_ values higher than 33.8 μM, and compound **2** exhibited IC_50_ values higher than 14.2 μM. In contrast, compound **6** showed IC_50_ of 1.9 and 49.4 μM in Jurkat and NB4 cells, respectively, which suggests a selectivity for Jurkat cells, showing a marked time-dependent reduction in viability. Exhibiting very important activity, compound **17** presented IC_50_ values of lower than 5 μM in both cell lines tested, and the effect was also time-dependent, but in this case 48 h of exposure was already very close to maximum potency and efficacy (Figure 5).

**Table 1:** Results of the Evaluation of the Effect of the Synthesized Compounds on the Proliferation of Cell Lines, Expressed in IC_50_ (μM)

	IC_50_ (μM)	
Compounds	Jurkat	NB4
**1**	>50	>50
**2**	14.2	16.6
**3**	>50	>50
**4**	>50	>50
**5**	>50	>50
**6**	1.9	49.4
**7**	>50	>50
**8**	>50	>50
**9**	>50	>50
**10**	>50	>50
**11**	>50	>50
**12**	>50	>50
**13**	>50	>50
**14**	>50	>50
**15**	>50	>50
**16**	>50	>50
**17**	4.5	3.6
**18**	>50	>50
**19**	>50	>50
**20**	42.8	33.8

**Figure 5:**
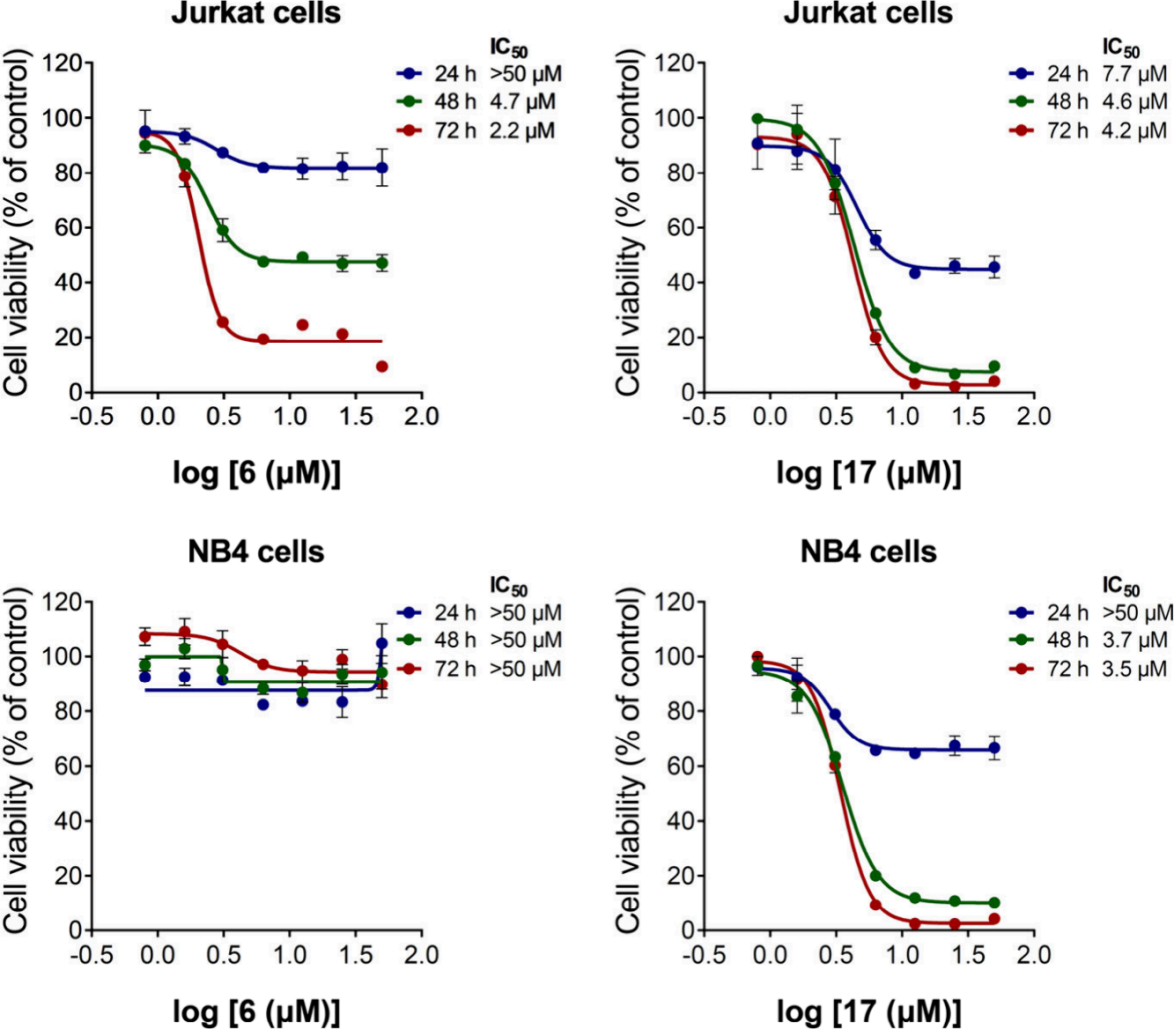
Concentration- and time-dependent response curves for compounds **6** and **17** in Jurkat and NB4 cells. The Jurkat and NB4 leukemic cell lines were used to investigate the concentration-dependent response by MTT, and the 50% inhibitory concentration (IC_50_) values are indicated for each cell line. The cells were treated with vehicle or with increasing concentrations of the compounds of interest for 24, 48, and 72 h. The results are expressed as a percentage in relation to the cells treated with vehicle and presented as a mean and standard deviation.

#### 3.2.2 Investigation of Apoptosis against Jurkat and NB4 Cells

The baseline cell viability was greater than 85%, which indicates a good quality cell culture and reliability in the data obtained. Compound **6** showed greater efficacy, but still limited in Jurkat cells compared to NB4 cells. Compound **17** was the most effective in both models tested (Figure 6).

**Figure 6:**
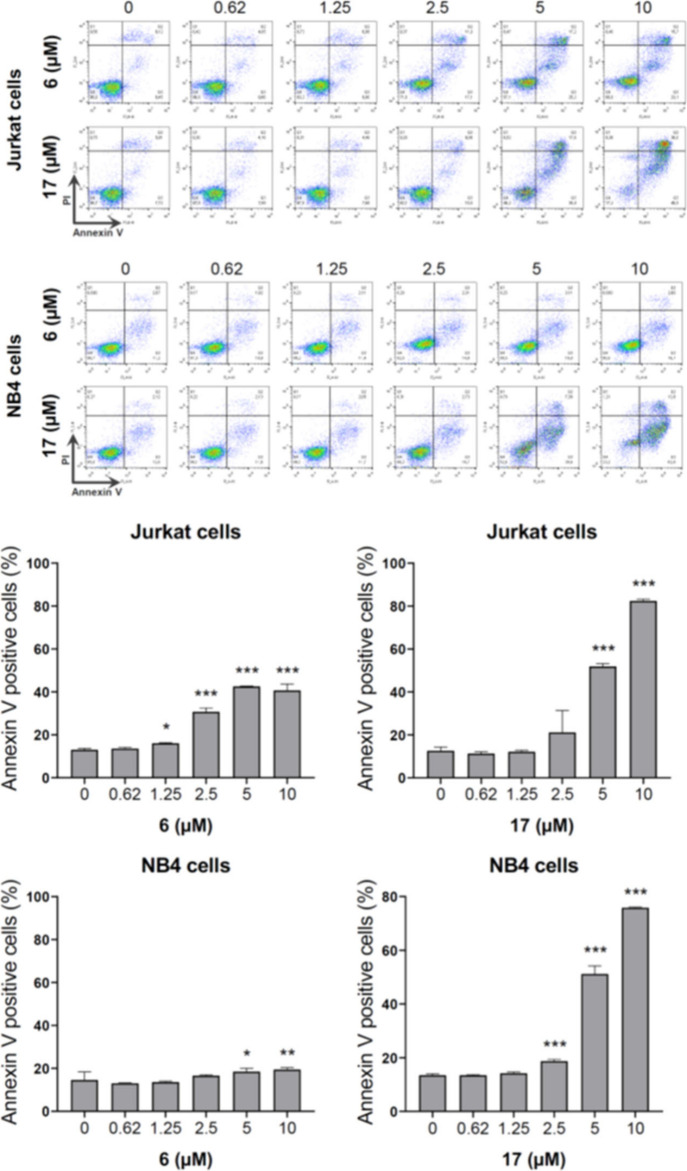
**17** is highly effective at inducing apoptosis in Jurkat and NB4 cells. Apoptosis was measured by flow cytometry in Jurkat and NB4 cells treated with either vehicle or increasing concentrations of **6** or **17** (ranging from 0.6 to 10 μM) for 48 h. Representative dot plots are shown for each condition. The upper and lower right quadrants (Q2 plus Q3) cumulatively represent the apoptotic cell population (annexin V+ cells). Bar graphs show the mean ± SD of at least three independent experiments. The p-values and cell lines are indicated in the graphs; *p < 0.05, **p < 0.01, ***p < 0.0001; ANOVA with Bonferroni post-test.

#### 3.2.3 Cell Cycle Analysis

The graphs show the distribution of cells in different phases of the cell cycle (G0/G1, S and G2/M) after treatment with the compounds **6** and **17** against Jurkat and NB4 leukemia cell lines. Compound **6** versus Jurkat (A) had a significant increase in G2/M, suggesting a mitotic arrest. Compound **6** versus NB4 (B) promoted a robust effect in G2/M in 48 h, but with a high IC50 (49.4 μM) suggesting that the drug has relevant effects, but a low potency and the cell has high tolerance to damage. Compound **17** when tested against Jurkat (C) caused an accumulation of G0/G1 and suggests that **17** prevents the G1/S transition with the suggested IC_50_ (4.5 μM). This same compound when placed against NB4 (D) demonstrates a blocking effect in S phase preventing replication. The graphs demonstrate that while compound **6** promotes an increase in the G2/M phase in cells, compound **17** promotes a distinct effect against Jurkat and NB4 cells (Figure 7).

**Figure 7:**
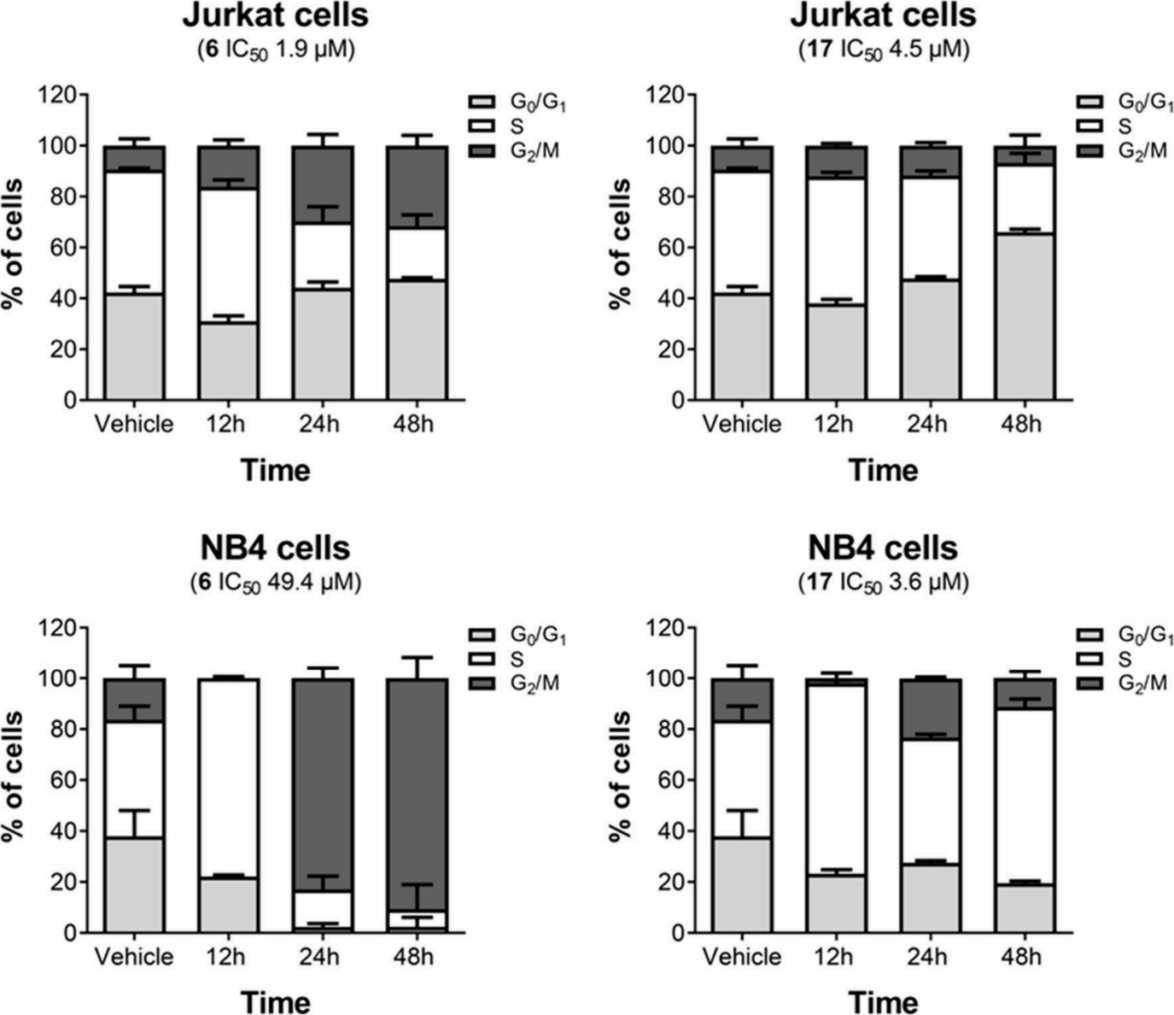
Data expressed in these graphs are the results of 3 distinct experiments analyzed at 3 different times (12, 24, and 48 h) and both were subjected to ANOVA statistical analysis with Bonferroni post-test and the error bars are SD *p <* 0.05 for controls and *p* < 0.001 for the other bars.

### 3.3 Pharmacokinetic and Physicochemical Properties Prediction

Initially, molecular properties were analyzed, such as molecular weight, number of rotatable bonds, number of hydrogen bond acceptors, number of hydrogen bond donors, molar refractivity, and topological polar surface area. In addition, physicochemical parameters such as Log S and Log P were also analyzed. For the prediction of these properties, the SwissADME^30^ software is widely used as a tool within Medicinal Chemistry, and these *in silico* studies are very important in the development of new drugs. Figure 8 shows the compounds synthesized and the drugs discussed below. The results obtained for the 2-substituted quinazolin-4­(3*H*)-ones, which showed antileukemic activity, are presented in Table 2.

**Figure 8:**
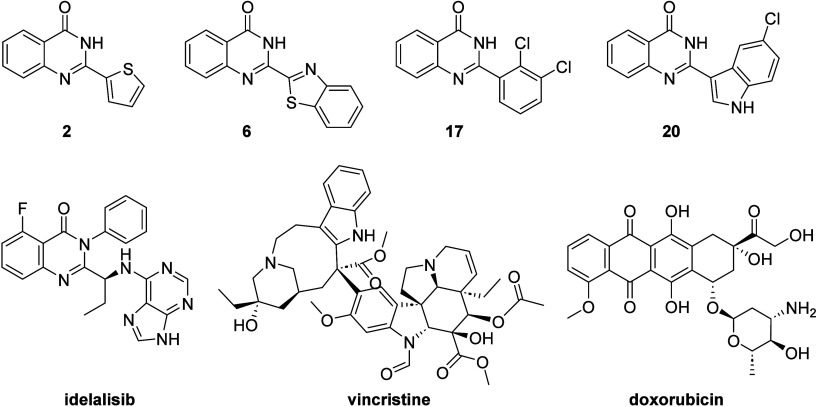
Compounds **2**, **6**, **17** and **20** showed antileukemic activity against the Jurkat and NB4 cell lines. The drugs idelalisib, vincristine and doxorubicin were used for property prediction studies.

**Table 2:** Prediction of the Molecular Descriptors of 2-Substituted Quinazolin-4­(3*H*)-ones and Some Drugs Used in the Treatment of Leukemia

Compound	MW^b^	NRB^c^	HBD^d^	HBA^e^	MR^f^	TPSA (Å)^g^	Log S^h^	Log P[Table-fn t2fn9]
**2**	228.27	1	1	2	65.68	73.99	–3.67	2.84
**6**	279.32	1	1	3	80.98	86.88	–4.19	3.20
**17**	291.13	1	1	2	77.82	45.75	–4.78	3.79
**20**	295.72	1	2	2	84.67	61.54	–4.24	3.33
**idelalisib**	415.42	5	2	6	115.95	101.38	–5.01	3.28
**vincristine**	824.96	11	3	12	233.11	171.17	–6.39	3.40
**doxorubicin**	543.52	5	6	12	132.66	206.07	–3.91	0.50

a MW: molecular weight.

b NRB: number of rotatable bonding.

c HBD: hydrogen bond donors.

d HBA: hydrogen bond acceptors.

e MR: molar refractivity.

f TPSA: topological polar surface area.

g Log S: logarithm of solubility (ESOL).

h Log P: average of the logarithm of the partition coefficient (Consensus).

Idelalisib, vincristine and doxorubicin are drugs used in the treatment of leukemia and different types of cancer. These drugs were included in this study to predict their properties for comparative purposes between the compounds under investigation. Druglikeness is a concept that expresses how similar a substance is to a drug in terms of bioavailability requirements if taken orally.^49^ A number of filters are used to analyze this Druglikeness, such as Lipinski, Ghose, Veber, Egan and Muegge. Initially, when we analyzed the 2-substituted quinazolin-4­(3*H*)-ones, none of them showed any type of violation of these filters used, and the drug Idelalisib also showed no violation. However, when we analyzed the drugs vincristine and doxorubicin, both had all the possible violations of these Druglikeness filters, such as molar mass greater than 480 g·mol^–1^, number of rotatable bonds greater than 10 and topological polar surface area greater than 140 Å^2^.

After examining the Log S values, **2** and doxorubicin were classified soluble, with **2** exhibiting more soluble than doxorubicin. **6**, **17**, **20** and idelalisib were considered moderately soluble, whereas vincristine was considered poorly soluble. Moreover, the poor solubility of vincristine may result in significant limitations to its oral bioavailability, an aspect that has, in fact, not been extensively addressed in the literature. Regarding the Log P (consensus) values, all the molecules fall within the acceptable range of molecular filters, with compound **17** displaying the highest value (3.79).

Molar refractivity could affect the bioavailability and pharmacokinetic performance of compounds. Therefore, compounds that have a higher molar refractivity may have a greater tendency to interact with plasma proteins (such as albumin), which can affect their distribution in the body, since binding to proteins can often limit the amount of the compound that is free and available for action. In addition, the drugs used in the *in silico* study have a higher molar refractivity than the synthesized compounds that showed antileukemic activity, since compounds that have a high molar refractivity can interact more strongly with enzyme systems or be more susceptible to conjugation reactions, which can alter their elimination from the body.

The final analysis of all those described in this study indicates that all the quinazolin-4­(3*H*)-ones synthesized meet the parameters for oral bioavailability.

Furthermore, other properties were measured, such as gastrointestinal absorption (GI a), blood-brain barrier permeability (BBB p), inhibitor or substrate for P-glycoprotein (P-gp) and inhibition of the main CYP450 isoforms (1A2, 2C9, 2C19, 2D6 and 3A4), as well as permeability through Caco-2 cells (human adenocarcinoma cell line, Log P_app_ in 10^–6^ cm.s^–1^) which was calculated using pkCSM (Table 3).^31^

**Table 3:** *In Silico* Pharmacokinetic Profile of the Synthesized 2-Substituted Quinazolin-4­(3*H*)-ones Compared with the Drugs Idelalisib, Vincristine and Doxorubicin

Compound	CYP isoforms inhibition	GI a	BBB p	P-gp	Caco-2 p
**2**	1A2	High	Yes	No	1.217
**6**	1A2, 2C9	High	No	No	1.235
**17**	1A2, 2C19, 2C9	High	Yes	No	1.271
**20**	1A2	High	Yes	No	1.188
**idelalisib**	2C9, 2D6	High	No	Yes	0.136
**vincristine**	3A4	Low	No	Yes	0.69
**doxorubicin**	-	Low	No	Yes	0.6

All the compounds, **2**, **6**, **17** and **20**, showed high gastrointestinal absorption, in addition to the drug idelalisb, while vincristine and doxorubicin showed low gastrointestinal absorption. Quinazolin-4­(3*H*)-ones were not expected to be P-glycoprotein substrates, which is a positive feature, unlike the drugs used as standards in the *in silico* study. All the compounds, in addition to the drugs, showed positive values for permeability through Caco-2 cells, where a positive value indicates that a compound is well absorbed. In addition to the drugs, **6** did not show the possibility of permeability at the blood-brain barrier, which may be a problem for compounds **2**, **17** and **20**, which were positive. When we look at the inhibition of the CYP450 isoforms, it is clear that the compounds synthesized (**2**, **6**, **17** and **20**) can inhibit the 1A2 isoform. Doxorubicin was the only compound in the study that did not show possible inhibition of CYP450 isoforms. The drugs idealisib, vincristine and doxorubin all have TPSA values over 100 Å, making it difficult for compounds with high TPSA values to cross the blood-brain barrier. Since vincristine and doxorubicin have higher values than idelalisb, this may influence limitations in permeability, especially considering the oral pathway.

Overall, solubility plays a key role in gastrointestinal absorption and, consequently, in the oral bioavailability of drugs. Compounds with low solubility usually dissolve poorly in the gastrointestinal tract, reducing the amount available for absorption through the intestinal lining. Therefore, even when a compound has good permeability, low solubility can greatly limit its bioavailability. This may help explain, at least in part, the low gastrointestinal absorption observed for vincristine and doxorubicin, despite their favorable permeability properties.^50–52^

In addition to all the predictions mentioned previously, predictions of the toxicity of the same previous compounds were also made. This *in silico* toxicity prediction of compounds is an important part of the new drug development process. Studies like this can help to reduce the number of *in vivo* studies and also in determining toxic doses. Table 4 describes some targets for organ toxicity and toxicological end points.

**Table 4:** Prediction of *In Silico* Toxicity for 2-Substituted Quinazolin-4­(3*H*)-ones and Selected Drugs^a^

	Target																	
	Hepatot^d^		Neurot^e^		Nephrot^f^		Resp t^g^		Cardiot^h^		Carcinog^i^		Immunot^j^		Mutagen[Table-fn t4fn11]		Citotox[Table-fn t4fn12]	
Compound	Pred	Pred	Pred	Prob	Pred	Pred	Pred	Prob	Pred	Prob	Pred	Prob	Pred	Prob	Pred	Prob	Pred	Prob
**2**	+	65	+	62	–	63	+	53	–	90	–	55	–	99	–	69	–	89
**6**	++	74	+	54	–	62	+	54	–	91	–	59	–	99	–	62	–	87
**17**	–	53	++	85	–	70	–	52	–	95	–	53	–	82	–	72	–	92
**20**	+	56	++	76	–	56	–	62	–	93	+	53	–	84	–	67	–	90
**idelalisib**	+	57	++	95	–	66	++	88	–	92	–	54	–	79	+	54	–	74
**vincristine**	–	80	++	86	+	66	++	97	–	81	–	55	++	99	–	95	++	94
**doxorubicin**	–	86	++	74	++	80	++	91	+	64	–	90	++	99	++	98	++	94

a The more positive (++), the more likely it is to be toxic, and the more negative (−), the more likely it is not to be toxic. Considering all the compounds, the small molecule **17** could be considered the least toxic, and doxorubicin would be considered the most toxic. Pred: Prediction; Prob: Probability (%). Prediction [active (++ very active; + little active) or inactive (− very inactive; – little inactive)].

b Hepatot: Hepatotoxicity.

c Neurot: Neurotoxicity.

d Nephrot: Nephrotoxicity.

e Resp t: Respiratory toxicity.

f Cardiot: Cardiotoxicity.

g Carcinog: Carcinogenicity.

h Immunot: Immunotoxicity.

i Mutagen: Mutagenicity.

j Citotox: Citotoxicity.

It is interesting to note that the quinazolin-4­(3*H*)-ones showed similar organ toxicity targets and toxicological end points. The only two exceptions were for compound **2** and **6**, which may exhibit hepatotoxicity and respiratory toxicity. In contrast, compound **17** showed only the possibility of being hepatotoxic, as did all the other compounds with activity in the study, and also the drugs used as controls in this *in silico* test. Therefore, **17** exhibits good molecular, pharmacokinetic and toxicological properties.

## 4 Conclusions

In this study, we prepared a series of 2-substituted quinazolin-4­(3*H*)-ones that exhibited antiproliferative activities in T cell ALL (Jurkat cells) and APL (NB4 cells). Of the 20 compounds synthesized for this study, compounds **11**, **16**, **19** and **20** are considered unpublished in the scientific literature. The most promising result of the study was quinazolin-4­(3*H*)-one **17**, derived from 2,3-dichlorobenzaldehyde, with IC_50_ values of 4.5 and 3.6 μM for both acute leukemias Jurkat and NB4 cells, respectively. In the analysis of apoptosis by flow cytometry, the highlight was also compound **17**, which was the most effective in both cell lines. In the analysis of the cell cycle by flow cytometry, in brief, compound **17** exhibited an accumulation of G1, suggesting that the compound prevents the G1/S transition in Jurkat cells, in counterpart, in NB4 cells, a blocking effect was demonstrated in the S phase, causing replication to be prevented. The prediction of the properties proposed in this study suggests that compound **17** is very promising, with the only problem being the possibility of it causing hepatotoxicity. Given the potential success of quinazolin-4­(3*H*)-one ring compounds as new antileukemic agents, further and subsequent *in vitro* studies are needed to determine the mechanisms that conferred selectivity of compound **6** against T-type T cell ALL, and to evaluate the selectivity of compound **17** in T cell ALL and APL. The data obtained in this article demonstrate the need for further *in vitro* studies to determine the biological target(s) of the molecules with the greatest antileukemic potential. Studies *in vivo* in animal models of acute leukemias are also required for evaluations of efficacy and safety of the agents.

## 5 Experimental Section

### 5.1 General

The starting materials were purchased from commercial sources in high purity. The reactions were monitored by thin layer chromatography (TLC) and the plates were revealed by UV–vis. NMR spectra were recorded on BRUKER equipment operating at 400 or 500 MHz for ^1^H and 100 or 125 MHz ^13^C. Chemical shifts were given in ppm. Infrared spectra were acquired on an Agilent CARY 630 FTIR IR absorption spectrophotometer using an ATR accessory. The high-resolution spectra were acquired on a Thermo QExactive Orbitrap analyzer, without a column. The samples were injected with the following experimental parameters: positive polarity, FIA flow rate 200 μL.min^–1^ acetonitrile:water, 1:1 v/v with 0.1% formic acid, resolution 140 10E3, injection volume 20 μL, analysis time 2 min and cone voltage 3.5 kV and 50 V SLens.

### 5.2 General Procedure for the Preparation of 2-Substituted Quinazolin-4­(3H)-ones

The 2-substituted quinazolin-4­(3*H*)-ones were obtained through a condensation reaction between 2-aminobenzamide and different aldehydes, according to the following procedure.

To a round-bottomed flask, 2 mmol of 2-aminobenzamide and 2.4 mmol of aldehyde were added, and 15 mL of dimethyl sulfoxide was added. The resulting solution, in an open flask under magnetic stirring and at a temperature of 100 °C, was monitored by thin layer chromatography. At the end of the reaction, the mixture was cooled to room temperature and 100 mL of water were added and the precipitate formed. The reaction mixture was filtered through a Büchner funnel and the product was recrystallized in ethanol. After being collected, the product was dried at room temperature and pressure, and then under vacuum. All products were obtained as solids.

### 5.3 Characterization

The compounds were characterized by infrared spectroscopy (IR), nuclear magnetic resonance (^1^H and ^13^C NMR) and high-resolution mass spectrometry (HRMS). NMR spectra were obtained with a Bruker instrument operating in the range of 400 to 500 MHz for ^1^H and ^13^C, respectively. Mass spectra analysis was performed through TOF-ESI mass spectrometry. Compounds that are not novel in the scientific literature are accompanied by the corresponding reference where their characterization can be found. It is worth noting that, for all non-novel compounds, the obtained characterization is consistent with that reported in the literature.

#### 5.3.1 2-Phenethylquinazolin-4­(3*H*)-one

Obtained as a white solid. 76% yield. The compound is known in the literature.^39^

IR (ATR, v_máx_, cm^–1^): 3170 (νNH, amide), 1678 (νCO, amide), 1613 (δNH, amide). ^1^H NMR (400 MHz, DMSO-*d*_6_, ppm): δ 12.26 (s, 1H, H-amide), 8.09 (dd, *J* = 8.0, 1.5 Hz, 1H, H-arom), 7.79 (ddd, *J* = 8.5, 7.1, 1.6 Hz, 1H, H-arom), 7.63 (dd, *J* = 8.4, 1.1 Hz, 1H, H-arom), 7.47 (ddd, *J* = 8.1, 7.1, 1.2 Hz, 1H, H-arom), 7.29 (d, *J* = 5.1 Hz, 4H, H-arom), 7.22 – 7.18 (m, 1H, H-arom), 3.10 – 3.03 (m, 2H, CH_2_), 2.94 – 2.87 (m, 2H, CH_2_). ^13^C NMR (125 MHz, DMSO-*d*_6_, ppm): δ 162.25, 157.06, 149.34, 141.24, 134.80, 128.85, 128.82, 127.30, 126.56, 126.51, 126.18, 121.32, 36.80, 32.95. HRMS (ESI^+^) calculated for C_16_H_15_N_2_O^+^ [M + H]^+^: 251.1179. Found: 251.1176.

#### 5.3.2 2-(Thiophen-2-yl)­quinazolin-4­(3*H*)-one

Obtained as a pale yellow solid. 82% yield. The compound is known in the literature.^39^

IR (ATR, v_máx_, cm^–1^): 3170 (νNH, amide), 1667 (νCO, amide), 1587 (δNH, amide). ^1^H NMR (400 MHz, DMSO-*d*_6_, ppm): δ 12.66 (s, 1H, H-amide), 8.24 (dd, *J* = 3.9, 1.2 Hz, 1H, H-arom), 8.13 (dd, *J* = 8.0, 1.5 Hz, 1H, H-arom), 7.88 (dd, *J* = 5.1, 1.1 Hz, 1H, H-arom), 7.81 (ddd, *J* = 8.6, 7.1, 1.6 Hz, 1H, H-arom), 7.70 – 7.63 (m, 1H, H-arom), 7.50 (ddd, *J* = 8.1, 7.1, 1.2 Hz, 1H, H-arom), 7.25 (dd, *J* = 5.0, 3.8 Hz, 1H, H-arom). ^13^C NMR (125 MHz, DMSO-*d*_6_, ppm): δ 162.26, 149.1), 148.30, 137.84, 135.17, 132.65, 129.88, 128.98, 127.43, 126.81, 126.46, 121.36. HRMS (ESI^+^) calculated for C_12_H_9_N_2_OS^+^ [M + H]^+^: 229,0430. Found: 229,0427.

#### 5.3.3 2-(Quinolin-4-yl)­quinazolin-4­(3*H*)-one

Obtained as a white solid. 55% yield. The compound is known in the literature.^40^

IR (ATR, v_máx_, cm^–1^): 3159 (νNH, amide), 1678 (νCO, amide), 1607 (δNH, amide). ^1^H NMR (400 MHz, DMSO-*d*_6_, ppm): δ 12.84 (s, 1H, H-amide), 9.08 (d, *J* = 4.4 Hz, 1H, H-arom), 8.27 – 8.21 (m, 2H, H-arom), 8.16 (d, *J* = 8.4 Hz, 1H, H-arom), 7.93 – 7.84 (m, 2H, H-arom), 7.84 – 7.75 (m, 2H, H-arom), 7.71 – 7.60 (m, 2H, H-arom). ^13^C NMR (125 MHz, DMSO-*d*_6_, ppm): δ 162.15, 152.10, 150.56, 148.90, 148.44, 139.62, 135.16, 130.42, 129.91, 128.11, 128.09, 127.78, 126.40, 126.05, 125.17, 122.03, 121.98. HRMS (ESI^+^) calculated for C_17_H_12_N_3_O^+^ [M + H]^+^: 274.0975. Found: 274.0974.

#### 5.3.4 2-(3-Bromophenyl)­quinazolin-4­(3*H*)-one

Obtained as a white solid. 60% yield. The compound is known in the literature.^39^

IR (ATR, v_máx_, cm^–1^): 3173 (νNH, amide), 1674 (νCO, amide), 1607 (δNH, amide). ^1^H NMR (400 MHz, DMSO-*d*_6_, ppm): δ 12.63 (s, 1H, H-amide), 8.39 (t, *J* = 1.9 Hz, 1H, H-arom), 8.23 – 8.15 (m, 2H, H-arom), 7.86 (ddd, *J* = 8.4, 7.0, 1.6 Hz, 1H, H-arom), 7.83 – 7.75 (m, 2H, H-arom), 7.58 – 7.50 (m, 2H, H-arom). ^13^C NMR (125 MHz, DMSO-*d*_6_, ppm): δ 162. 56, 151.42, 148.95, 135.42, 135.18, 134.53, 131.25, 130.88, 128.10, 127.42, 127.27, 126.36, 122.38, 121.63. HRMS (ESI^+^) calculated for C_14_H_10_BrN_2_O^+^ [M + H]^+^: 300.9971. Found: 300.9968.

#### 5.3.5 4-(4-Oxo-3,4-dihydroquinazolin-2-yl)­phenyl Acetate

Obtained as a white solid. 69% yield. The compound is known in the literature.^47^

IR (ATR, v_máx_, cm^–1^): 3173 (νNH, amide), 1762 (νCO, ester), 1657 (νCO, amide), 1605 (δNH, amide). ^1^H NMR (400 MHz, DMSO-*d*_6_, ppm): δ 12.58 (s, 1H, H-amide), 8.23 (d, *J* = 8.7 Hz, 2H, H-arom), 8.17 (dd, *J* = 7.9, 1.5 Hz, 1H, H-arom), 7.85 (ddd, *J* = 8.6, 7.1, 1.6 Hz, 1H, H-arom), 7.75 (dd, *J* = 8.3, 1.1 Hz, 1H, H-arom), 7.53 (ddd, *J* = 8.1, 7.0, 1.2 Hz, 1H, H-arom), 7.35 – 7.31 (m, 2H, H-arom), 2.32 (s, 3H, CH_3_). ^13^C NMR (125 MHz, DMSO-*d*_6_, ppm): δ 169.48, 162.69, 153.35, 152.14, 149.17, 135.11, 130.74, 129.72, 127.96, 127.10, 126.34, 122.58, 121.41, 21.38. HRMS (ESI^+^) calculated for C_16_H_13_N_2_O_3_^+^ [M + H]^+^: 281.0921. Found: 281.0919.

#### 5.3.6 2-(Benzo­[*d*]­thiazol-2-yl)­quinazolin-4­(3*H*)-one

Obtained as a butter-yellow solid. 35% yield. The compound is known in the literature.^40^

IR (ATR, v_máx_, cm^–1^): 3136 (νNH, amide), 1683 (νCO, amide), 1590 (δNH, amide). ^1^H NMR (400 MHz, DMSO-*d*_6_, ppm): δ 12.82 (s, 1H, H-amide), 8.26 (dd, *J* = 8.1, 1.4 Hz, 1H, H-arom), 8.20 (t, *J* = 6.7 Hz, 2H, H-arom), 7.94 – 7.87 (m, 1H, H-arom), 7.83 (d, *J* = 7.9 Hz, 1H, H-arom), 7.68 – 7.60 (m, 3H, H-arom). ^13^C NMR (125 MHz, DMSO-*d*_6_, ppm): δ 162.76 (CO), 161.14 (C-arom), 153.35 (C-arom), 148.18 (C-arom), 147.23 (CH-arom), 136.45 (CH-arom), 135.37 (CH-arom), 128.49 (CH-arom), 128.08 (CH-arom), 127.67 (2xC-arom), 126.79 (CH-arom), 124.45 (CH-arom), 123.35 (CH-arom), 123.06 (C-arom). HRMS (ESI^+^) calculated for C_15_H_10_N_3_OS^+^ [M + H]^+^: 280.0539. Found: 280.0537.

#### 5.3.7 2-(4-Hydroxy-3,5-dimethoxyphenyl)­quinazolin-4­(3*H*)-one

Obtained as a banana yellow solid. 65% yield. The compound is known in the literature.^41^

IR (ATR, v_máx_, cm^–1^): 3404 (νOH, phenol), 3181 (νNH, amide), 1667 (νCO, amide), 1581 (δNH, amide). ^1^H NMR (400 MHz, DMSO-*d*_6_, ppm): δ 12.40 (s, 1H, H-amide), 9.15 (s, 1H, OH), 8.14 (dd, *J* = 8.1, 1.5 Hz, 1H, H-arom), 7.82 (ddd, *J* = 8.5, 7.0, 1.6 Hz, 1H, H-arom), 7.72 (d, *J* = 7.2 Hz, 1H, H-arom), 7.59 (s, 2H), 7.52 – 7.45 (m, 1H), 3.89 (s, 6H, CH_3_). ^13^C NMR (125 MHz, DMSO-*d*_6_, ppm): δ 162,85, 152.43, 149.40, 148.29, 139.53, 135.01, 127.74, 126.48, 126.30, 122.57, 121.08, 105.77, 56.64. HRMS (ESI^+^) calculated for C_16_H_15_N_2_O_4_^+^ [M + H]^+^: 299.1026. Found: 299.1023.

#### 5.3.8 2-(6-Methylpyridin-2-yl)­quinazolin-4­(3*H*)-one

Obtained as a white solid. 35% yield. The compound is known in the literature.^40^

IR (ATR, v_máx_, cm^–1^): 3322 (νNH, amide), 1677 (νCO, amide), 1614 (δNH, amide). ^1^H NMR (400 MHz, DMSO-*d*_6_, ppm): δ 11.70 (s, 1H, H-amide), 8.27 (d, *J* = 7.8 Hz, 1H, H-arom), 8.21 (dd, *J* = 7.7, 1.2 Hz, 1H, H-arom), 7.97 (t, *J* = 7.8 Hz, 1H, H-arom), 7.89 (ddd, *J* = 8.5, 7.0, 1.6 Hz, 1H, H-arom), 7.81 (d, *J* = 7.7 Hz, 1H, H-arom), 7.61 – 7.55 (m, 1H, H-arom), 7.53 (d, *J* = 7.7 Hz, 1H, H-arom), 2.64 (s, 3H, CH_3_). ^13^C NMR (125 MHz, DMSO-*d*_6_, ppm): δ 161.20, 158.3), 150.34, 149.04, 148.18, 138.64, 135.22, 128.20, 127.70, 126.59, 122.46, 119.58, 24.19. HRMS (ESI^+^) calculated for C_14_H_12_N_3_O^+^ [M + H]^+^: 238.0975. Found: 238.1033.

#### 5.3.9 2-(Naphthalen-2-yl)­quinazolin-4­(3*H*)-one

Obtained as a white solid. 80% yield. The compound is known in the literature.^42^

IR (ATR, v_máx_, cm^–1^): 3136 (νNH, amide), 1672 (νCO, amide), 1605 (δNH, amide). ^1^H NMR (500 MHz, DMSO-*d*_6_, ppm): δ 12.67 (s, 1H, H-amide), 8.83 (s, 1H, H-arom), 8.32 (dd, *J* = 8.6, 1.9 Hz, 1H, H-arom), 8.20 (dd, *J* = 8.0, 1.5 Hz, 1H, H-arom), 8.08 (t, *J* = 7.6 Hz, 2H, H-arom), 8.05 – 8.01 (m, 1H, H-arom), 7.88 (ddd, *J* = 8.5, 7.6, 1.6 Hz, 1H, H-arom), 7.81 (dd, *J* = 8.3, 1.2 Hz, 1H, H-arom), 7.68 – 7.62 (m, 2H, H-arom), 7.58 – 7.54 (m, 1H, H-arom). ^13^C NMR (125 MHz, DMSO-*d*_6_, ppm): δ 162.71, 152.72, 149.26, 135.15, 134.62, 132.77, 130.43, 129.44, 128.66, 128.59, 128.41, 128.15, 128.05, 127.40, 127.16, 126.39, 124.98, 121.54. HRMS (ESI^+^) calculated for C_18_H_13_N_2_O^+^ [M + H]^+^: 273.1022. Found: 273.1019.

#### 5.3.10 2-(4-(Methylsulfonyl)­phenyl)­quinazolin-4­(3*H*)-one

Obtained as a white solid. 87% yield. The compound is known in the literature.^45^

IR (ATR, v_máx_, cm^–1^): 3198 (νNH, amide), 1661 (νCO, amide), 1601 (δNH, amide). ^1^H NMR (500 MHz, DMSO-*d*_6_, ppm): δ 12.78 (s, 1H, H-amide), 8.44 – 8.39 (m, 2H, H-arom), 8.19 (dd, *J* = 7.9, 1.5 Hz, 1H, H-arom), 8.14 – 8.08 (m, 2H, H-arom), 7.89 (ddd, *J* = 8.5, 7.1, 1.6 Hz, 1H, H-arom), 7.80 (d, *J* = 7.6 Hz, 1H, H-arom), 7.62 – 7.55 (m, 1H, H-arom), 3.32 (s, 3H, CH_3_). ^13^C NMR (125 MHz, DMSO-*d*_6_, ppm): δ 162.57, 151.55, 148.89, 143.40, 137.77, 135.24, 129.30, 128.20, 127.69, 127.63, 126.39, 121.71, 43.75. HRMS (ESI^+^) calculated for C_15_H_13_N_2_O_3_S^+^ [M + H]^+^: 301.0641. Found: 301.0636.

#### 5.3.11 2-(6-(Trifluoromethyl)­pyridin-2-yl)­quinazolin-4­(3*H*)-one

Obtained as a white solid. 40% yield. The compound is novel in the scientific literature.

IR (ATR, v_máx_, cm^–1^): 3198 (νNH, amide), 1669 (νCO, amide), 1605 (δNH, amide). ^1^H NMR (500 MHz, DMSO-*d*_6_, ppm): δ 12.00 (s, 1H, H-amide), 8.68 (d, J = 8.0 Hz, 1H, H-arom), 8.36 (t, J = 7.9 Hz, 1H, H-arom), 8.25 – 8.19 (m, 1H, H-arom), 8.16 (d, J = 7.7 Hz, 1H, H-arom), 7.93 – 7.87 (m, 1H, H-arom), 7.83 (d, J = 9.3 Hz, 1H, H-arom), 7.64 – 7.59 (m, 1H, H-arom). ^13^C NMR (125 MHz, DMSO-*d*_6_, ppm): δ 161.42, 150.20, 149.56, 148.58, 146,36 (J = 35 Hz), 140.90, 135.28, 128.32, 128.17, 126.61, 126.15, 123.71, 123.69, 122.57, 121,73 (J = 273,8 Hz). HRMS (ESI^+^) calculated for C_14_H_9_F_3_N_3_O^+^ [M + H]^+^: 292.0692. Found: 292.0724.

#### 5.3.12 2-(Furan-3-yl)­quinazolin-4­(3*H*)-one

Obtained as a dark mustard yellow solid. 72% yield. The compound is known in the literature.^42^

IR (ATR, v_máx_, cm^–1^): 3132 (νNH, amide), 1674 (νCO, amide), 1605 (δNH, amide). ^1^H NMR (400 MHz, DMSO-*d*_6_, ppm): δ 12.42 (s, 1H, H-amide), 8.64 (s, 1H, H-arom), 8.13 (d, *J* = 7.9 Hz, 1H, H-arom), 7.87 (s, 1H, H-arom), 7.82 (t, *J* = 7.6 Hz, 1H, H-arom), 7.67 (d, *J* = 8.1 Hz, 1H, H-arom), 7.50 (t, *J* = 7.5 Hz, 1H, H-arom), 7.17 (s, 1H, H-arom). ^13^C NMR (125 MHz, DMSO-*d*_6_, ppm): δ 162.38, 149.34, 147.70, 145.54, 145.23, 135.06, 127.55, 126.76, 126.35, 121.94, 121.46, 109.54. HRMS (ESI^+^) calculated for C_12_H_9_N_2_O_2_^+^ [M + H]^+^: 213.0659. Found: 213.0656.

#### 5.3.13 2-(Benzofuran-2-yl)­quinazolin-4­(3*H*)-one

Obtained as a banana yellow solid. 34% yield. The compound is known in the literature.^48^

IR (ATR, v_máx_, cm^–1^): 3175 (νNH, amide), 1678 (νCO, amide), 1602 (δNH, amide). ^1^H NMR (500 MHz, DMSO-*d*_6_, ppm): δ 12.78 (s, 1H, H-amide), 8.17 (dd, J = 8.0, 1.5 Hz, 1H, H-arom), 8.08 (s, 1H, H-arom), 7.90 – 7.74 (m, 4H, H-arom), 7.59 – 7.53 (m, 1H, H-arom), 7.53 – 7.48 (m, 1H, H-arom), 7.40 – 7.34 (m, 1H, H-arom). ^13^C NMR (125 MHz, DMSO-*d*_6_, ppm): δ 162.00, 155.40, 148.90, 148.16, 144.73, 135.25, 128.07, 127.84, 127.58, 126.48, 124.37, 123.18, 122.04, 112.31, 110.71. HRMS (ESI^+^) calculated for C_16_H_11_N_2_O_2_^+^ [M + H]^+^: 263.0815. Found: 263.0827.

#### 5.3.14 2-(3-Methylpyridin-2-yl)­quinazolin-4­(3*H*)-one

Obtained as a white solid. 44% yield. The compound is known in the literature.^40^

IR (ATR, v_máx_, cm^–1^): 3296 (νNH, amide), 1670 (νCO, amide), 1613 (δNH, amide). ^1^H NMR (500 MHz, DMSO-*d*_6_, ppm): δ 12,08 (s, 1H, H-amide), 8.64 – 8.49 (m, 1H, H-arom), 8.20 (dd, *J* = 8.0, 1.5 Hz, 1H, H-arom), 7.91 – 7.84 (m, 2H, H-arom), 7.76 (dd, *J* = 8.2, 1.1 Hz, 1H, H-arom), 7.59 (ddd, *J* = 8.2, 7.1, 1.2 Hz, 1H, H-arom), 7.53 (dd, *J* = 7.7, 4.7 Hz, 1H, H-arom), 2.67 (s, 3H, CH_3_). ^13^C NMR (125 MHz, DMSO-*d*_6_, ppm): δ 161.59, 152.22, 148.74, 148.63, 146.89, 140.69, 135.06, 134.32, 128.21, 127.71, 126.36, 125.83, 122.01, 20.37. HRMS (ESI^+^) calculated for C_14_H_12_N_3_O^+^ [M + H]^+^: 238.0975. Found: 238.0971.

#### 5.3.15 2-(3-Hydroxy-4-nitrophenyl)­quinazolin-4­(3*H*)-one

Obtained as a mustard yellow solid. 47% yield. The compound is known in the literature.^43^

IR (ATR, v_máx_, cm^–1^): 3650 (νOH, phenol), 1676 (νCO, amide), 1601 (δNH, amide). ^1^H NMR (500 MHz, DMSO-*d*_6_, ppm): δ 12.78 (s, 1H, H-amide), 11.41 (s, 1H, OH), 8.24 (d, J = 6.3 Hz, 1H, H-arom), 8.09 (d, J = 8.5 Hz, 1H, H-arom), 7.98 (d, J = 1.9 Hz, 1H, H-arom), 7.96 – 7.91 (m, 1H, H-arom), 7.83 (d, J = 7.2 Hz, 1H, H-arom), 7.78 (dd, J = 8.6, 1.9 Hz, 1H, H-arom), 7.64 (t, J = 8.1 Hz, 1H, H-arom). ^13^C NMR (125 MHz, DMSO-*d*_6_, ppm): δ 162.51, 152.06, 151.23, 148.80, 139.03, 138.90, 135.27, 128.18, 127.74, 126.40, 125.88, 121.73, 119.01, 118.86. HRMS (ESI^+^) calculated for C_14_H_10_N_3_O_4_^+^ [M + H]^+^: 284.0666. Found: 284.0661.

#### 5.3.16 2-(2-Aminopyridin-3-yl)­quinazolin-4­(3*H*)-one

Obtained as a white solid. 26% yield. The compound is novel in the scientific literature.

IR (ATR, v_máx_, cm^–1^): 3326 (νNH, amide), 1677 (νCO, amide), 1593 (δNH, amide). ^1^H NMR (500 MHz, DMSO-*d*_6_, ppm): δ 11.76 (s, 1H, H-amide), 8.35 (d, *J* = 7.8 Hz, 1H, H-arom), 8.29 (dd, *J* = 7.9, 1.6 Hz, 1H, H-arom), 8.05 (t, *J* = 7.7 Hz, 1H, H-arom), 8.00 – 7.94 (m, 1H, H-arom), 7.90 (d, *J* = 7.0 Hz, 1H, H-arom), 7.67 (ddd, *J* = 8.0, 7.1, 1.2 Hz, 1H, H-arom), 7.61 (d, *J* = 7.6 Hz, 1H, H-arom), 3.43 (s, 2H, NH_2_). ^13^C NMR (125 MHz, DMSO-*d*_6_, ppm): δ 161.19, 158.29, 150.32, 149.04, 148.16, 138.62, 135.21, 128.19, 127.69, 126.59, 122.46, 119.57. HRMS (ESI^+^) calculated for C_13_H_10_N_4_ONa^+^ [M + Na]^+^: 261.0752. Found: 261.0821.

#### 5.3.17 2-(2,3-Dichlorophenyl)­quinazolin-4­(3*H*)-one

Obtained as a white solid. 94% yield. The compound is known in the literature.^44^

IR (ATR, v_máx_, cm^–1^): 3165 (νNH, amide), 1671 (νCO, amide), 1604 (δNH, amide). ^1^H NMR (500 MHz, DMSO-*d*_6_, ppm): δ 12.78 (s, 1H, H-amide), 8.29 (dd, J = 8.0, 1.5 Hz, 1H, H-arom), 7.99 – 7.92 (m, 2H, H-arom), 7.82 (d, J = 8.1 Hz, 1H, H-arom), 7.76 (dd, J = 7.6, 1.5 Hz, 1H, H-arom), 7.71 – 7.67 (m, 1H, H-arom), 7.63 (t, J = 7.9 Hz, 1H, H-arom). ^13^C NMR (125 MHz, DMSO-*d*_6_, ppm): δ 161.87, 152.25, 148.90, 136.50, 135.14, 132.49, 132.40, 130.35, 129.95, 129.01, 127.95, 127.73, 126.36, 121.82. HRMS (ESI^+^) calculated for C_14_H_9_Cl_2_N_2_O^+^ [M + H]^+^: 291.0086. Found: 291.0084.

#### 5.3.18 2-(3,5-Difluorophenyl)­quinazolin-4­(3*H*)-one

Obtained as a white solid. 90% yield. The compound is known in the literature.^46^

IR (ATR, v_máx_, cm^–1^): 3177 (νNH, amide), 1680 (νCO, amide), 1594 (δNH, amide). ^1^H NMR (500 MHz, DMSO-*d*_6_, ppm): δ 12.76 (s, 1H, H-amide), 8.28 (d, J = 7.9 Hz, 1H, H-arom), 8.04 (d, J = 6.9 Hz, 2H, H-arom), 7.97 (d, J = 7.4 Hz, 1H, H-arom), 7.89 (d, J = 8.0 Hz, 1H, H-arom), 7.73 – 7.58 (m, 2H, H-arom). ^13^C NMR (125 MHz, DMSO-*d*_6_, ppm): δ 162,84 (J = 247,5 Hz, 12,5 Hz), 162,52, 150.44, 148.65, 136.61, 135.27, 128.20, 127.73, 126.39, 121.72, 111,59 (J = 27,5 Hz), 107.30. HRMS (ESI^+^) calculated for C_14_H_9_F_2_N_2_O^+^ [M + H]^+^: 259.0677. Found: 259.0675.

#### 5.3.19 2-(6-(Trifluoromethyl)­pyridin-3-yl)­quinazolin-4­(3*H*)-one

Obtained as a white solid. 36% yield. The compound is novel in the scientific literature.

IR (ATR, v_máx_, cm^–1^): 3190 (νNH, amide), 1663 (νCO, amide), 1607 (δNH, amide). ^1^H NMR (500 MHz, DMSO-*d*_6_, ppm): δ 12.91 (s, 1H, H-amide), 9.45 (s, 1H, H-arom), 8.77 (dd, J = 8.3, 2.2 Hz, 1H, H-arom), 8.19 (dd, J = 8.0, 1.5 Hz, 1H, H-arom), 8.12 (d, J = 9.1 Hz, 1H, H-arom), 7.93 – 7.85 (m, 1H, H-arom), 7.84 – 7.78 (m, 1H, H-arom), 7.63 – 7.55 (m, 1H, H-arom). ^13^C NMR (125 MHz, DMSO-*d*_6_, ppm): δ 162.47, 150.08, 149.77, 148,34 (J = 32,9 Hz), 138.32, 135.29, 132.42, 128.21, 127.91, 126.40, 121,92 (J = 272,5 Hz), 121.80, 121.11. HRMS (ESI^+^) calculated for C_14_H_9_F_3_N_3_O^+^ [M + H]^+^: 292.0692. Found: 292.0690.

#### 5.3.20 2-(5-Chloro-1H-indol-3-yl)­quinazolin-4­(3*H*)-one

Obtained as a banana yellow solid. 58% yield. The compound is novel in the scientific literature.

IR (ATR, v_máx_, cm^–1^): 3426 (νNH, amine), 3171 (νNH, amide), 1673 (νCO, amide), 1595 (δNH, amide). ^1^H NMR (500 MHz, DMSO-*d*_6_, ppm): δ 12.28 (s, 1H, H-amide), 12.11 (s, 1H, NH-indole), 8.76 (d, J = 2.2 Hz, 1H, H-arom), 8.67 (d, J = 3.1 Hz, 1H, H-arom), 8.17 (dd, J = 7.8, 1.5 Hz, 1H, H-arom), 7.89 – 7.83 (m, 1H, H-arom), 7.81 (d, J = 7.6 Hz, 1H, H-arom), 7.58 (d, J = 8.6 Hz, 1H, H-arom), 7.53 – 7.47 (m, 1H, H-arom), 7.32 (dd, J = 8.6, 2.2 Hz, 1H, H-arom). ^13^C NMR (125 MHz, DMSO-*d*_6_, ppm): δ 162.54, 150.35, 149.97, 135.82, 134.96, 131.02, 127.51, 127.11, 126.27, 126.12, 125.87, 123.17, 121.91, 120.95, 114.15, 108.78. HRMS (ESI^+^) calculated for C_16_H_11_ClN_3_O^+^ [M + H]^+^: 296.0585. Found: 296.0582.

## Supplementary Material



## Data Availability

The authors confirm that the data supporting the findings of this study are available within the article and its Supporting Information. Further inquiries can be directed to the corresponding author.

## Notes

The compounds were characterized by Fourier Transform Infrared Spectroscopy (FTIR), Nuclear Magnetic Resonance (^1^H and ^13^C NMR), and High-Resolution Mass Spectrometry (HRMS), and their corresponding spectra were recorded (PDF)
