# Supplementary material for: Synthesis and Evaluation of 2‑Substituted Quinazolin-4(3H)‑ones as Potential Antileukemic Agents
Source: ACS Omega. 2025 Aug 1;10(31):34882–94. doi: 10.1021/acsomega.5c04106 (PMC12355269; doi:10.1021/acsomega.5c04106)
Supplement: Supplementary file 1 [file ao5c04106_si_001.pdf]

Supporting Information

Synthesis and evaluation of 2-substituted quinazolin-4(3*H*)-ones as potential antileukemic agents

Giorgio Antoniolli*, Keli Lima, Gilberto Carlos Franchi, Carmen Silvia Passos Lima, João Agostinho Machado-Neto, Fernando Coelho

Giorgio Antoniolli – Institute of Chemistry, University of Campinas, Campinas, SP, Brazil

Keli Lima – Faculty of Medicine, University of São Paulo, São Paulo, SP, Brazil

Gilberto Carlos Franchi – School of Medical Sciences, University of Campinas, Campinas, SP, Brazil

Carmen Silvia Passos Lima – School of Medical Sciences, University of Campinas, Campinas, SP, Brazil

João Agostinho Machado-Neto – Institute of Biomedical Sciences, University of São Paulo, São Paulo, SP, Brazil

Fernando Coelho – Institute of Chemistry, University of Campinas, Campinas, SP, Brazil

Corresponding author*:

Giorgio Antoniolli

Email: g191000@dac.unicamp.br, and antoniolli.giorgio@gmail.com

The compounds were characterized by Fourier Transform Infrared Spectroscopy (FTIR), Nuclear Magnetic Resonance (^{1}H and ^{13}C NMR), and High-Resolution Mass Spectrometry (HRMS), and their corresponding spectra were recorded.

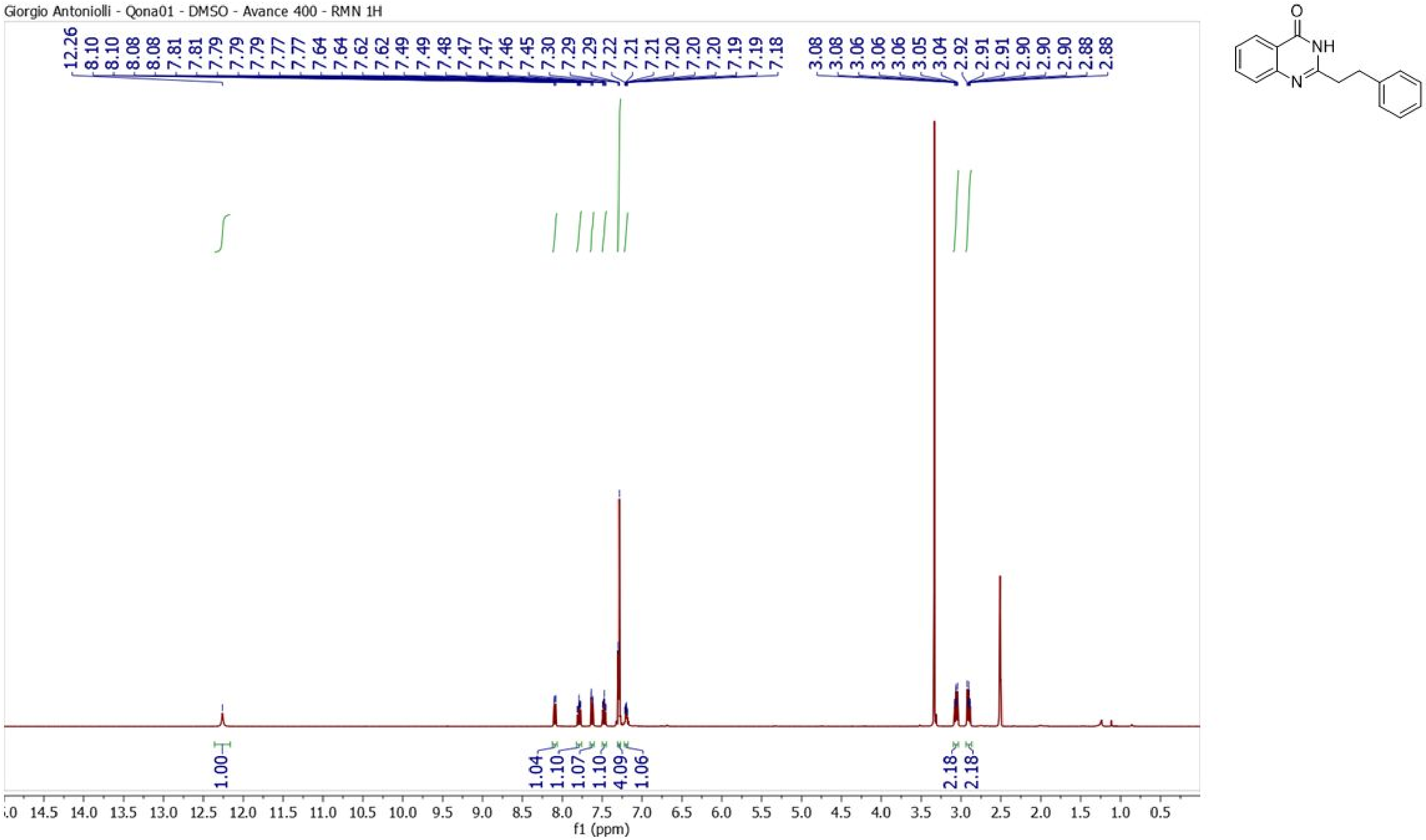

Figure 1S. ^{1}H NMR (d$_6$-DMSO, 400 MHz) of 2-phenethylquinazolin-4(3*H*)-one, **1**.

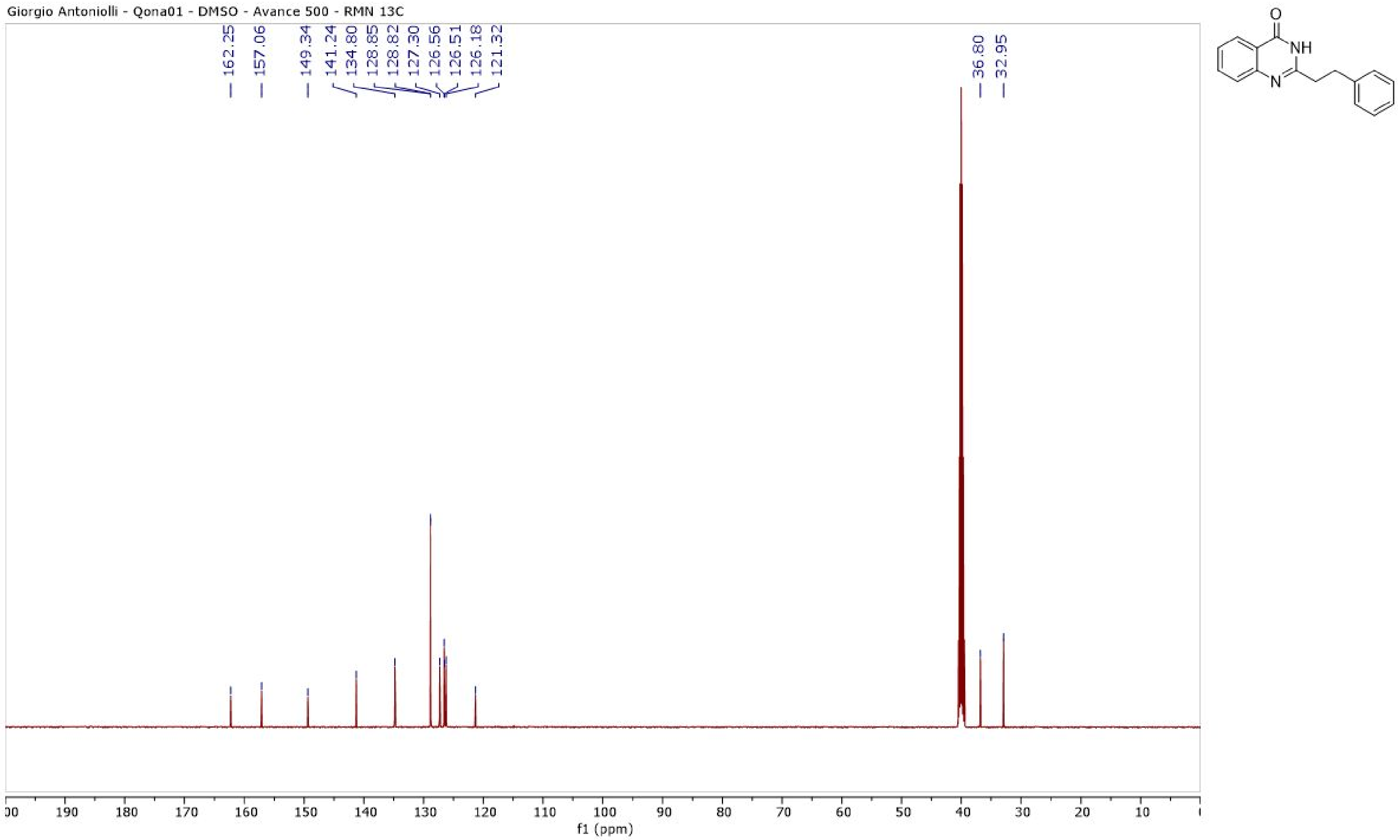

Figure 2S. ^{13}C NMR (d_6-DMSO, 125 MHz) of 2-phenethylquinazolin-4(3*H*)-one, **1**.

Sample ID: Qona01

Sample Scans: 64

Background Scans: 64

Resolution: 4 cm-1

System Status: Good

File Location: C:\Program Files\Agilent\MicroLab PC\Results\Qona01_2023-08-17T14-33-49.a2r

Method Name: PADRAO ATR

User: Admin

Date/Time: 17-Aug-23 2:31:08PM

Range: 4,000.00 - 650.00

Apodization: Happ-Genzel

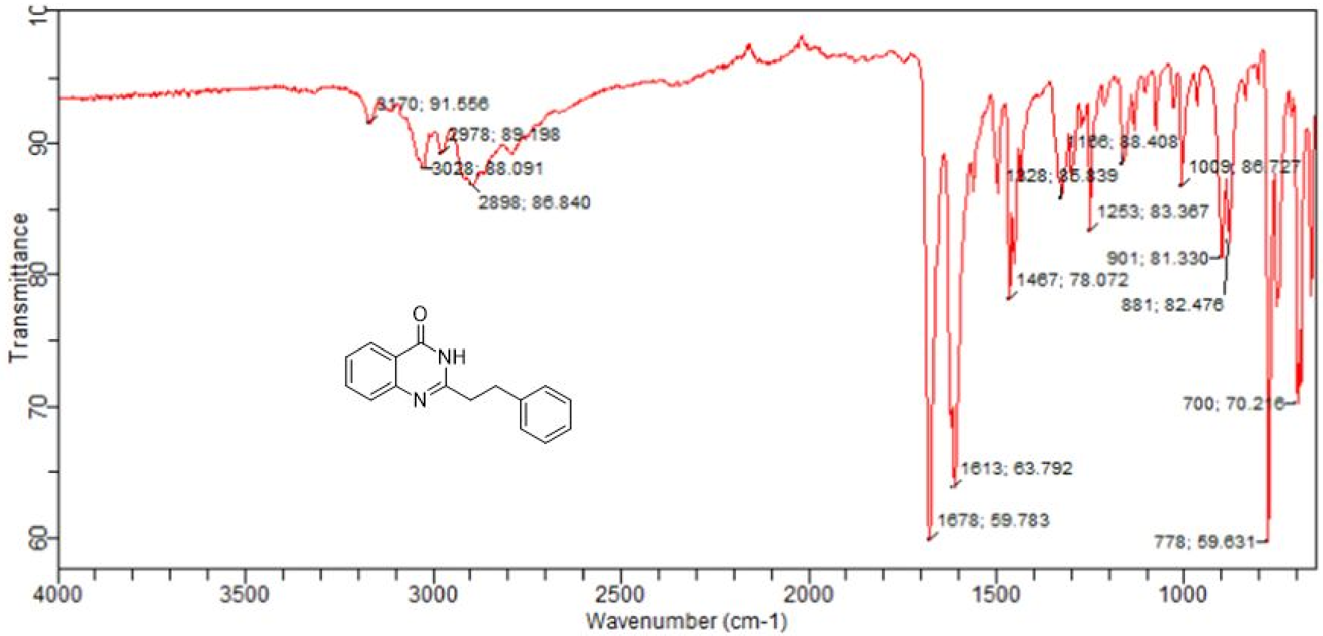

Figure 3S. IR (ATR, ν_{max}, cm^{-1}) of 2-phenethylquinazolin-4(3*H*)-one, **1**.

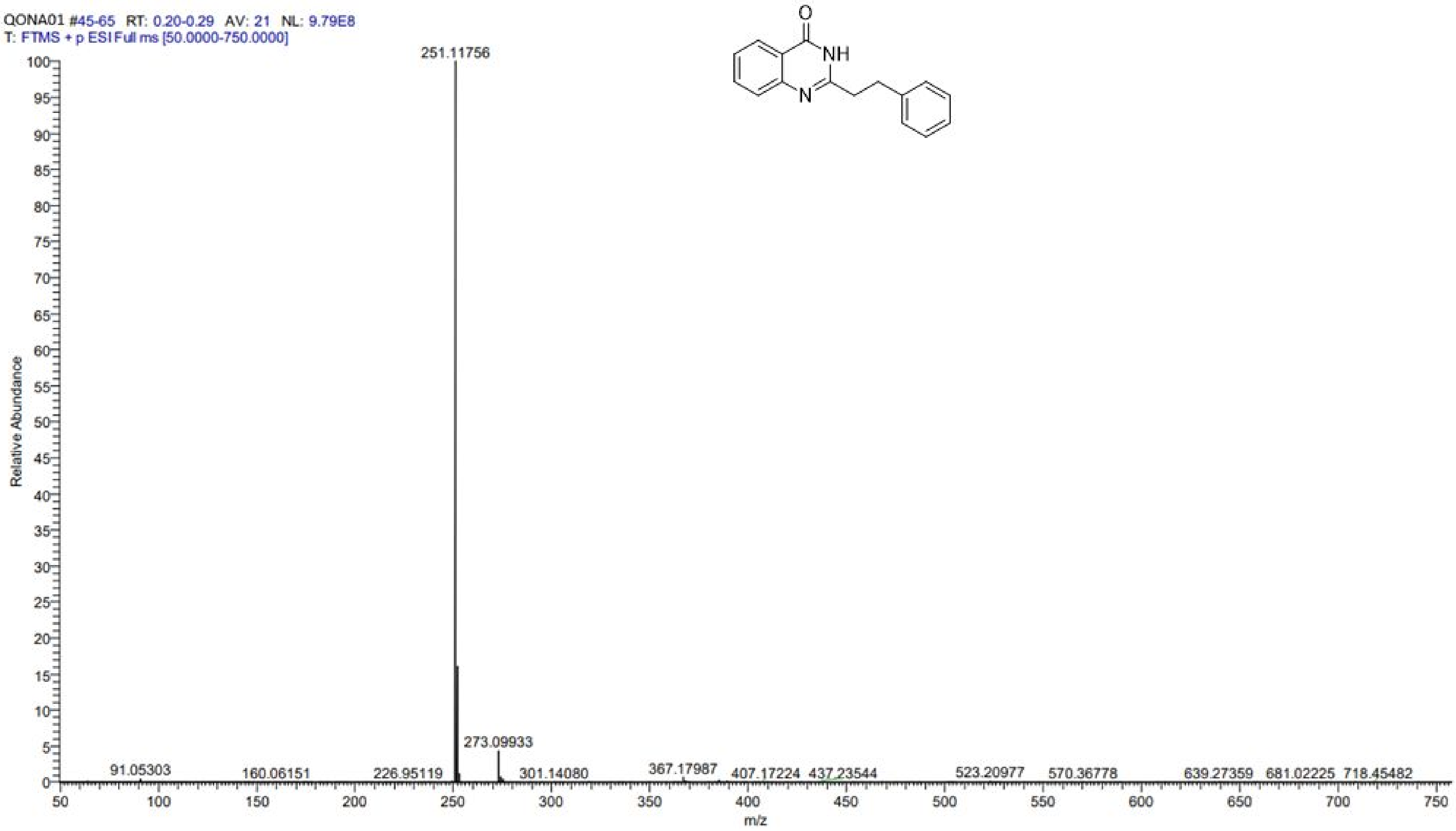

Figure 4S. MS of 2-phenethylquinazolin-4(3*H*)-one, **1**.

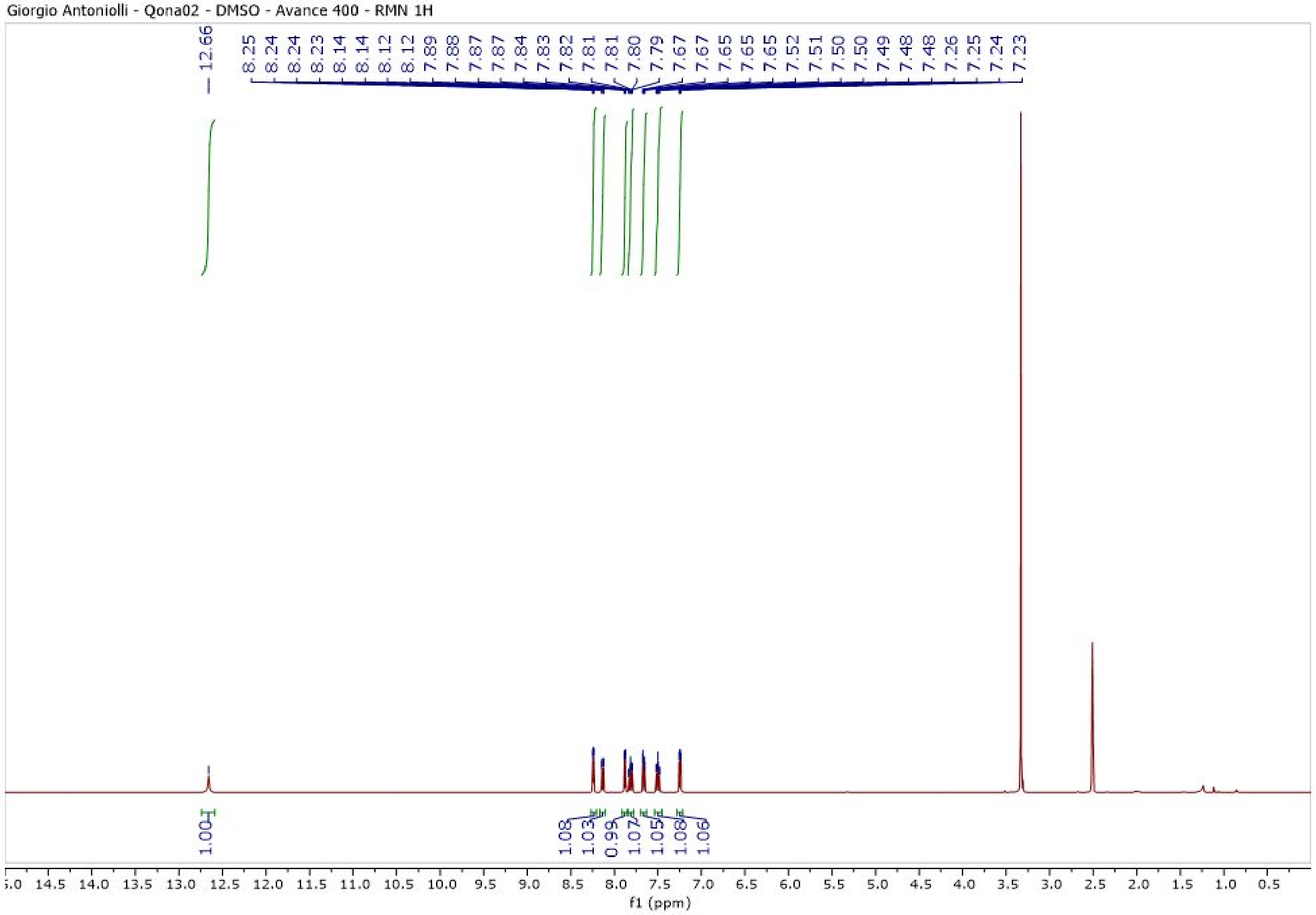

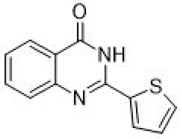

Figure 5S. ^{1}H NMR (d_6-DMSO, 400 MHz) of 2-(thiophen-2-yl)quinazolin-4(3*H*)-one, **2**.

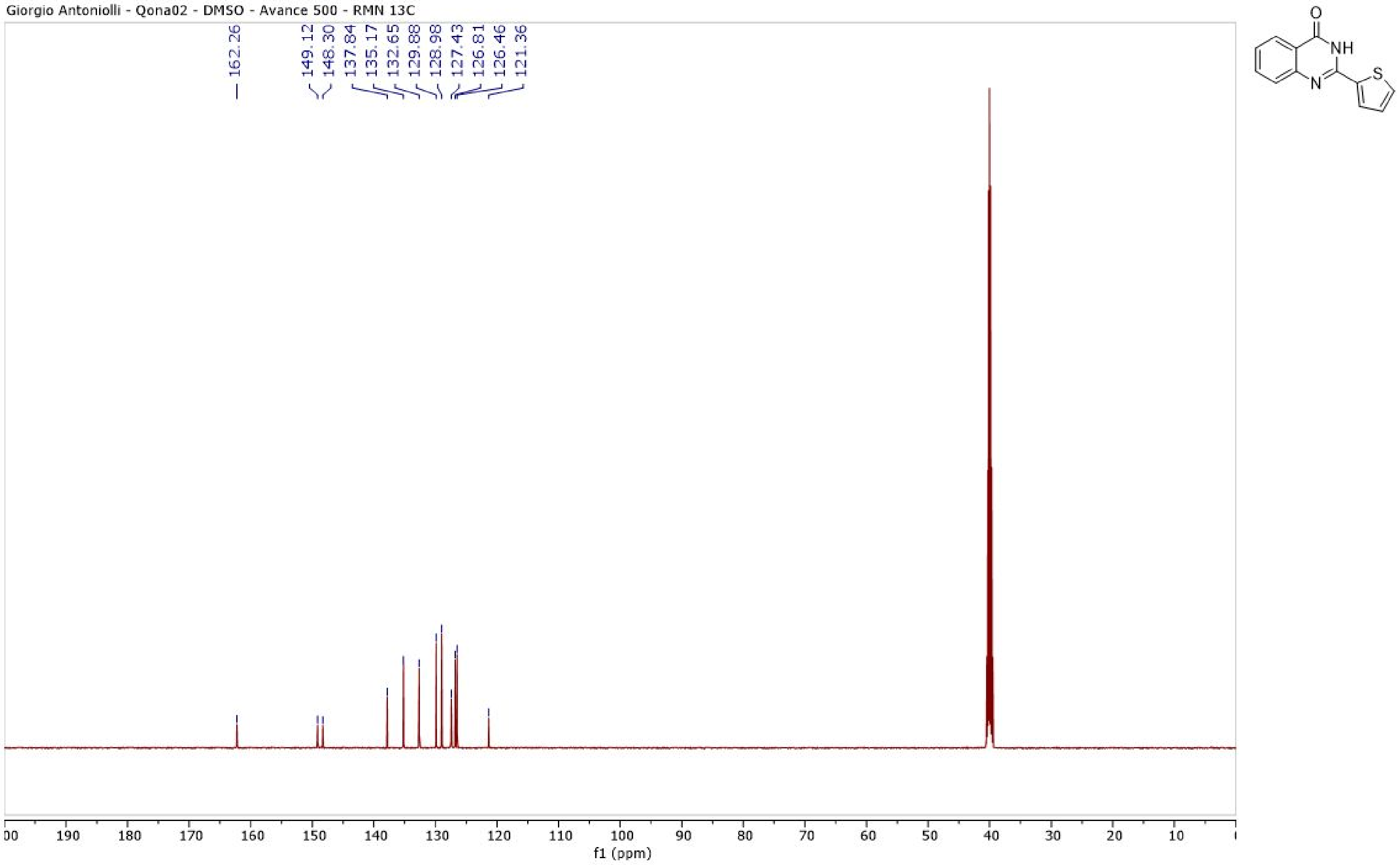

Figure 6S. ^{13}C NMR (d_6-DMSO, 125 MHz) of 2-(thiophen-2-yl)quinazolin-4(3*H*)-one, **2**.

Sample ID:	Qona02	Method Name:	PADRAO ATR
Sample Scans:	64	User:	Admin
Background Scans:	64	Date/Time:	17-Aug-23 2:36:23PM
Resolution:	4 cm-1	Range:	4,000.00 - 650.00
System Status:	Good	Apodization:	Happ-Genzel
File Location:	C:\Program Files\Agilent\MicroLab PC\Results\Qona02_2023-08-17T14-38-11.a2r		

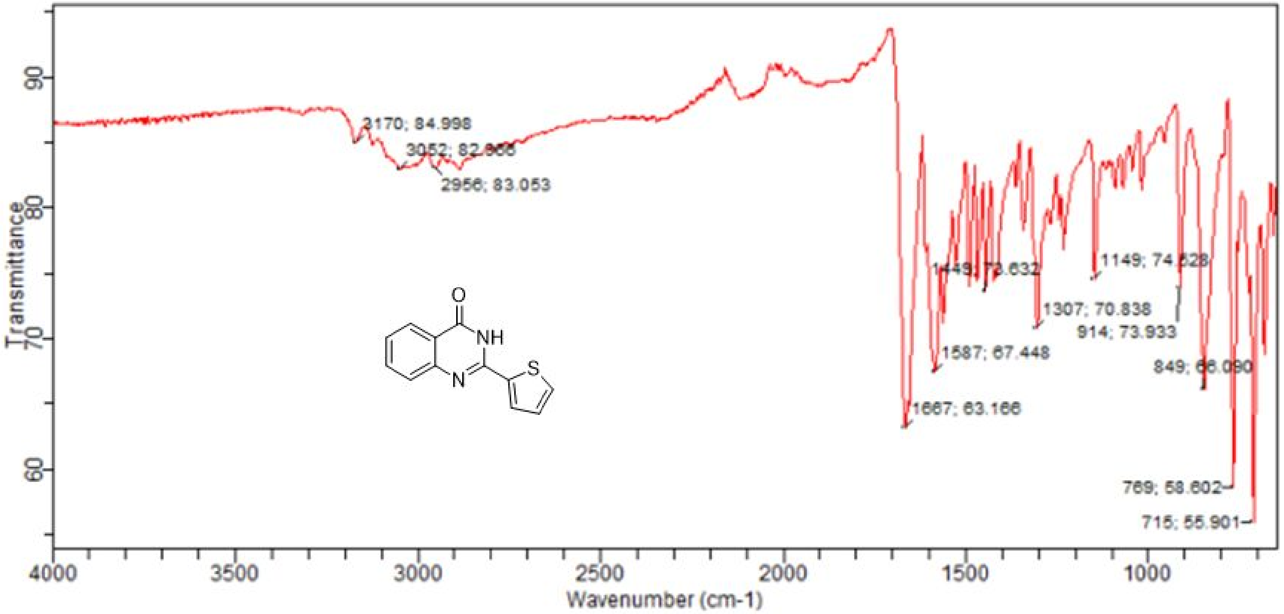

Figure 7S. IR (ATR, ν_{max}, cm^{-1}) of 2-(thiophen-2-yl)quinazolin-4(3*H*)-one, **2**.

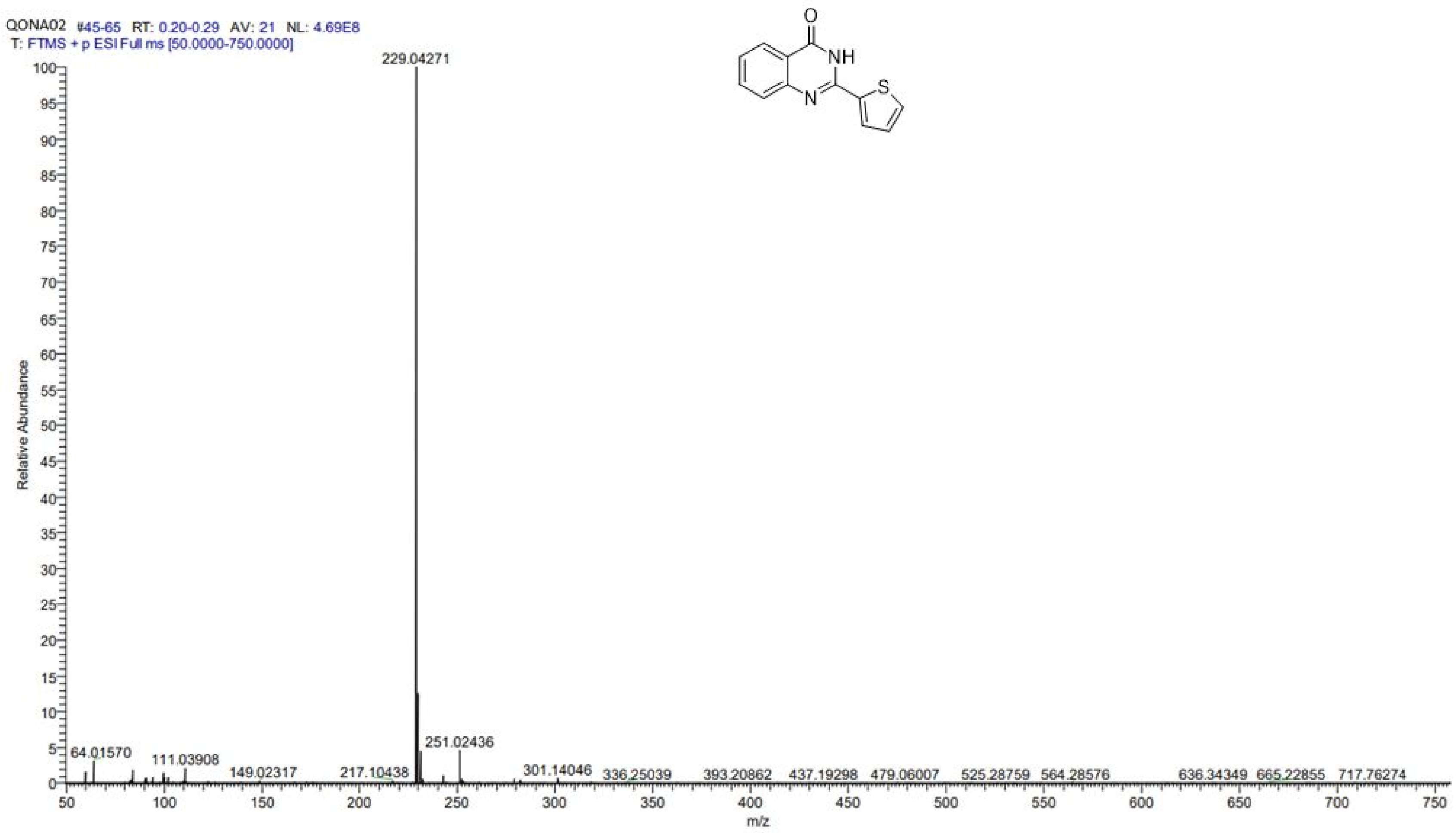

Figure 8S. MS of 2-(thiophen-2-yl)quinazolin-4(3*H*)-one, **2**.

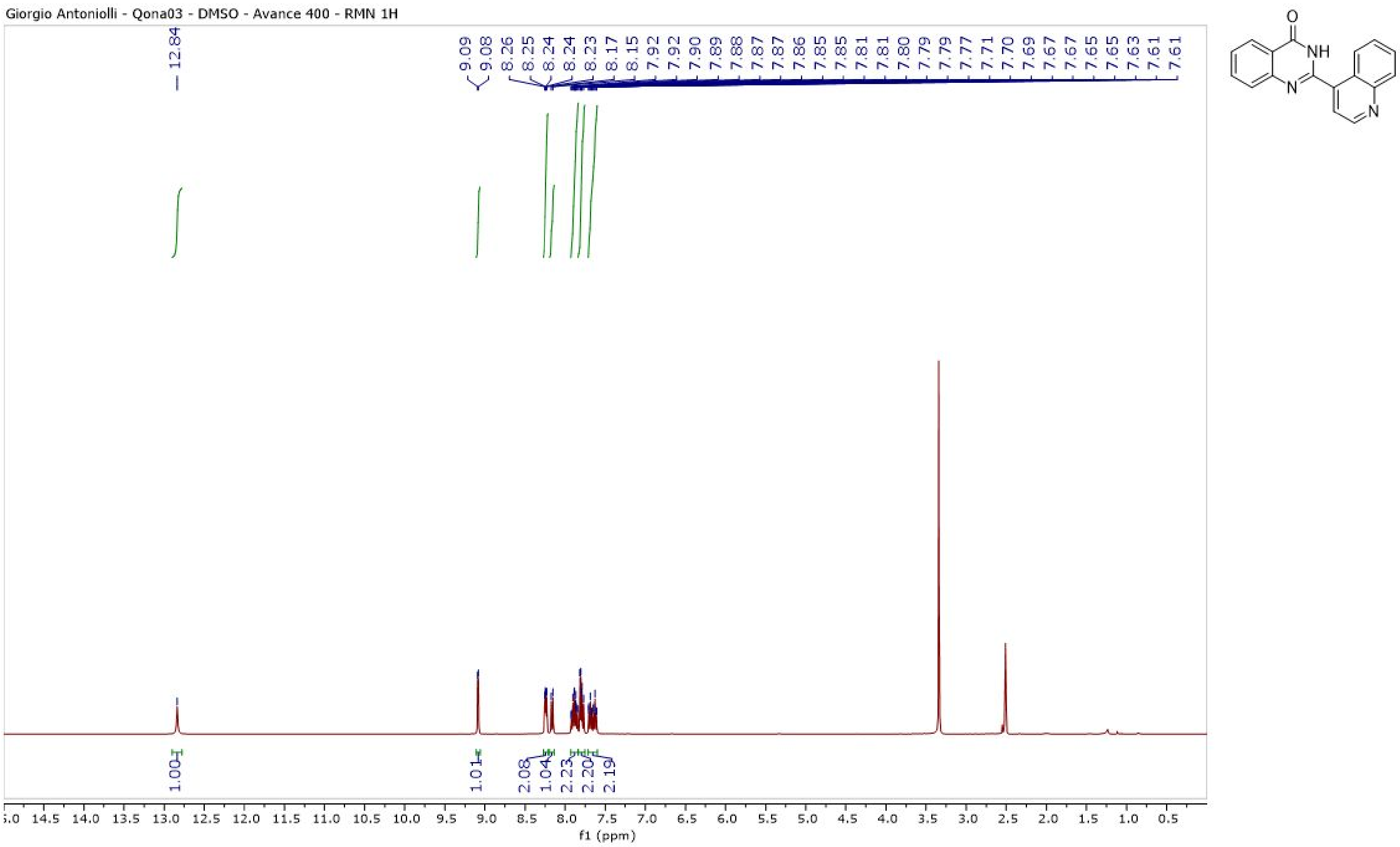

Figure 9S. ^{1}H NMR (d_6-DMSO, 400 MHz) of 2-(quinolin-4-yl)quinazolin-4(3*H*)-one, **3**.

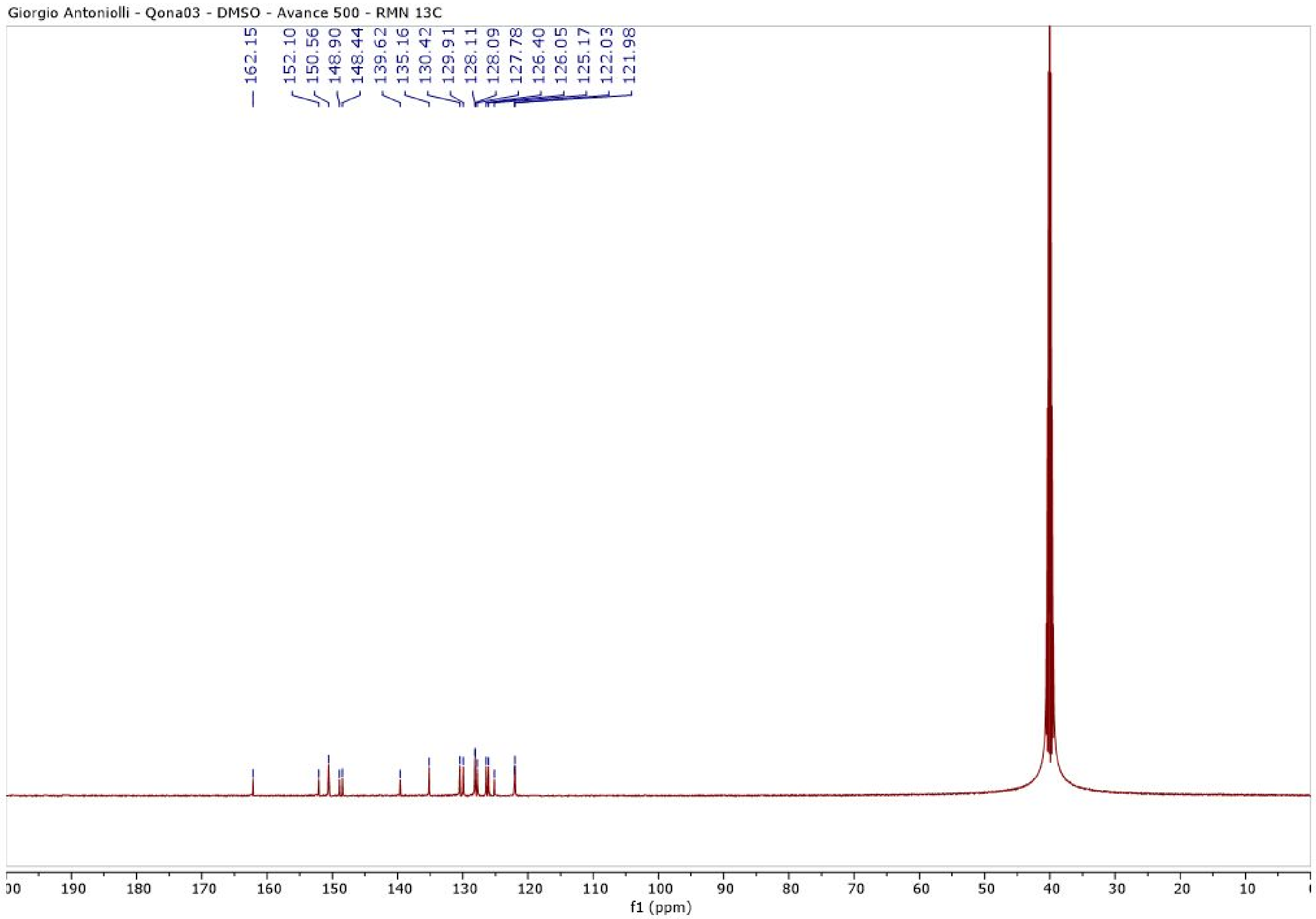

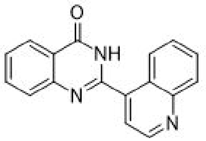

Figure 10S. ^{13}C NMR (d_6-DMSO, 125 MHz) of 2-(quinolin-4-yl)quinazolin-4(3*H*)-one, **3**.

Sample ID:	Qona03	Method Name:	PADRAO ATR
Sample Scans:	64	User:	Admin
Background Scans:	64	Date/Time:	17-Aug-23 2:40:12PM
Resolution:	4 cm-1	Range:	4,000.00 - 650.00
System Status:	Good	Apodization:	Happ-Genzel

File Location: C:\Program Files\Agilent\MicroLab PC\Results\Qona03_2023-08-17T14-41-53.a2r

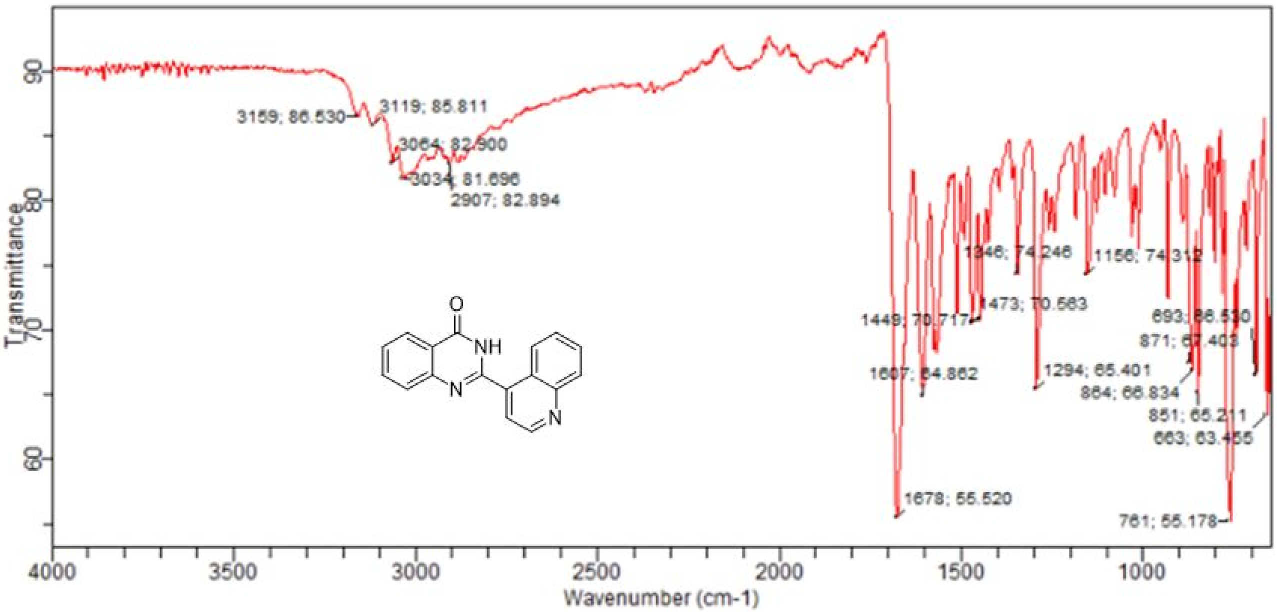

Figure 11S. IR (ATR, ν_{max}, cm^{-1}) of 2-(quinolin-4-yl)quinazolin-4(3*H*)-one, **3**.

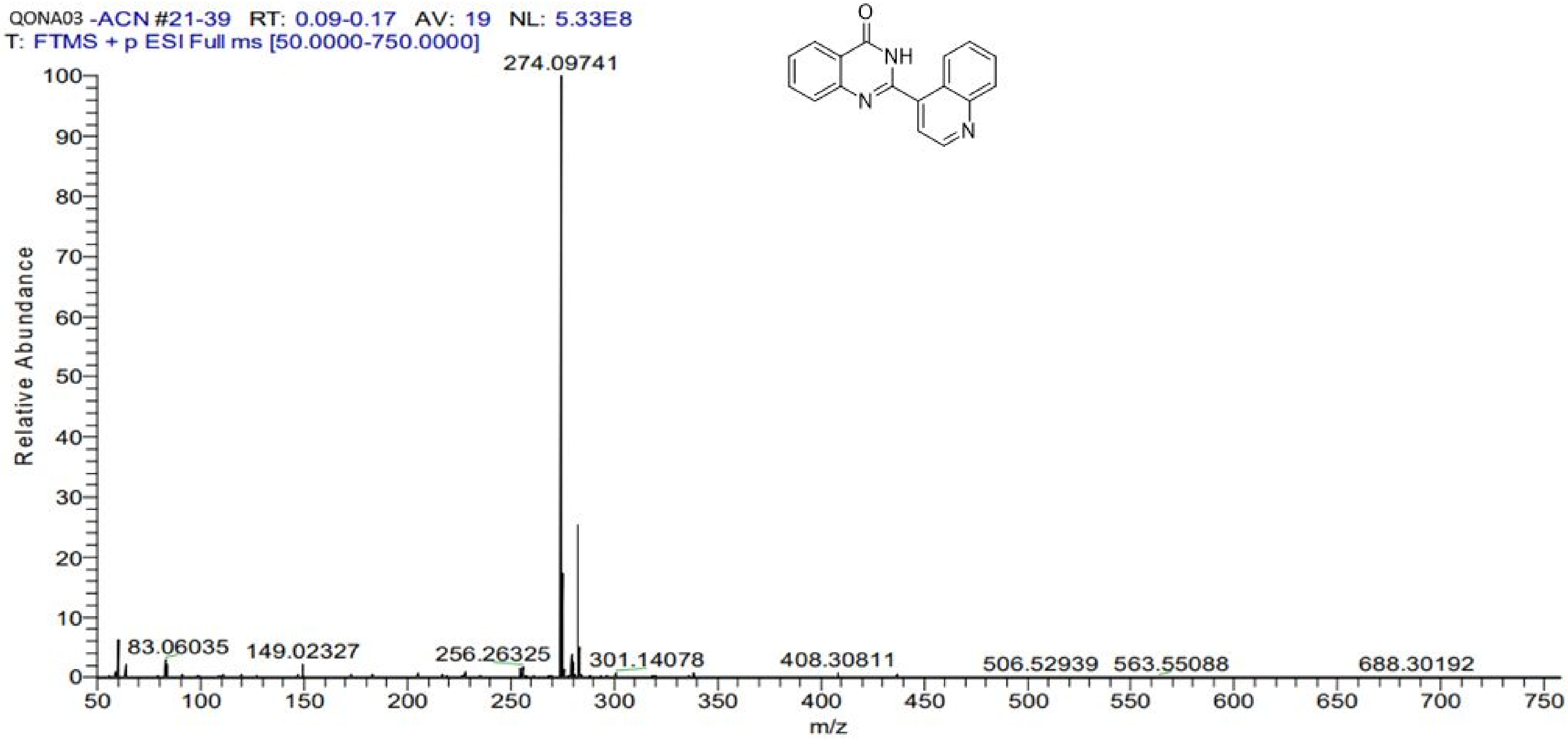

Figure 12S. MS of 2-(quinolin-4-yl)quinazolin-4(3*H*)-one, **3**.

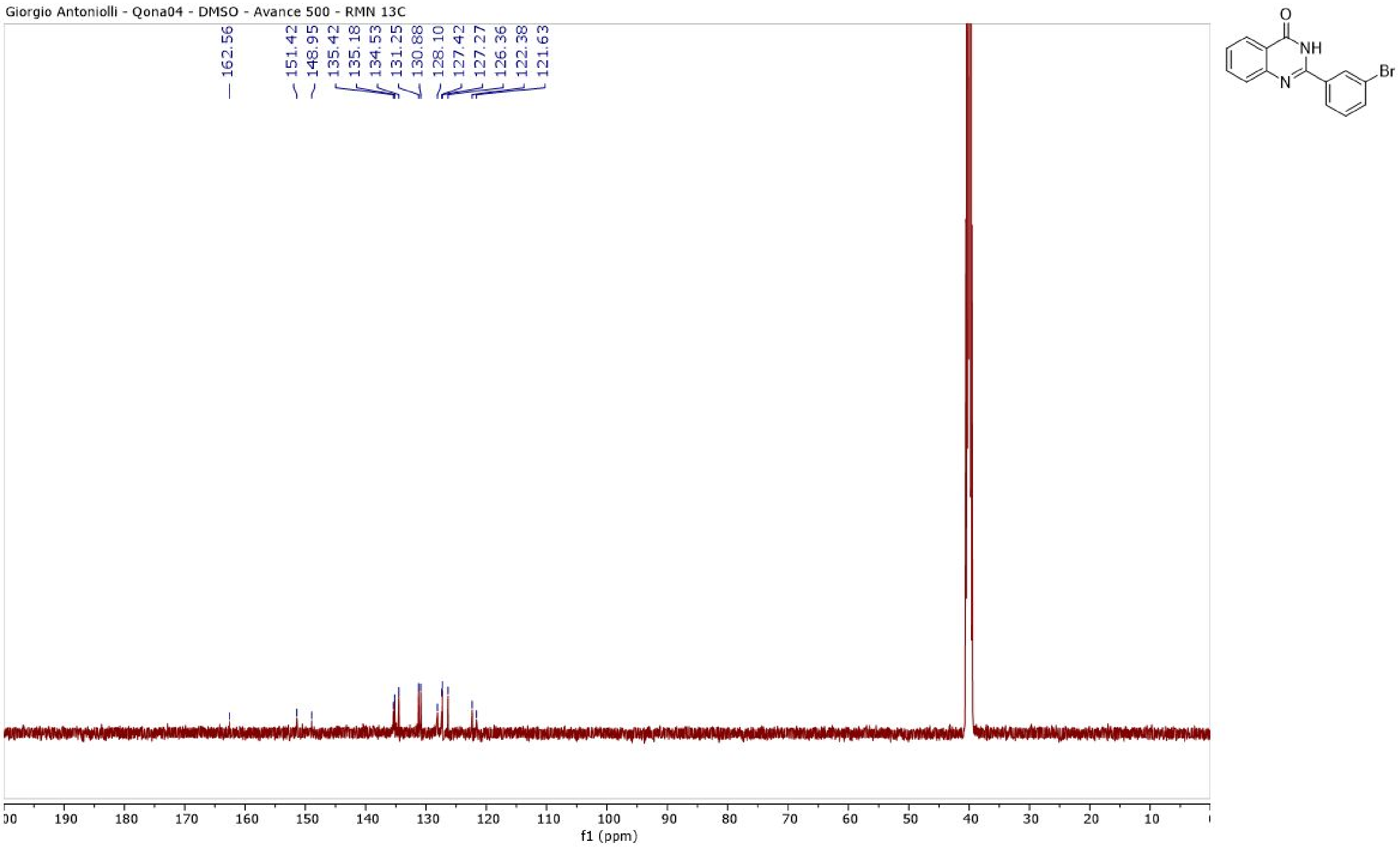

Figure 13S. ^{1}H NMR (d_6-DMSO, 400 MHz) of 2-(3-bromophenyl)quinazolin-4(3*H*)-one, **4**.

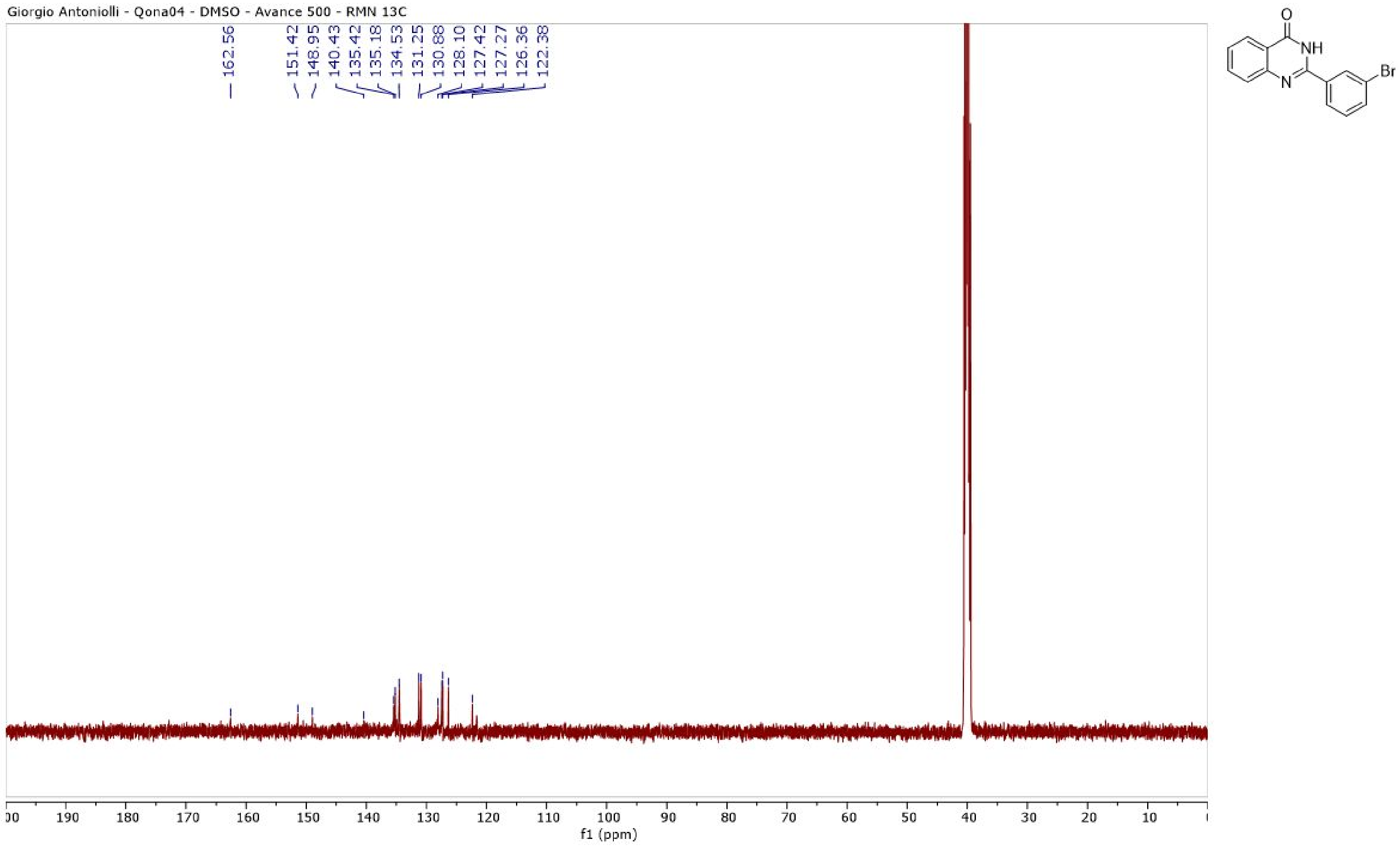

Figure 14S. ^{13}C NMR (d_6-DMSO, 125 MHz) of 2-(3-bromophenyl)quinazolin-4(3*H*)-one, **4**.

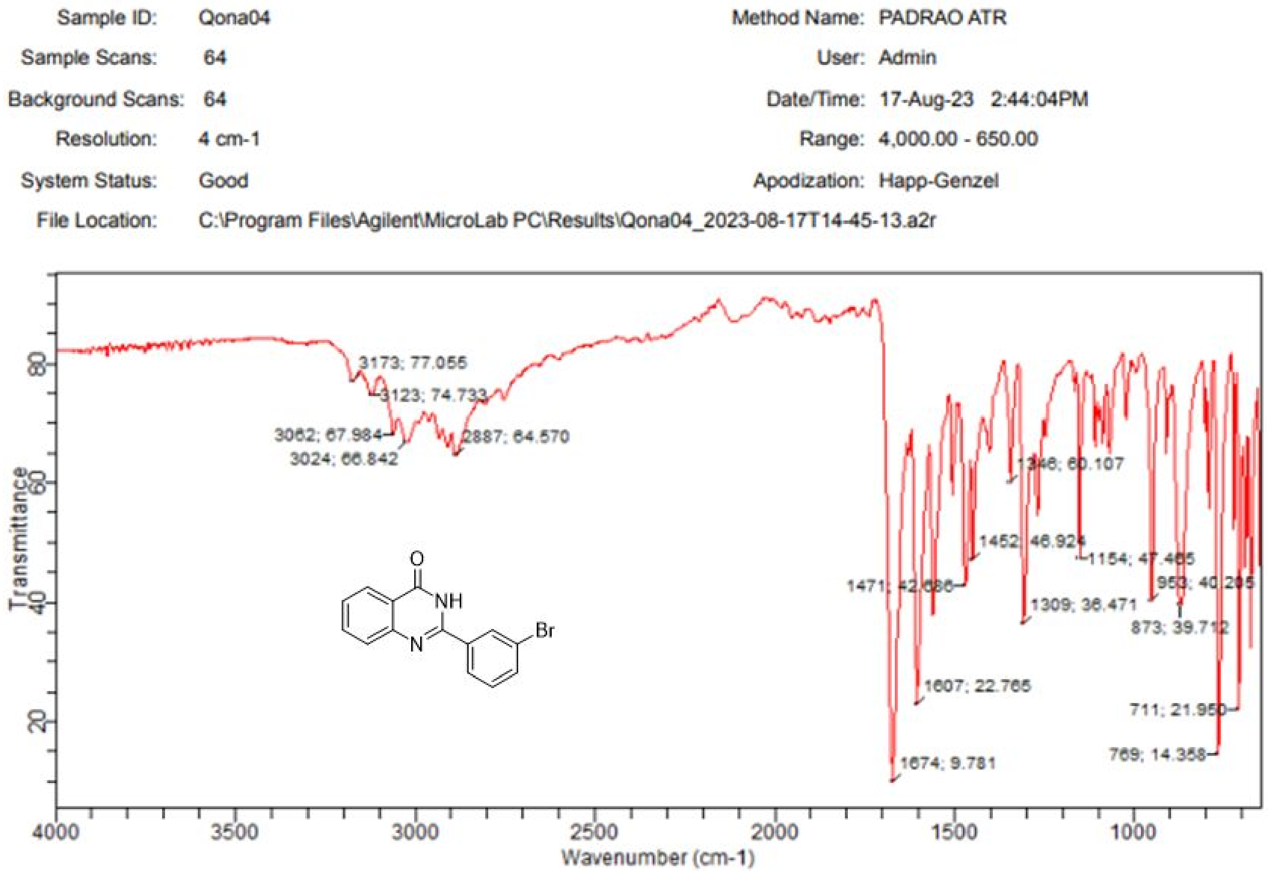

Figure 15S. IR (ATR, ν_{max}, cm^{-1}) of 2-(3-bromophenyl)quinazolin-4(3*H*)-one, **4**.

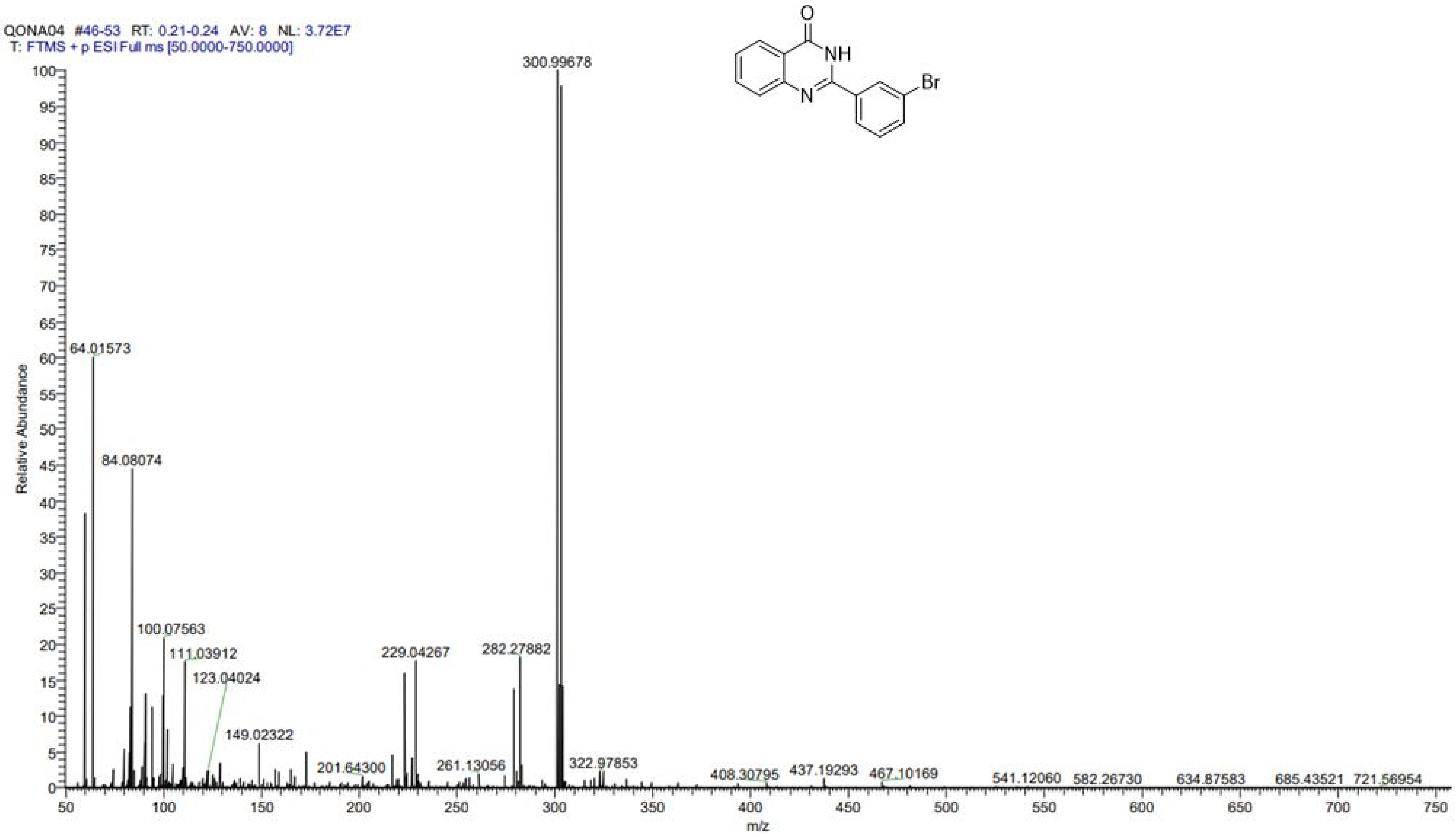

Figure 16S. MS of 2-(3-bromophenyl)quinazolin-4(3*H*)-one, **4**.

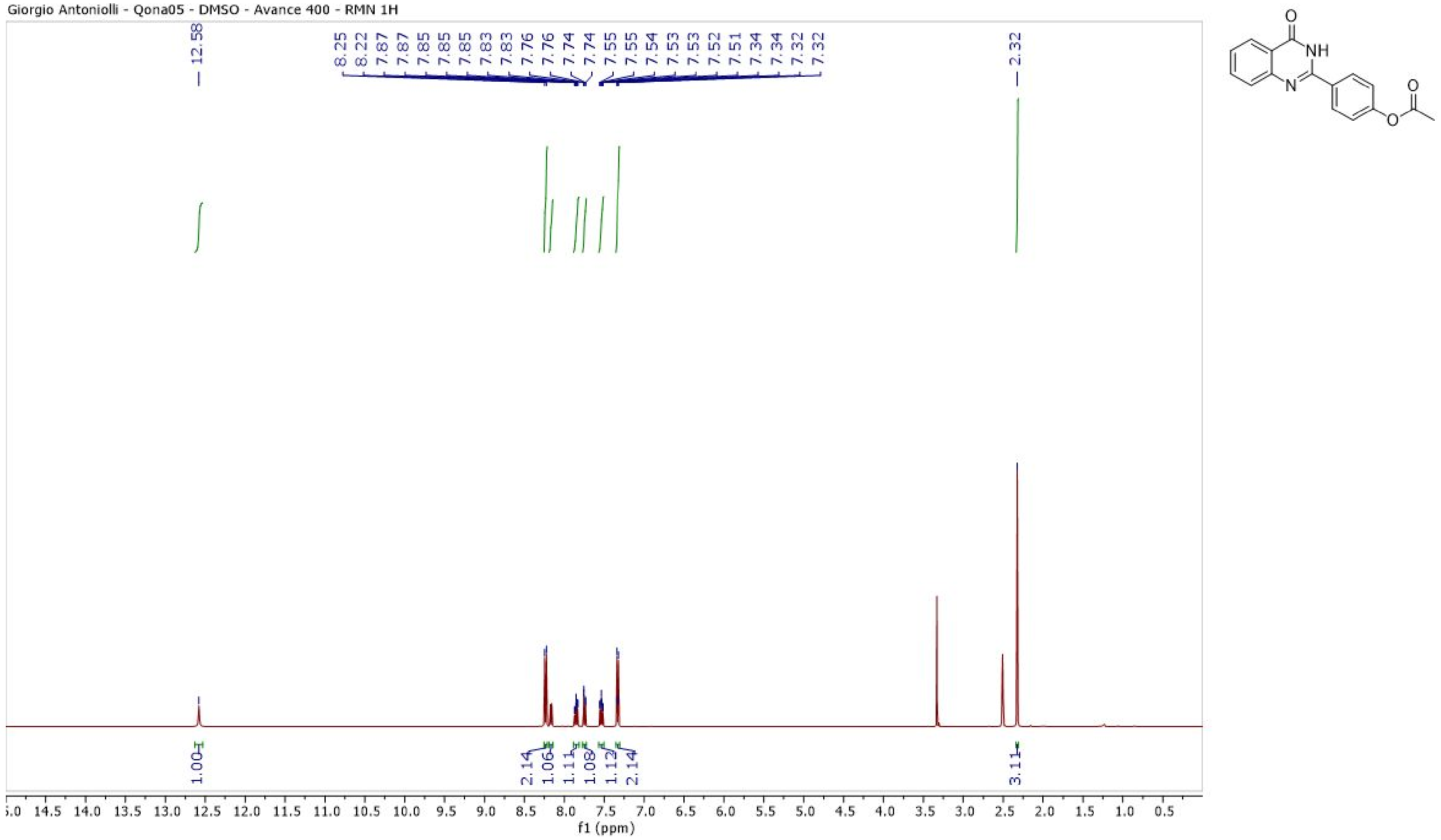

Figure 17S. ^{1}H NMR (d_6-DMSO, 400 MHz) of 4-(4-oxo-3,4-dihydroquinazolin-2-yl)phenyl acetate, **5**.

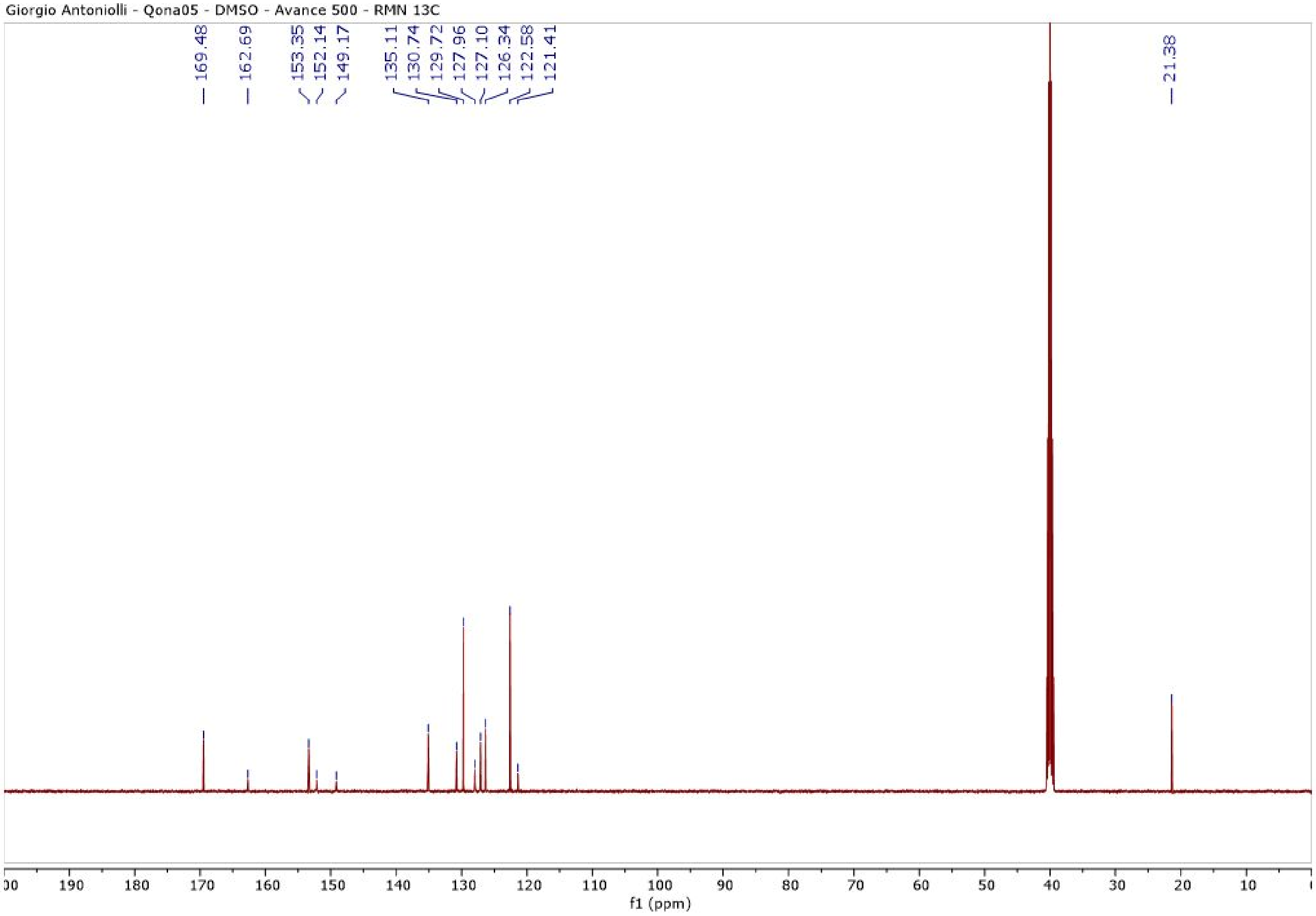

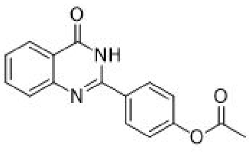

Figure 18S. ^{13}C NMR (d_6-DMSO, 125 MHz) of 4-(4-oxo-3,4-dihydroquinazolin-2-yl)phenyl acetate, **5**.

Sample ID:	Qona05	Method Name:	PADRAO ATR
Sample Scans:	64	User:	Admin
Background Scans:	64	Date/Time:	17-Aug-23 2:47:12PM
Resolution:	4 cm-1	Range:	4,000.00 - 650.00
System Status:	Good	Apodization:	Happ-Genzel
File Location:	C:\Program Files\Agilent\MicroLab PC\Results\Qona05_2023-08-17T14-48-34.a2r		

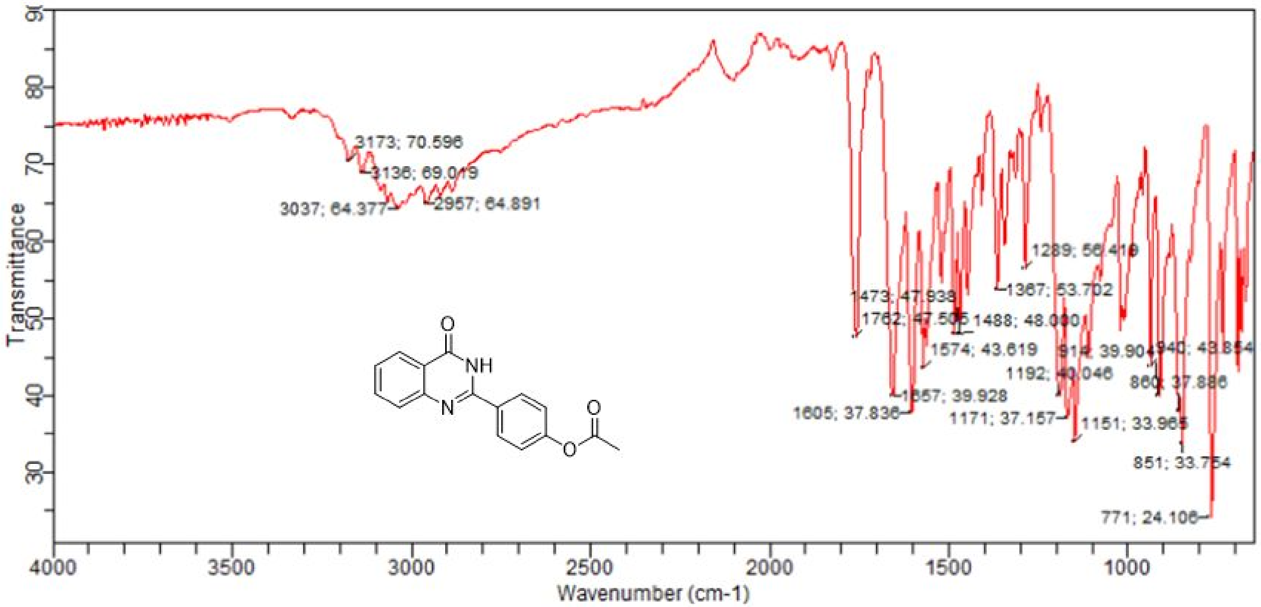

Figure 19S. IR (ATR, ν_{max}, cm^{-1}) of 4-(4-oxo-3,4-dihydroquinazolin-2-yl)phenyl acetate, **5**.

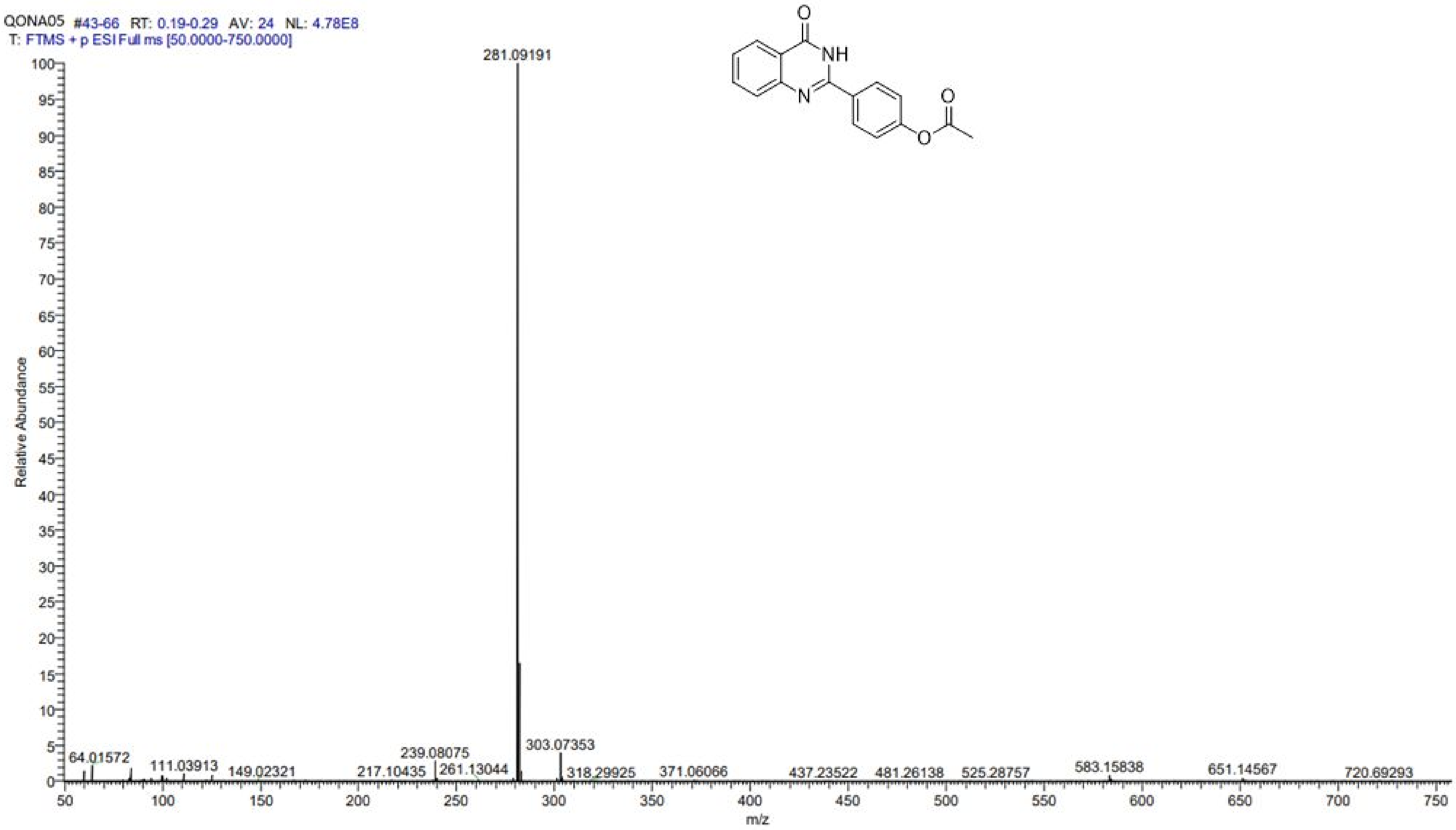

Figure 20S. MS of 4-(4-oxo-3,4-dihydroquinazolin-2-yl)phenyl acetate, **5**.

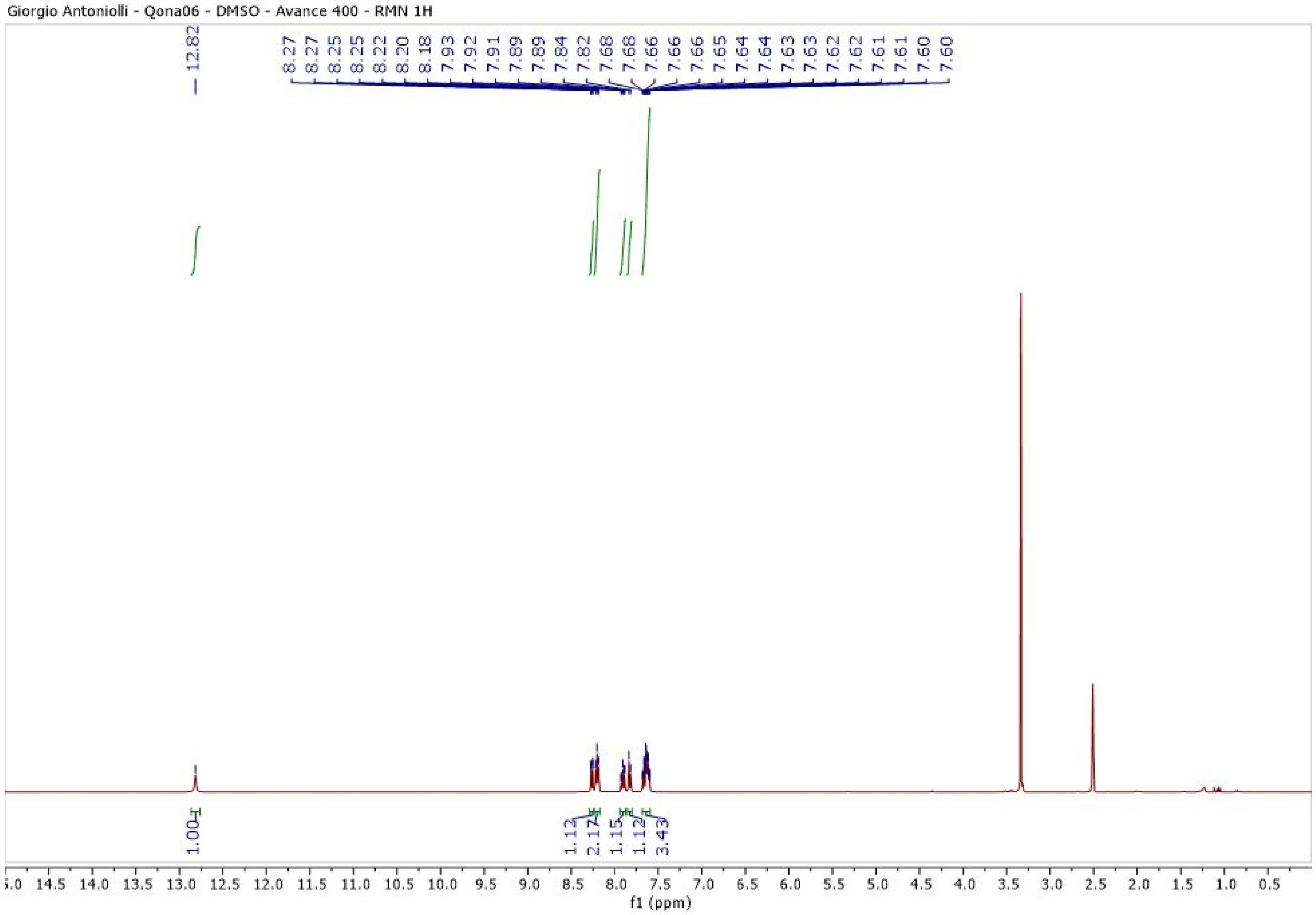

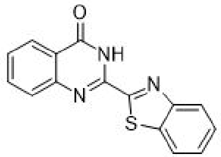

Figure 21S. ^{1}H NMR (d_6-DMSO, 400 MHz) of 2-(benzo[*d*]thiazol-2-yl)quinazolin-4(3*H*)-one, **6**.

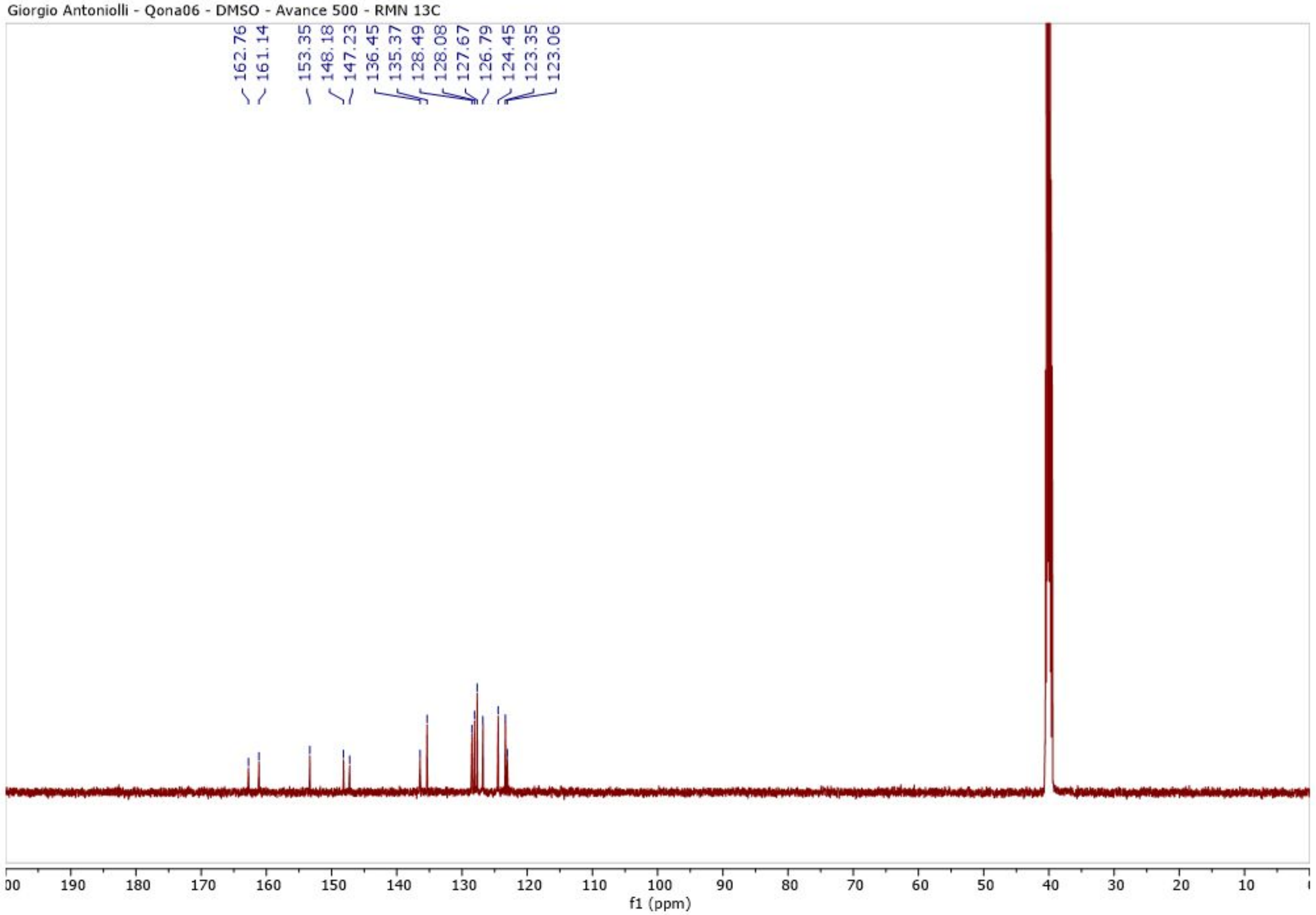

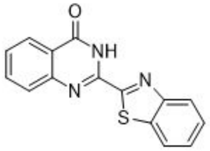

Figure 22S. ^{13}C NMR (d_6-DMSO, 125 MHz) of 2-(benzo[*d*]thiazol-2-yl)quinazolin-4(3*H*)-one, **6**.

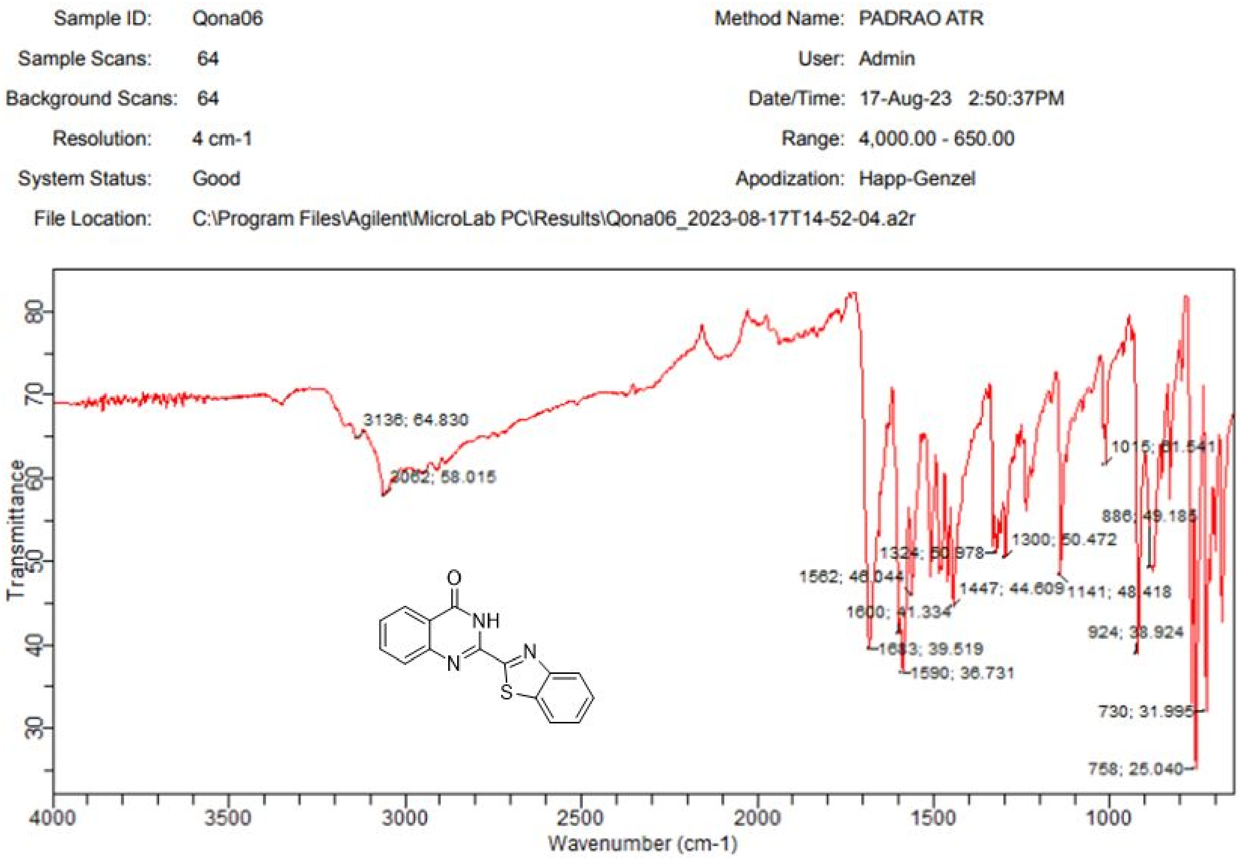

Figure 23S. IR (ATR, ν_{max}, cm^{-1}) of 2-(benzo[*d*]thiazol-2-yl)quinazolin-4(3*H*)-one, **6**.

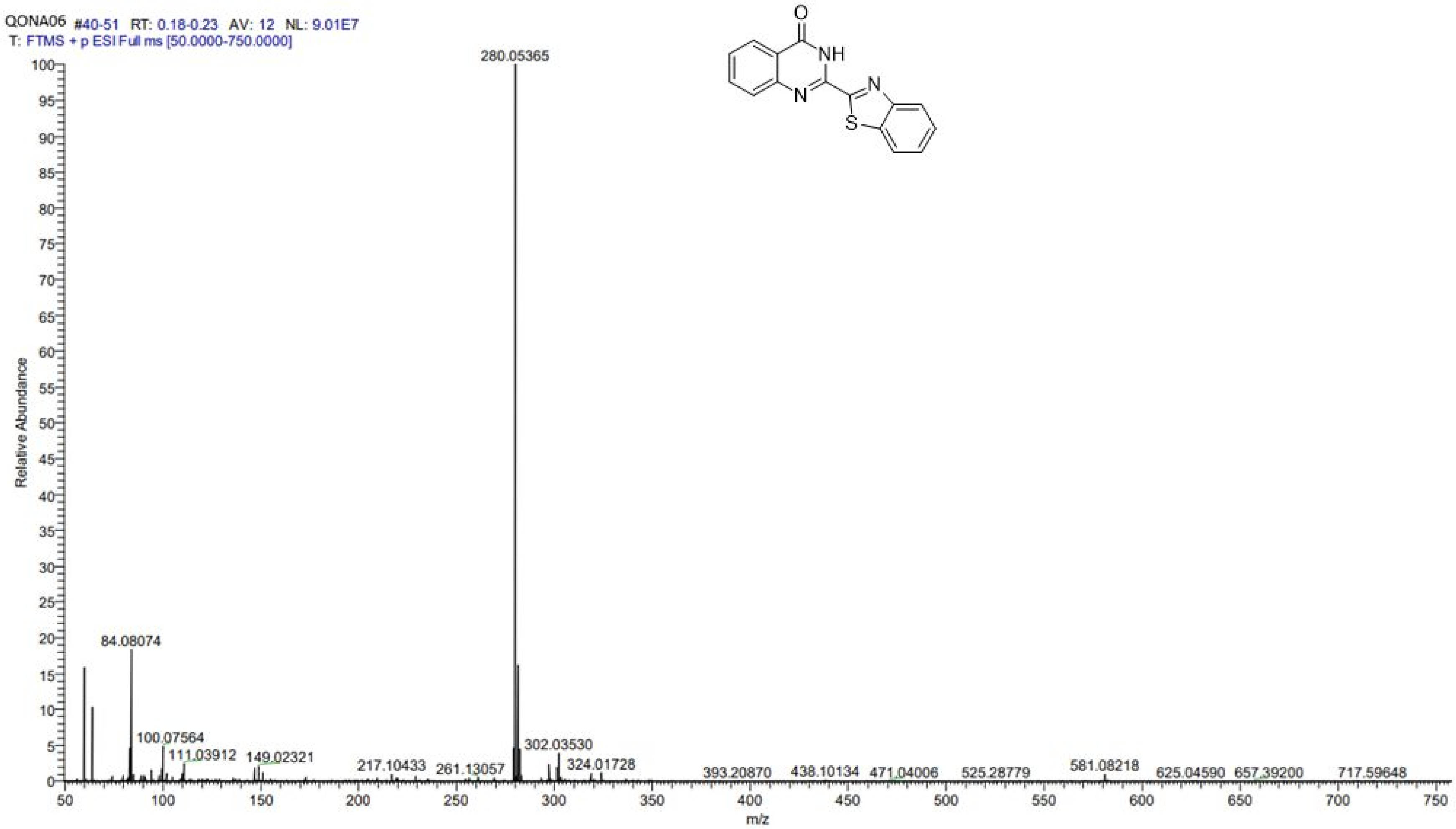

Figure 24S. MS of 2-(benzo[*d*]thiazol-2-yl)quinazolin-4(3*H*)-one, **6**.

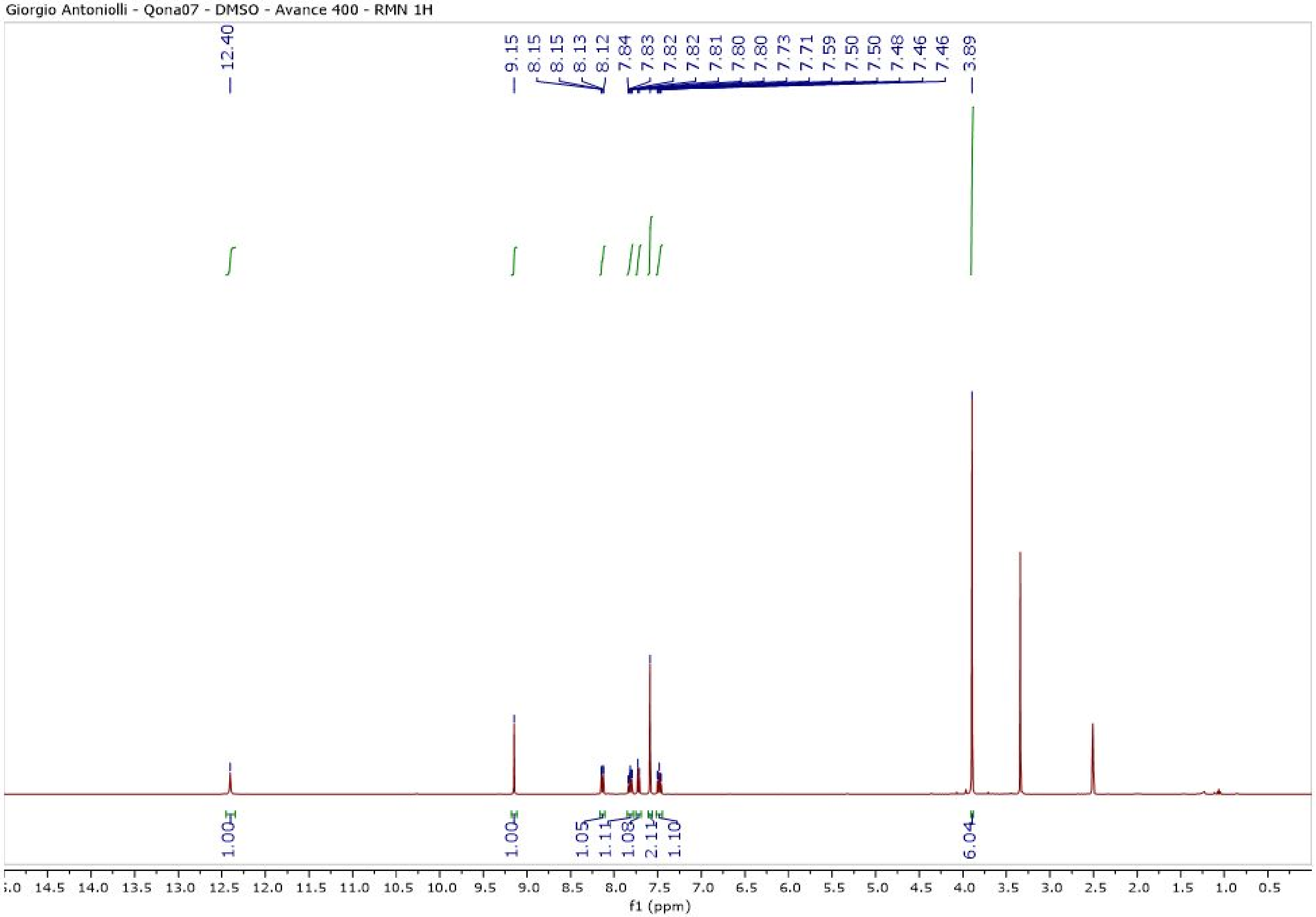

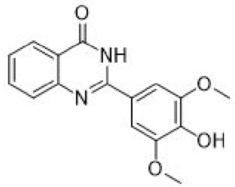

Figure 25S. ^{1}H NMR (d_6-DMSO, 400 MHz) of 2-(4-hydroxy-3,5-dimethoxyphenyl)quinazolin-4(3*H*)-one, **7**.

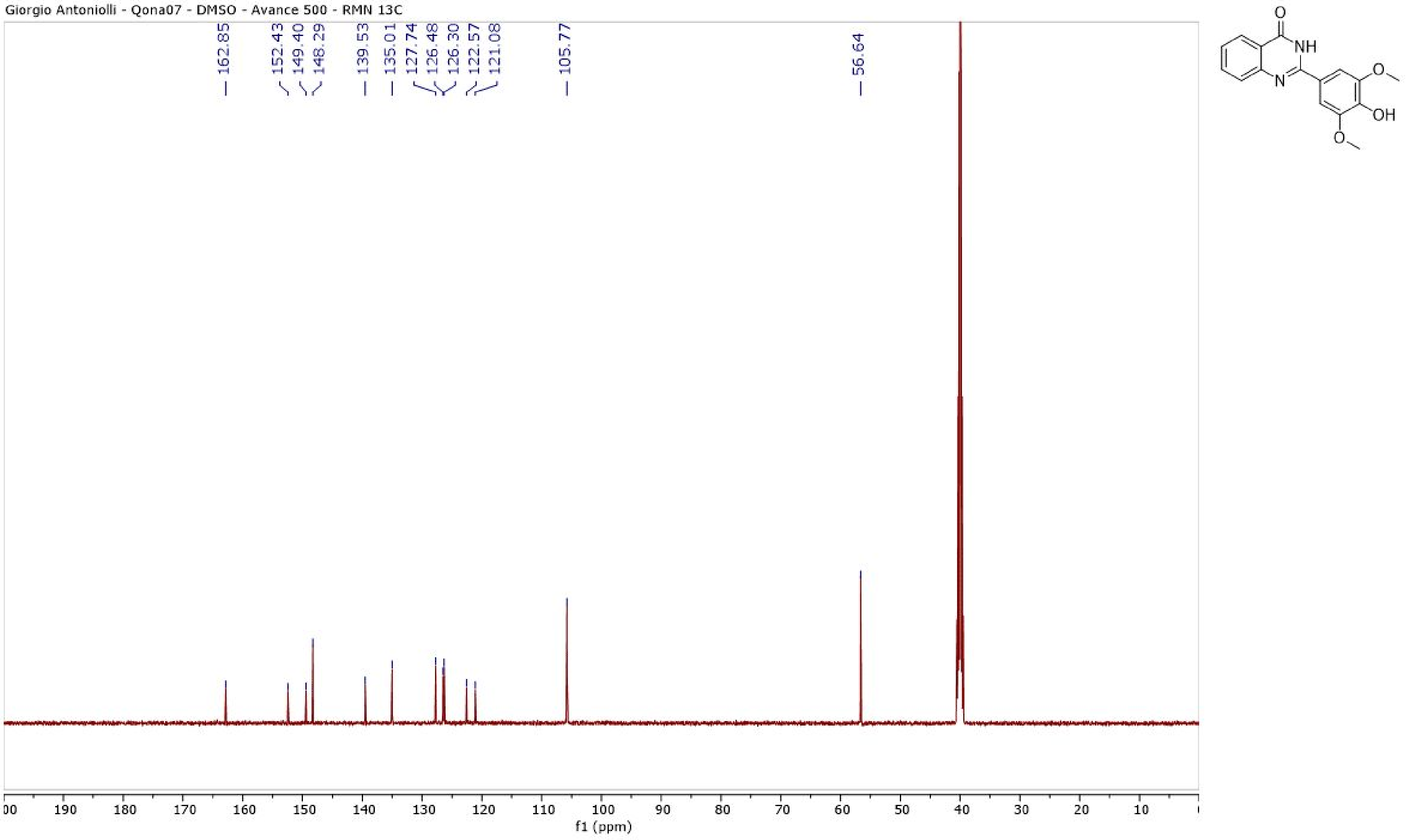

Figure 26S. ^{13}C NMR (d_6-DMSO, 125 MHz) of 2-(4-hydroxy-3,5-dimethoxyphenyl)quinazolin-4(3*H*)-one, **7**.

Sample ID:	Qona07	Method Name:	PADRAO ATR
Sample Scans:	64	User:	Admin
Background Scans:	64	Date/Time:	17-Aug-23 2:54:02PM
Resolution:	4 cm-1	Range:	4,000.00 - 650.00
System Status:	Good	Apodization:	Happ-Genzel
File Location:	C:\Program Files\Agilent\MicroLab PC\Results\Qona07_2023-08-17T14-55-27.a2r		

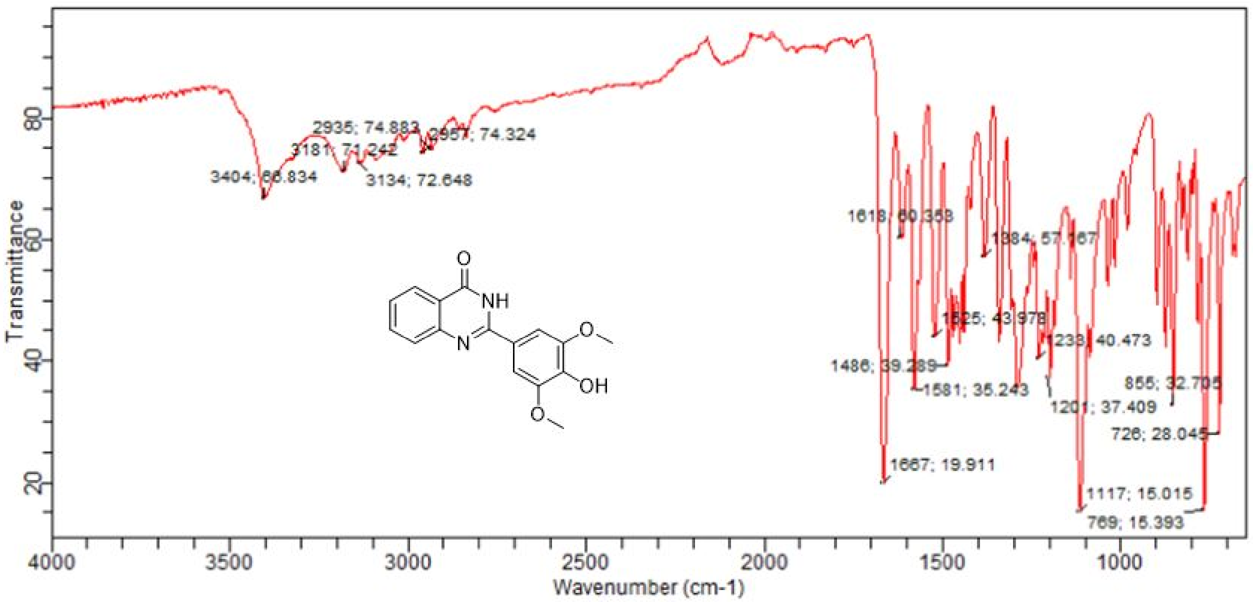

Figure 27S. IR (ATR, ν_{max}, cm^{-1}) of 2-(4-hydroxy-3,5-dimethoxyphenyl)quinazolin-4(3*H*)-one, **7**.

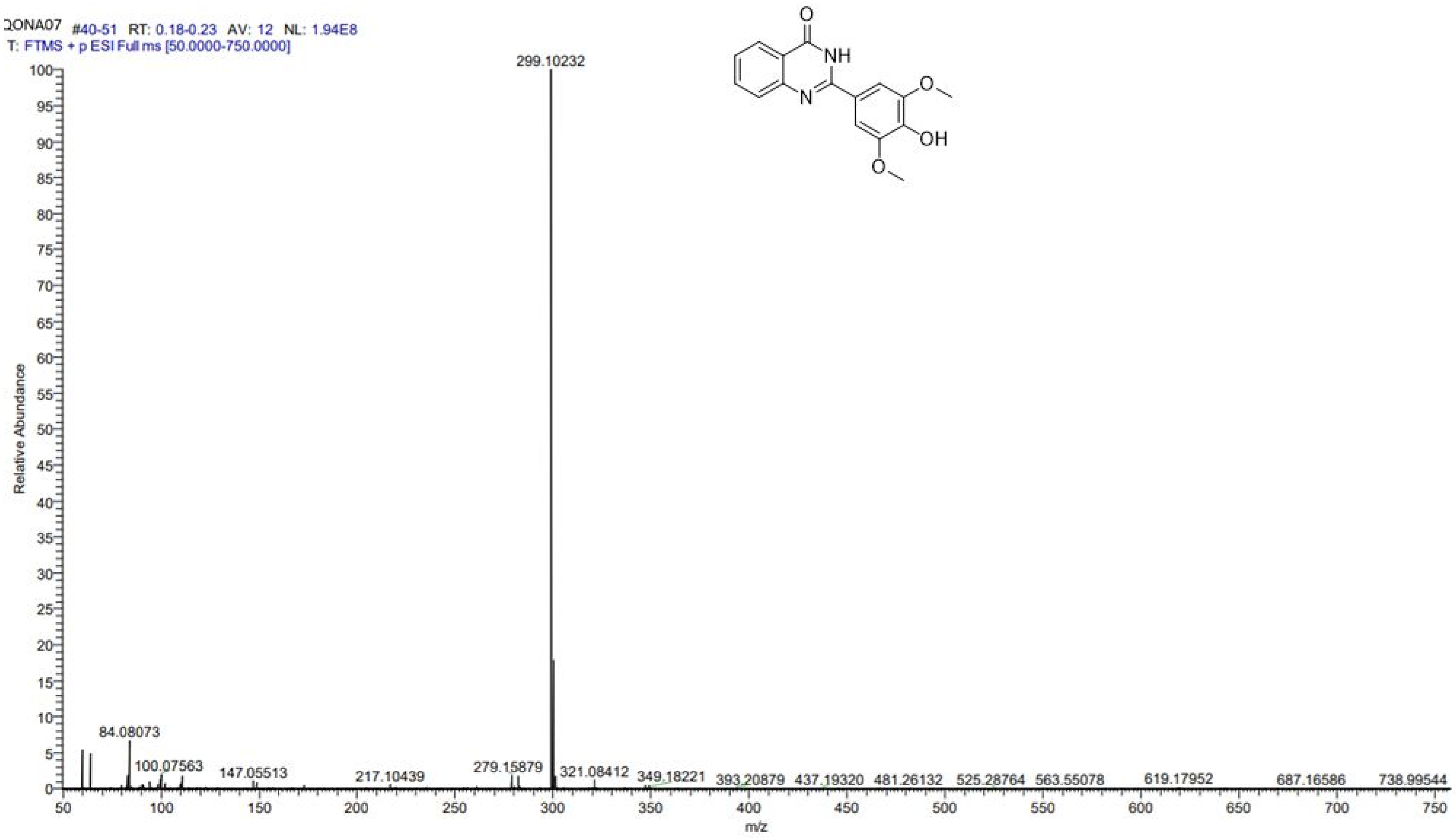

Figure 28S. MS of 2-(4-hydroxy-3,5-dimethoxyphenyl)quinazolin-4(3*H*)-one, **7**.

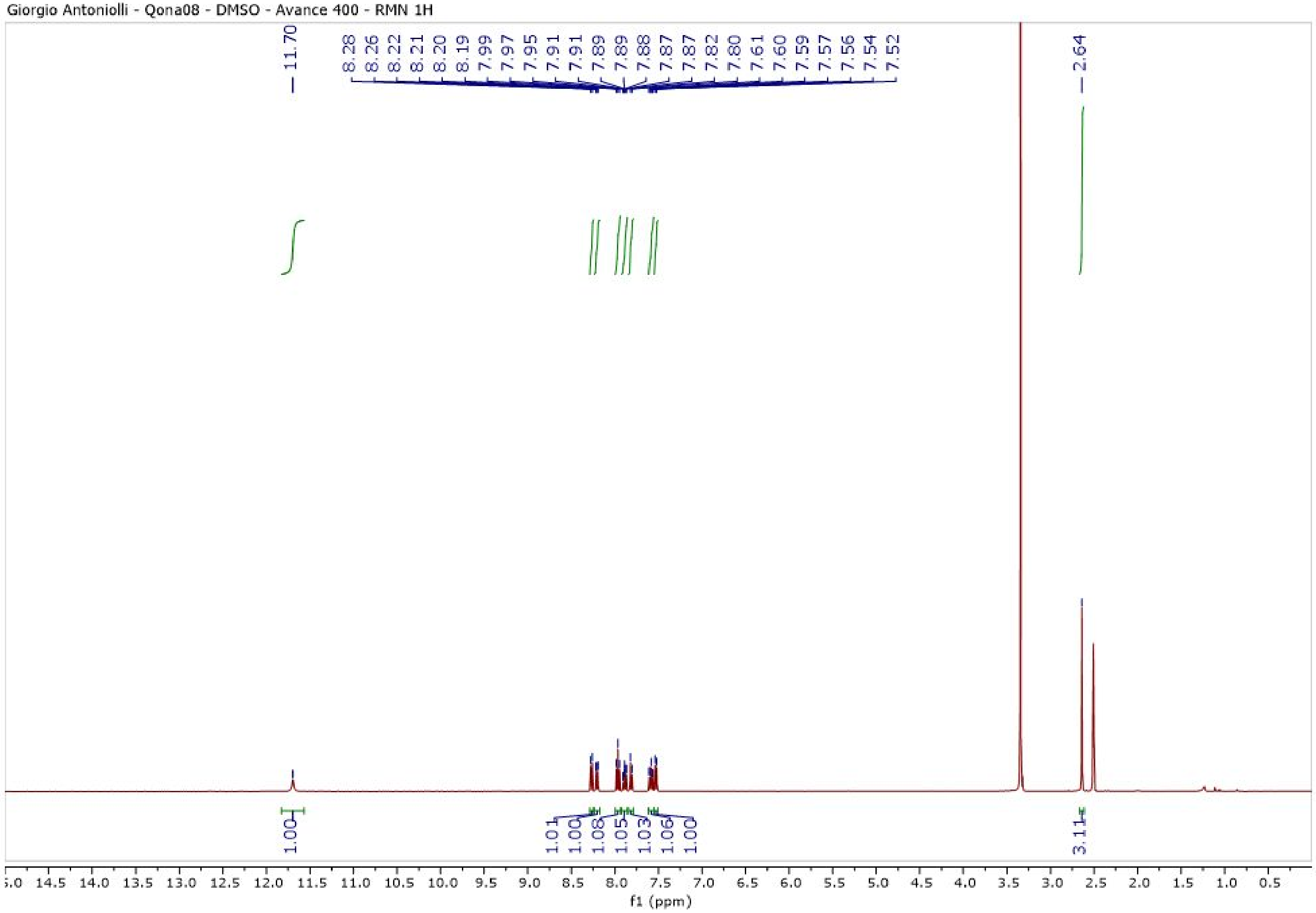

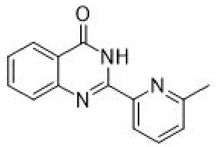

Figure 29S. ^{1}H NMR (d_6-DMSO, 400 MHz) of 2-(6-methylpyridin-2-yl)quinazolin-4(3*H*)-one, **8**.

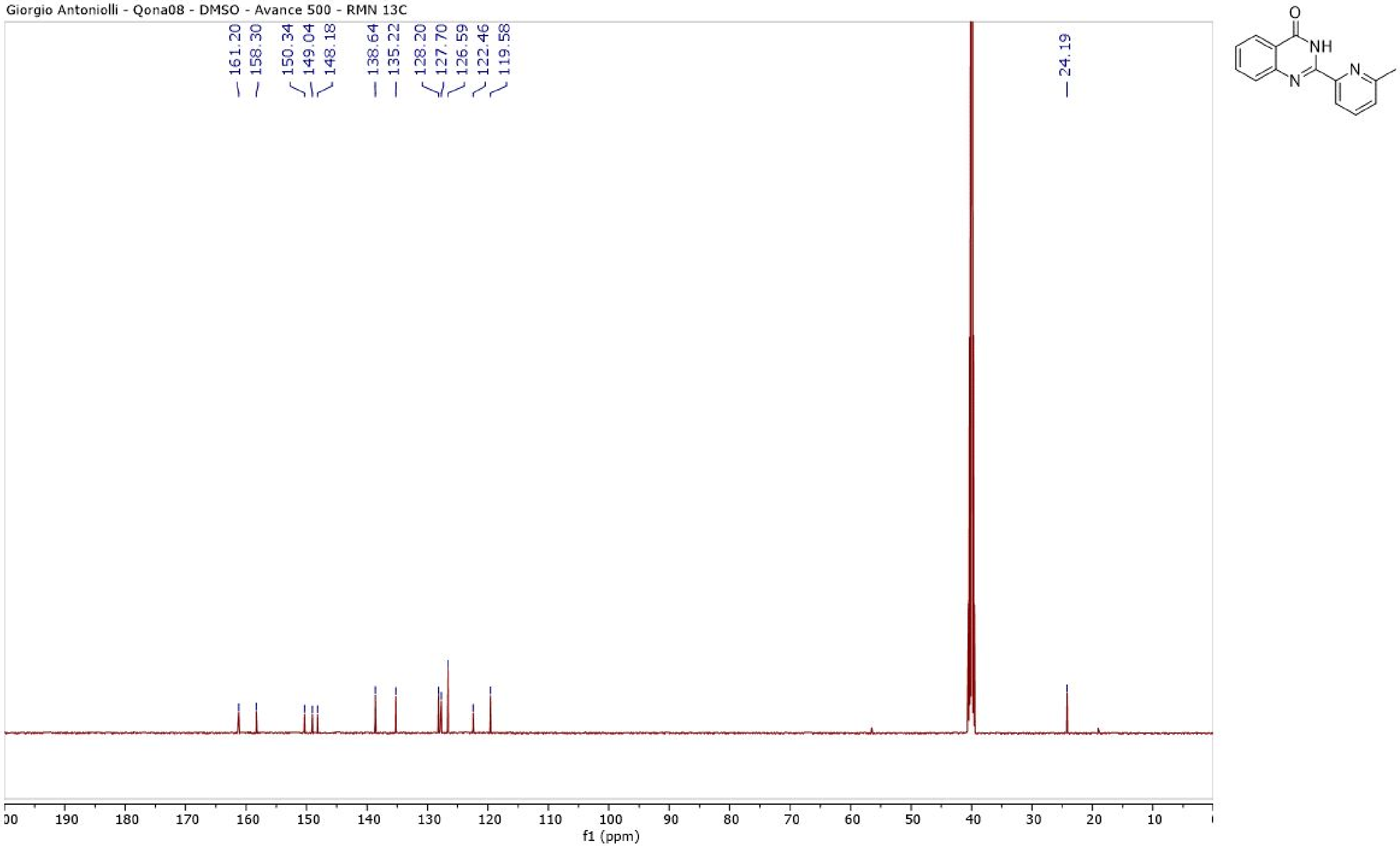

Figure 30S. ^{13}C NMR (d$_6$-DMSO, 125 MHz) of 2-(6-methylpyridin-2-yl)quinazolin-4(3*H*)-one, **8**.

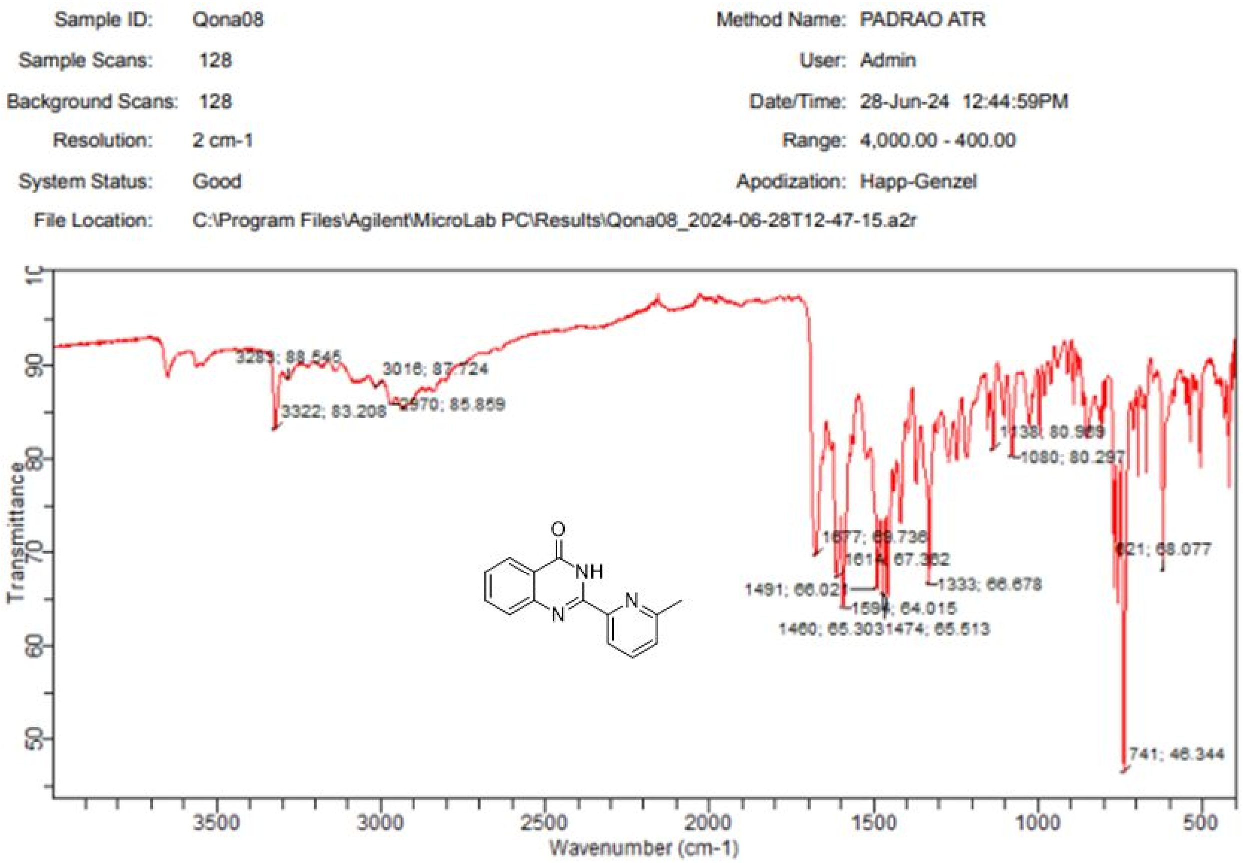

Figure 31S. IR (ATR, ν_{max}, cm^{-1}) of 2-(6-methylpyridin-2-yl)quinazolin-4(3*H*)-one, **8**.

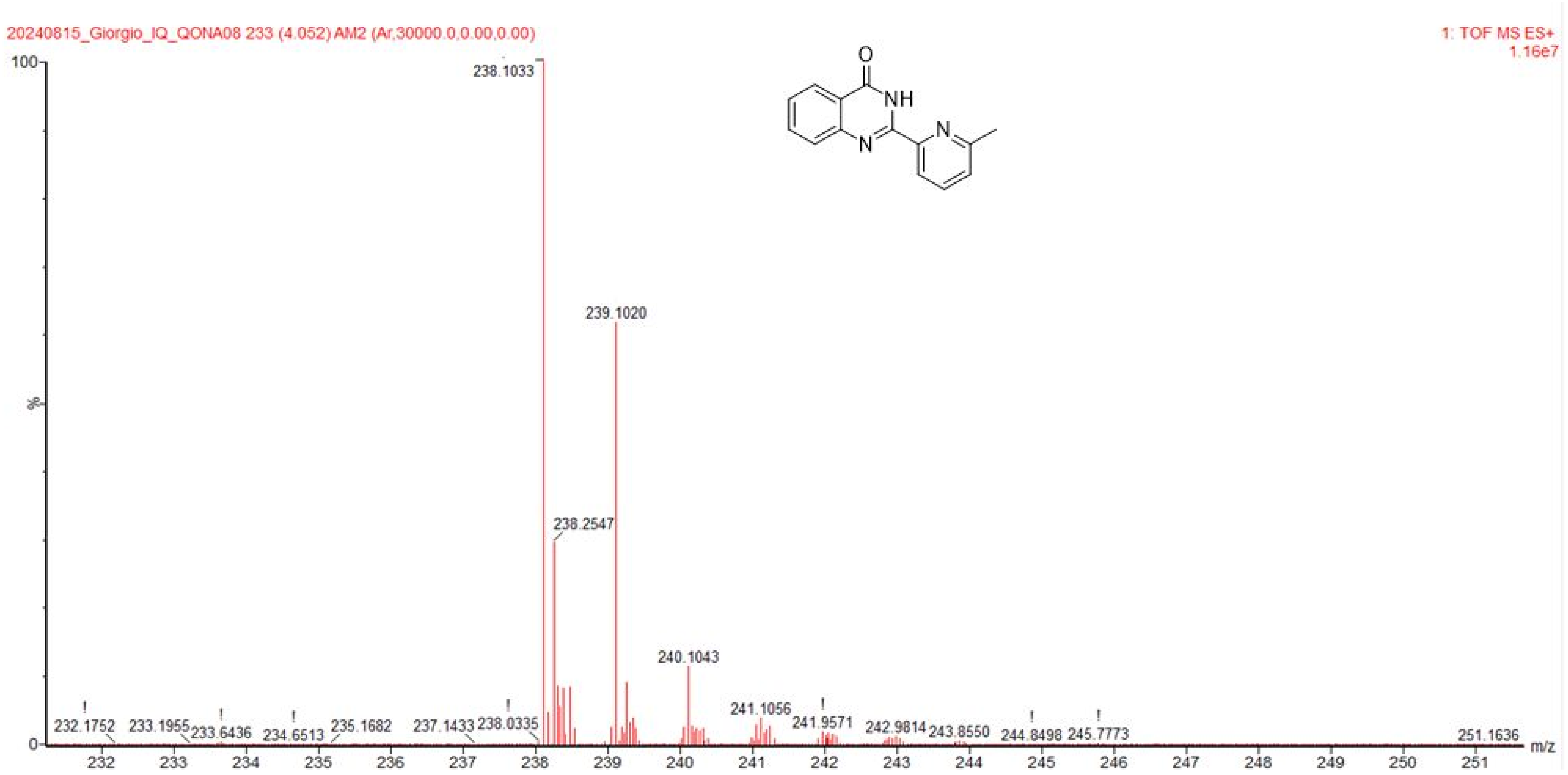

Figure 32S. MS of 2-(6-methylpyridin-2-yl)quinazolin-4(3*H*)-one, **8**.

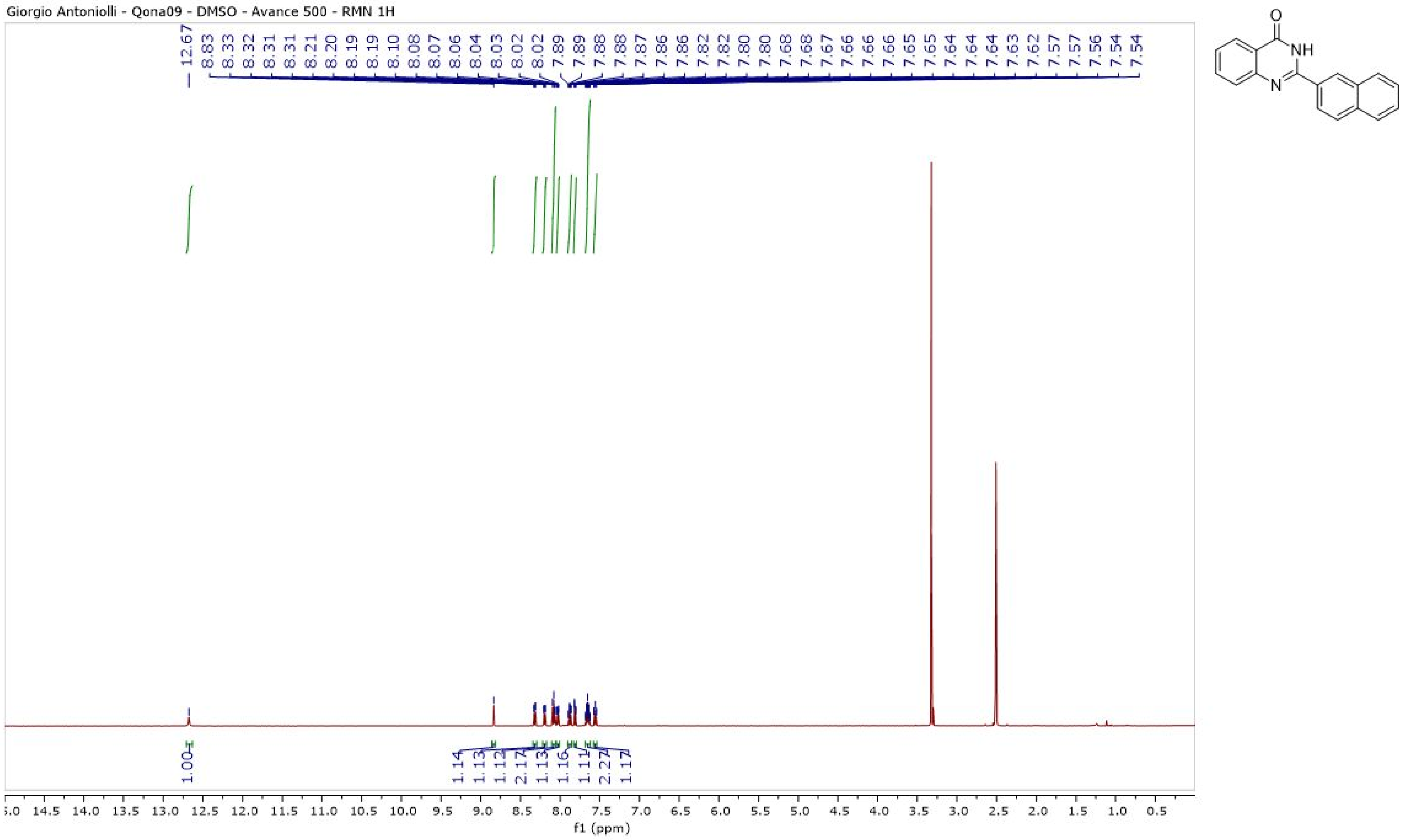

Figure 33S. ^{1}H NMR (d_6-DMSO, 500 MHz) of 2-(naphthalen-2-yl)quinazolin-4(3*H*)-one, **9**.

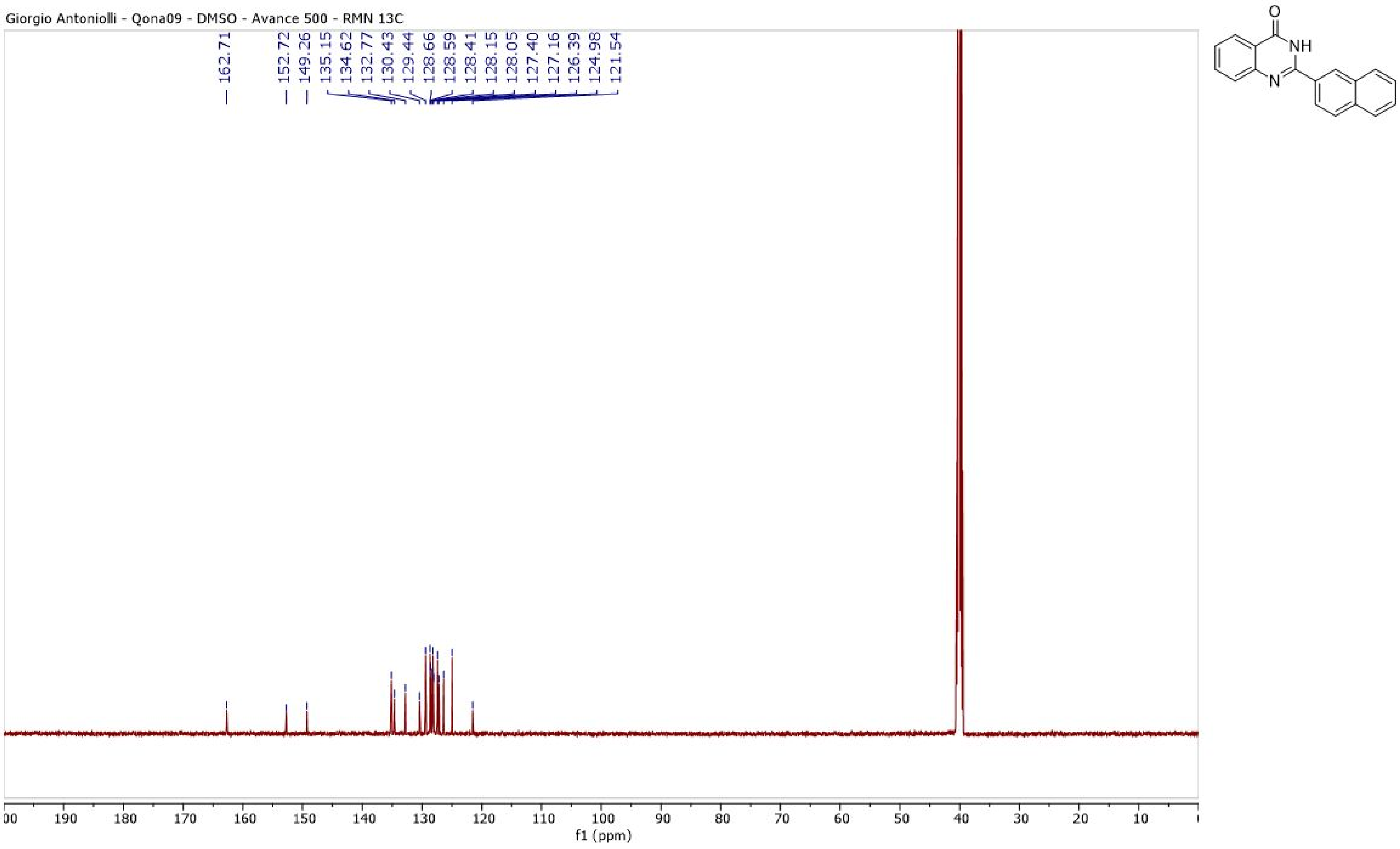

Figure 34S. ^{13}C NMR (d_6-DMSO, 125 MHz) of 2-(naphthalen-2-yl)quinazolin-4(3*H*)-one, **9**.

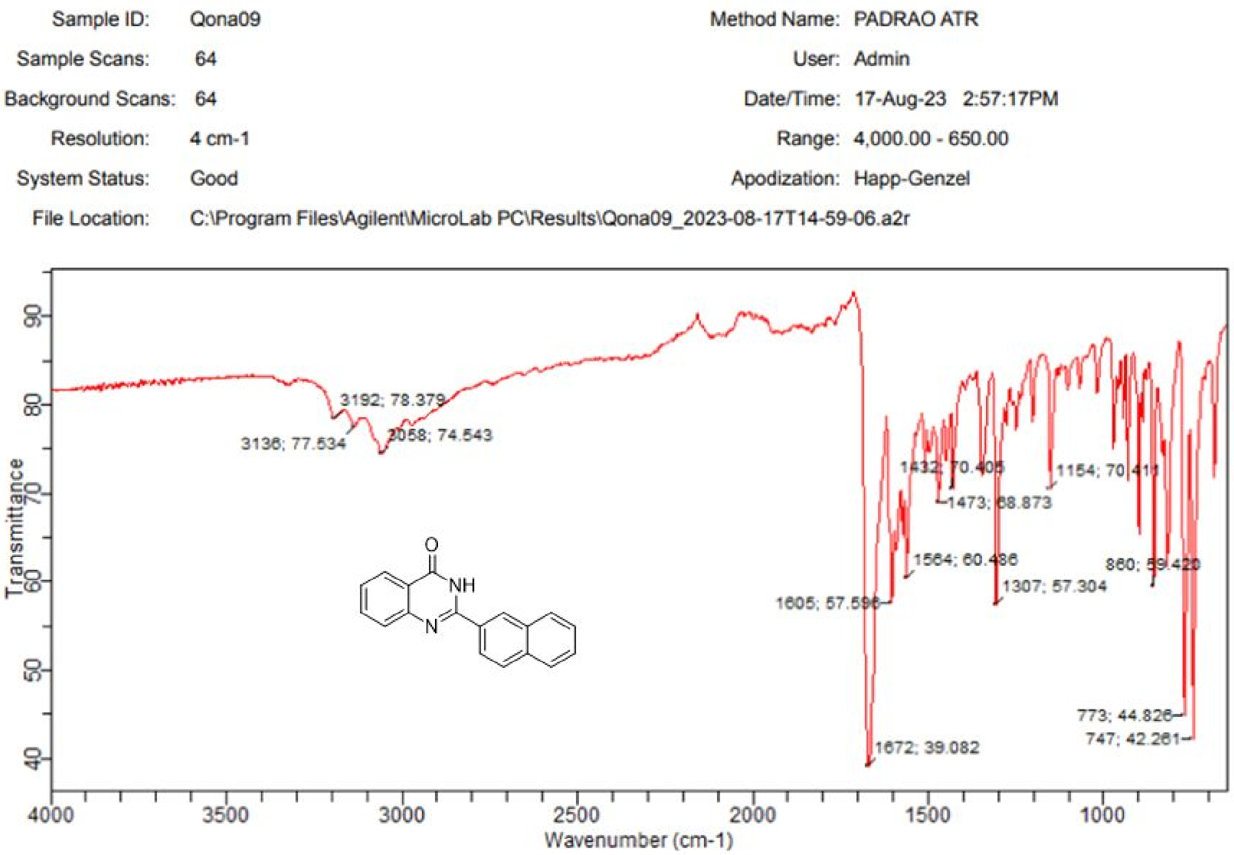

Figure 35S. IR (ATR, ν_{max}, cm^{-1}) of 2-(naphthalen-2-yl)quinazolin-4(3*H*)-one, **9**.

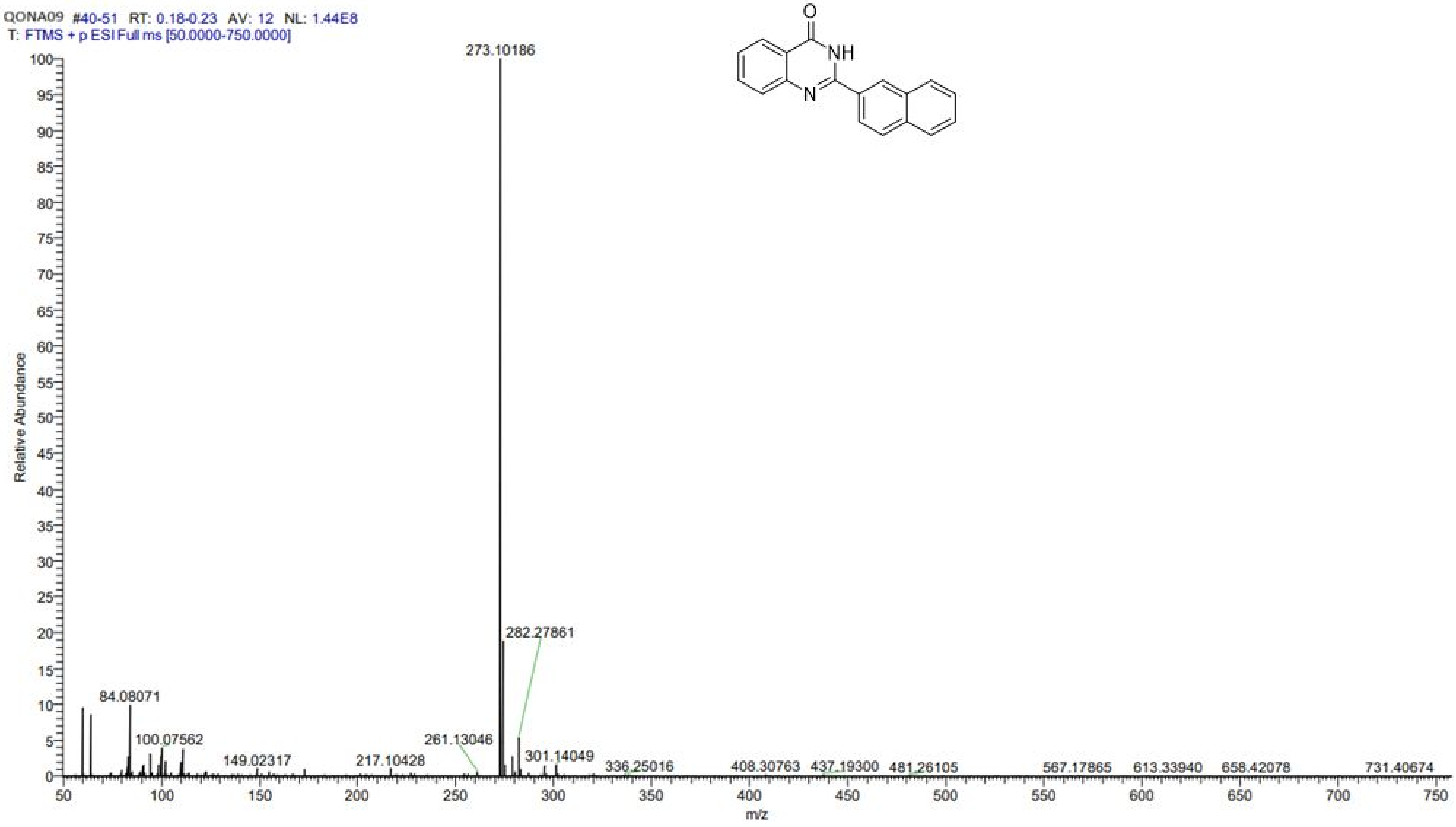

Figure 36S. MS of 2-(naphthalen-2-yl)quinazolin-4(3*H*)-one, **9**.

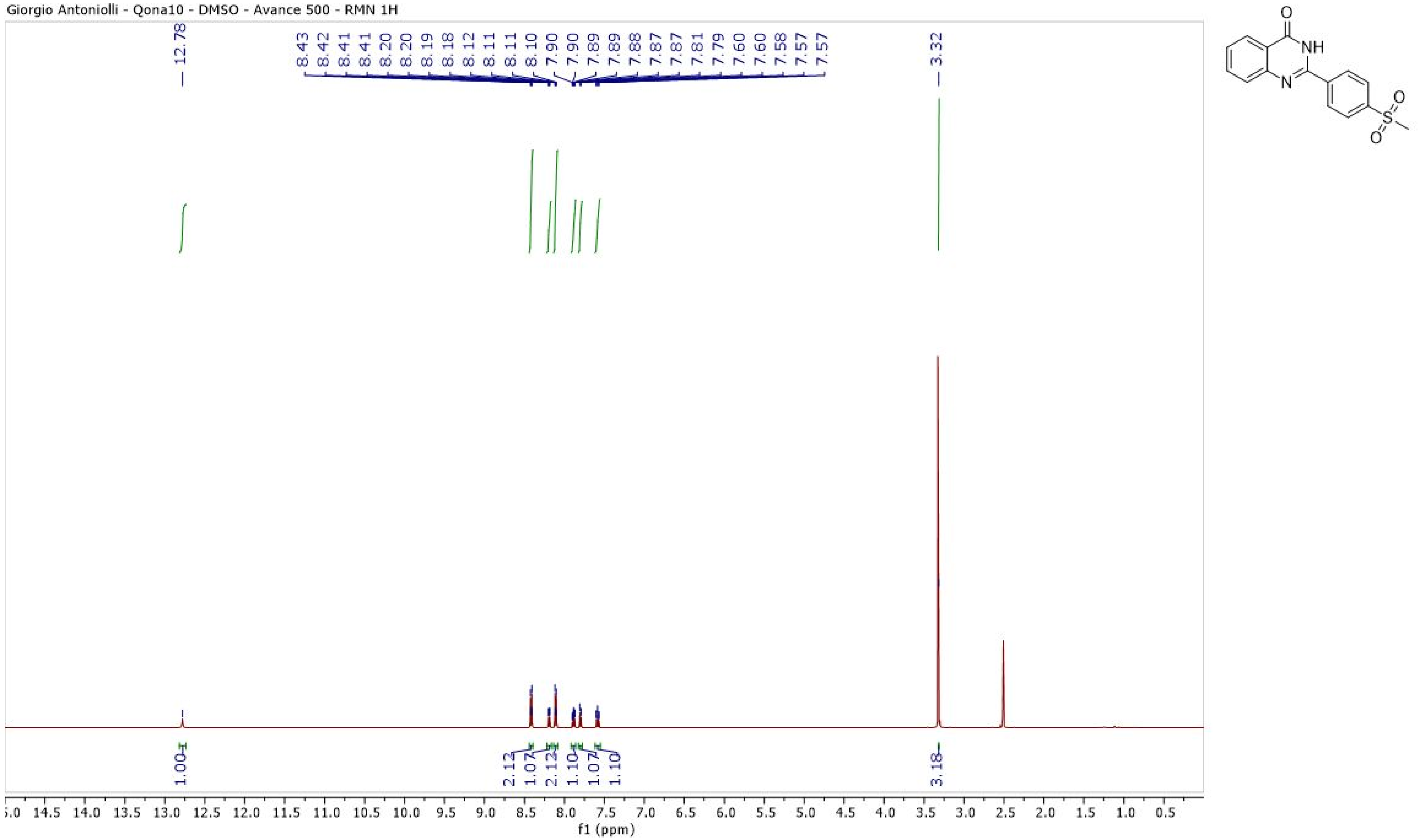

Figure 37S. ^{1}H NMR (d$_6$-DMSO, 500 MHz) of 2-(4-(methylsulfonyl)phenyl)quinazolin-4(3*H*)-one, **10**.

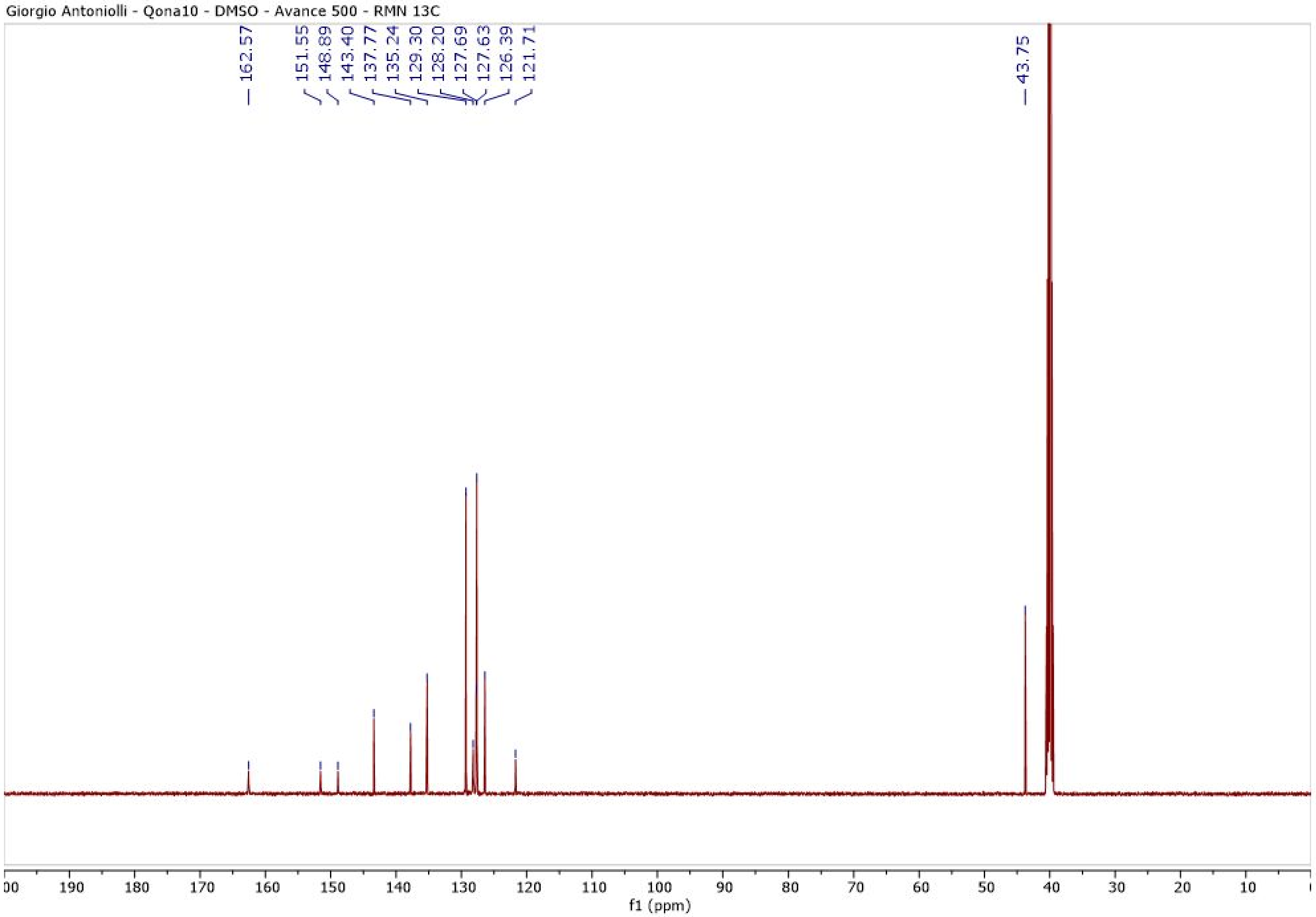

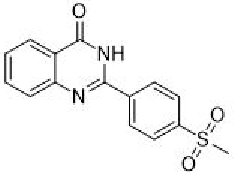

Figure 38S. ^{13}C NMR (d_6-DMSO, 125 MHz) of 2-(4-(methylsulfonyl)phenyl)quinazolin-4(3*H*)-one, **10**.

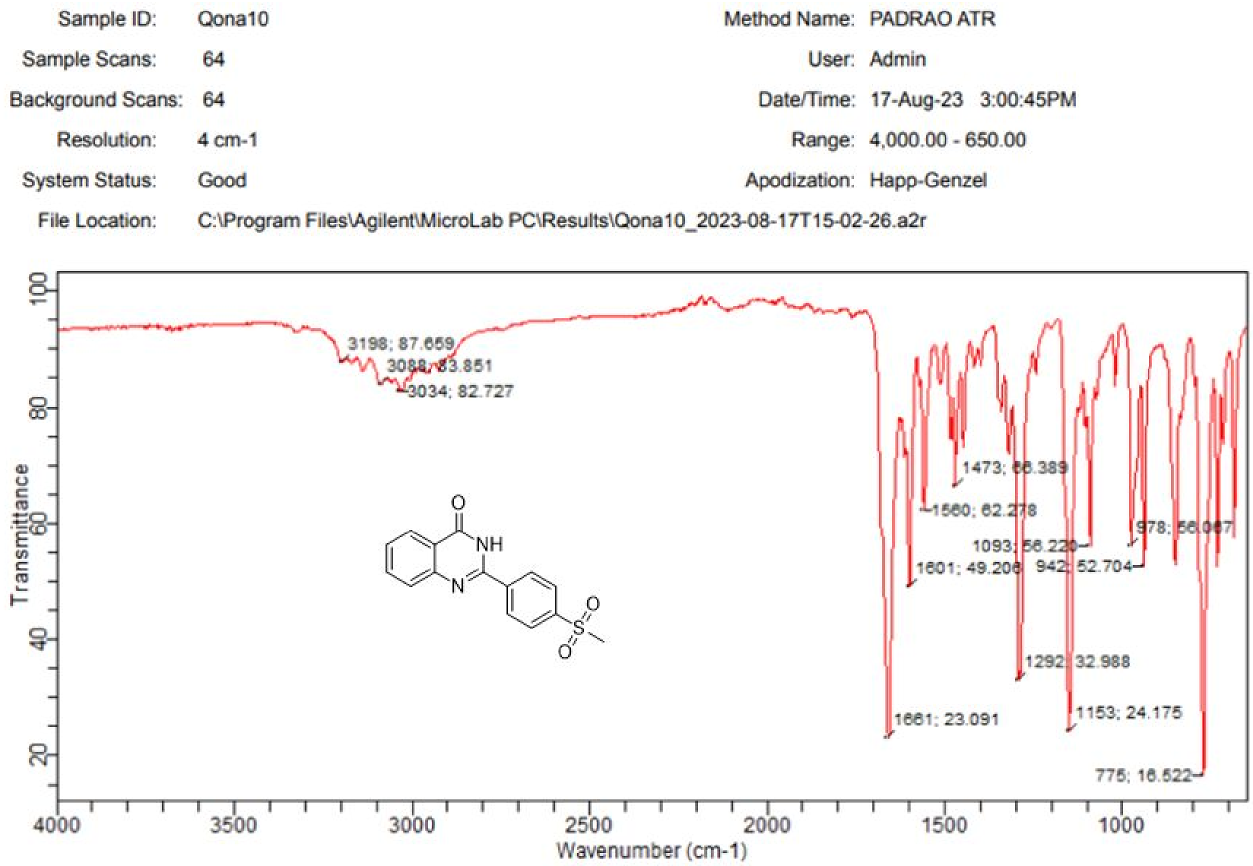

Figure 39S. IR (ATR, ν_{max}, cm^{-1}) of 2-(4-(methylsulfonyl)phenyl)quinazolin-4(3*H*)-one, **10**.

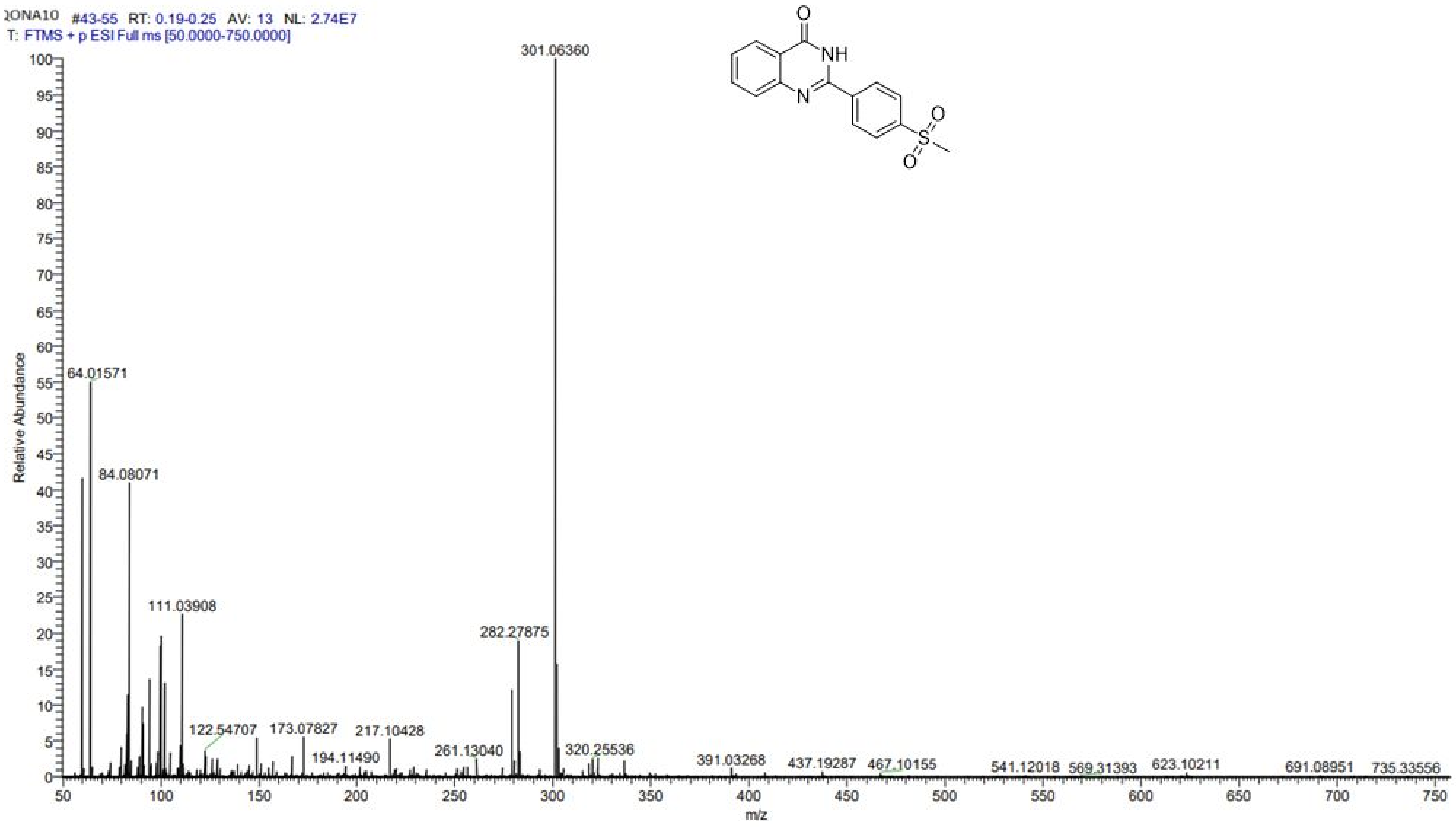

Figure 40S. MS of 2-(4-(methylsulfonyl)phenyl)quinazolin-4(3*H*)-one, **10**.

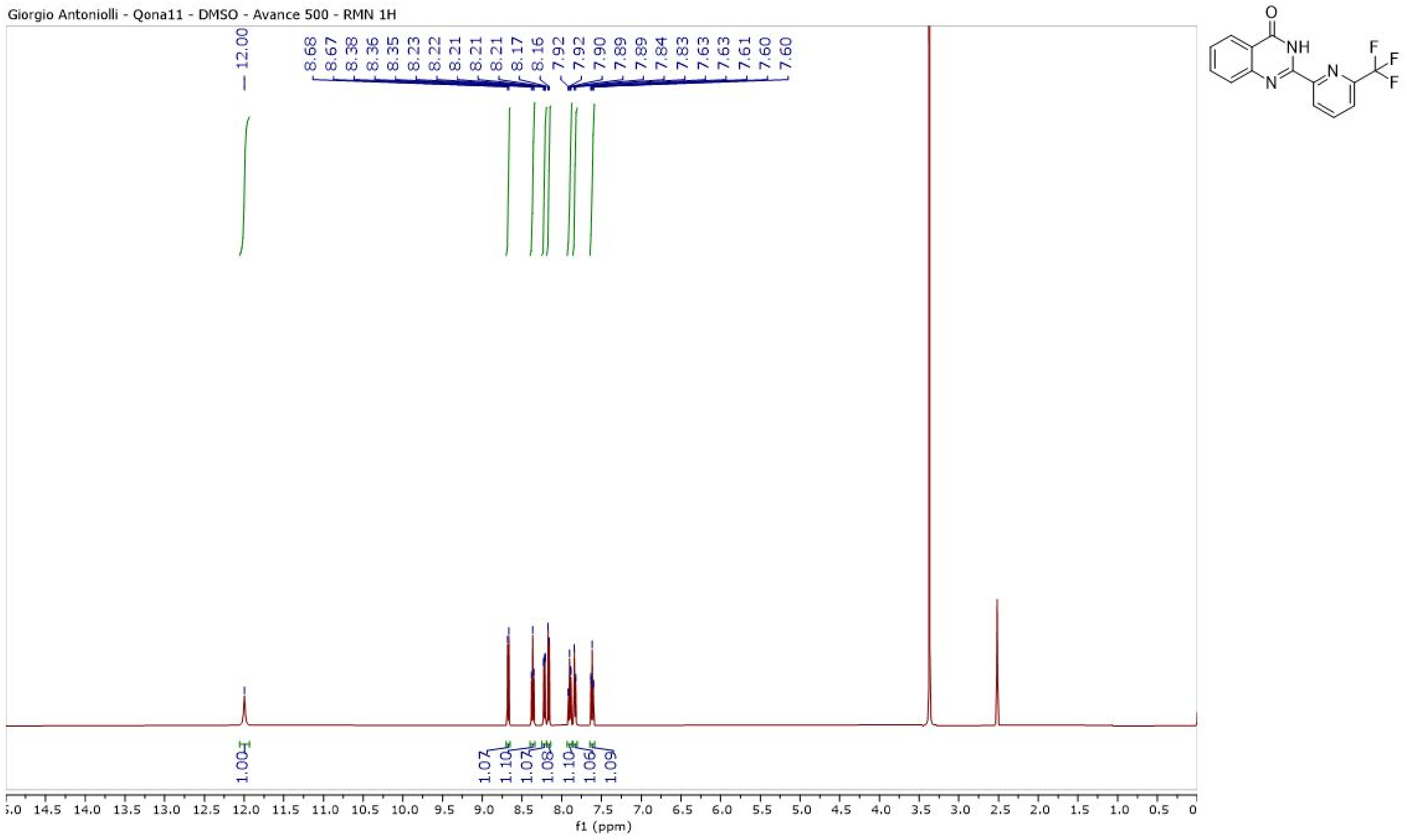

Figure 41S. ^{1}H NMR (d_6-DMSO, 500 MHz) of 2-(6-(trifluoromethyl)pyridin-2-yl)quinazolin-4(3*H*)-one, **11**.

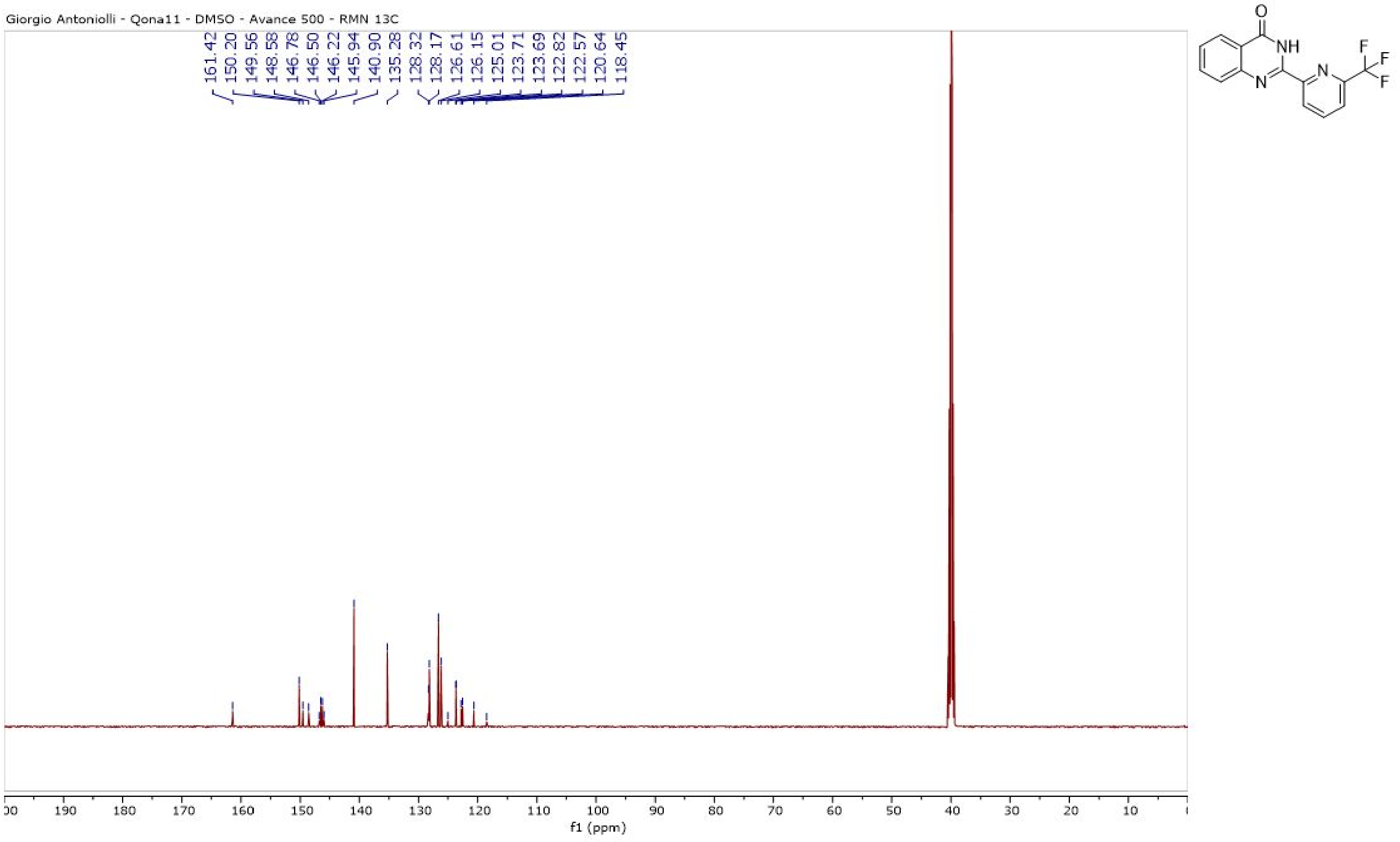

Figure 42S. ^{13}C NMR (d$_6$-DMSO, 125 MHz) of 2-(6-(trifluoromethyl)pyridin-2-yl)quinazolin-4(3*H*)-one, **11**.

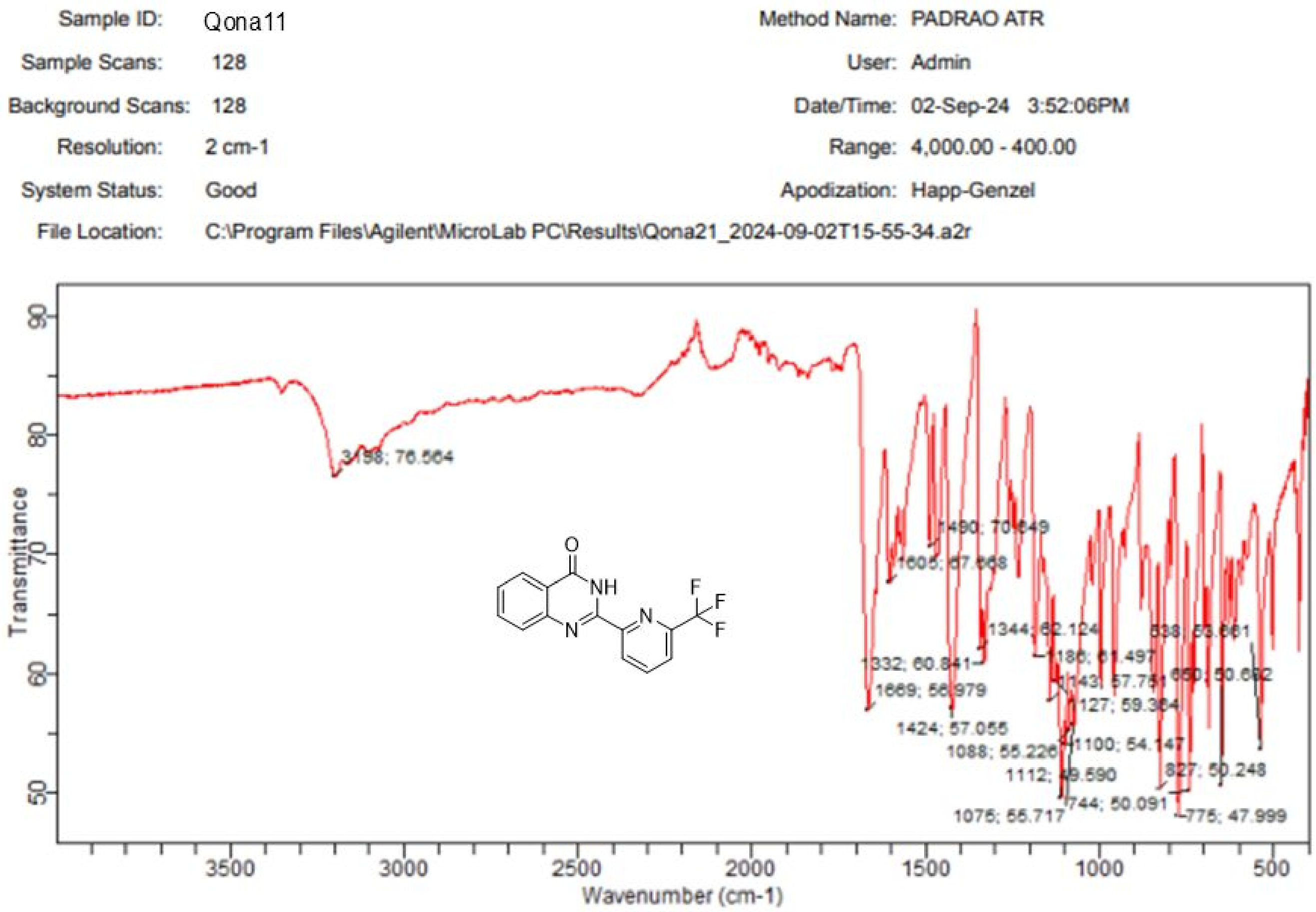

Figure 43S. IR (ATR, ν_{max}, cm^{-1}) of 2-(6-(trifluoromethyl)pyridin-2-yl)quinazolin-4(3*H*)-one, **11**.

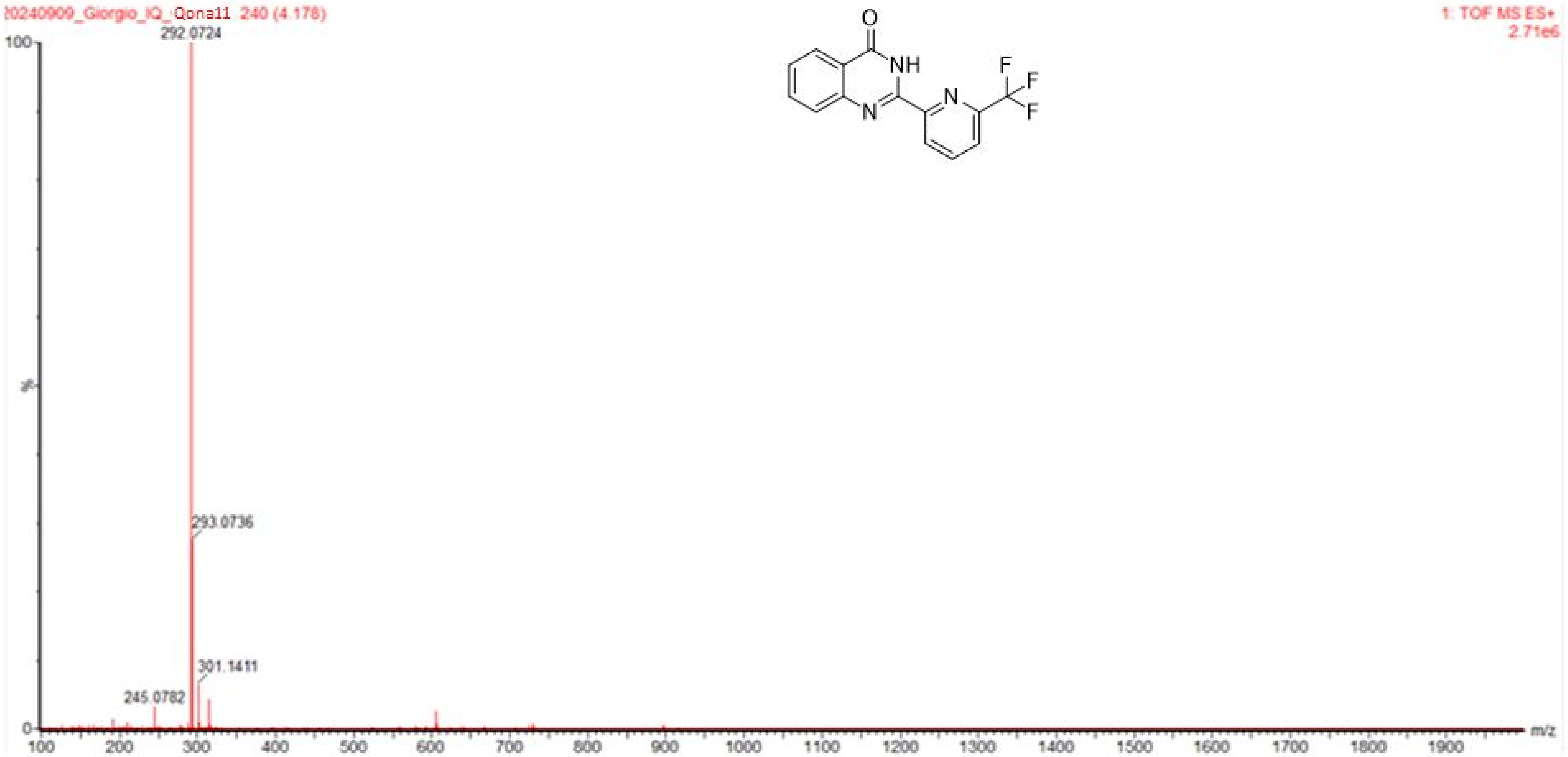

Figure 44S. MS of 2-(6-(trifluoromethyl)pyridin-2-yl)quinazolin-4(3*H*)-one, **11**.

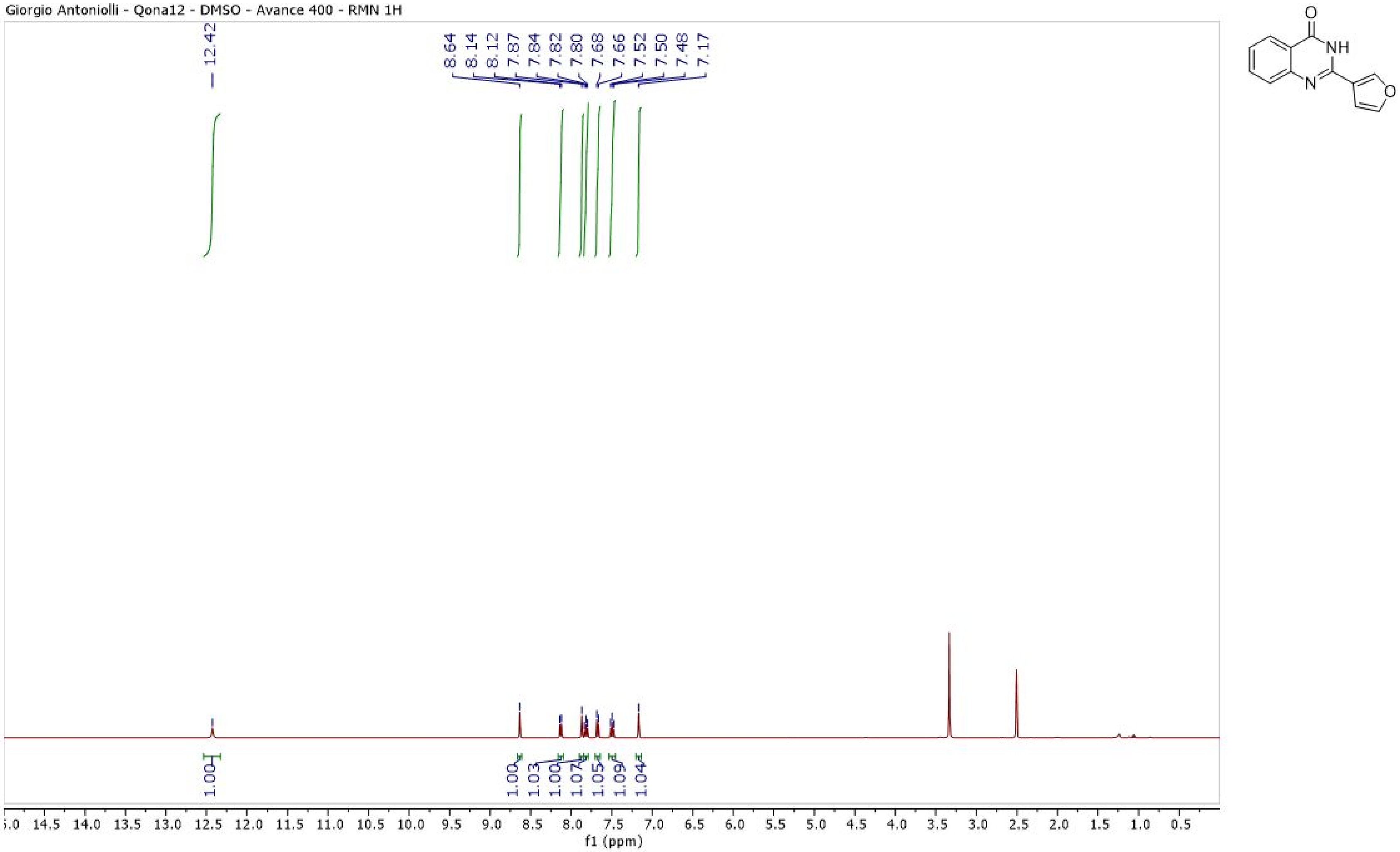

Figure 45S. ^{1}H NMR (d$_6$-DMSO, 500 MHz) of 2-(furan-3-yl)quinazolin-4(3*H*)-one, **12**.

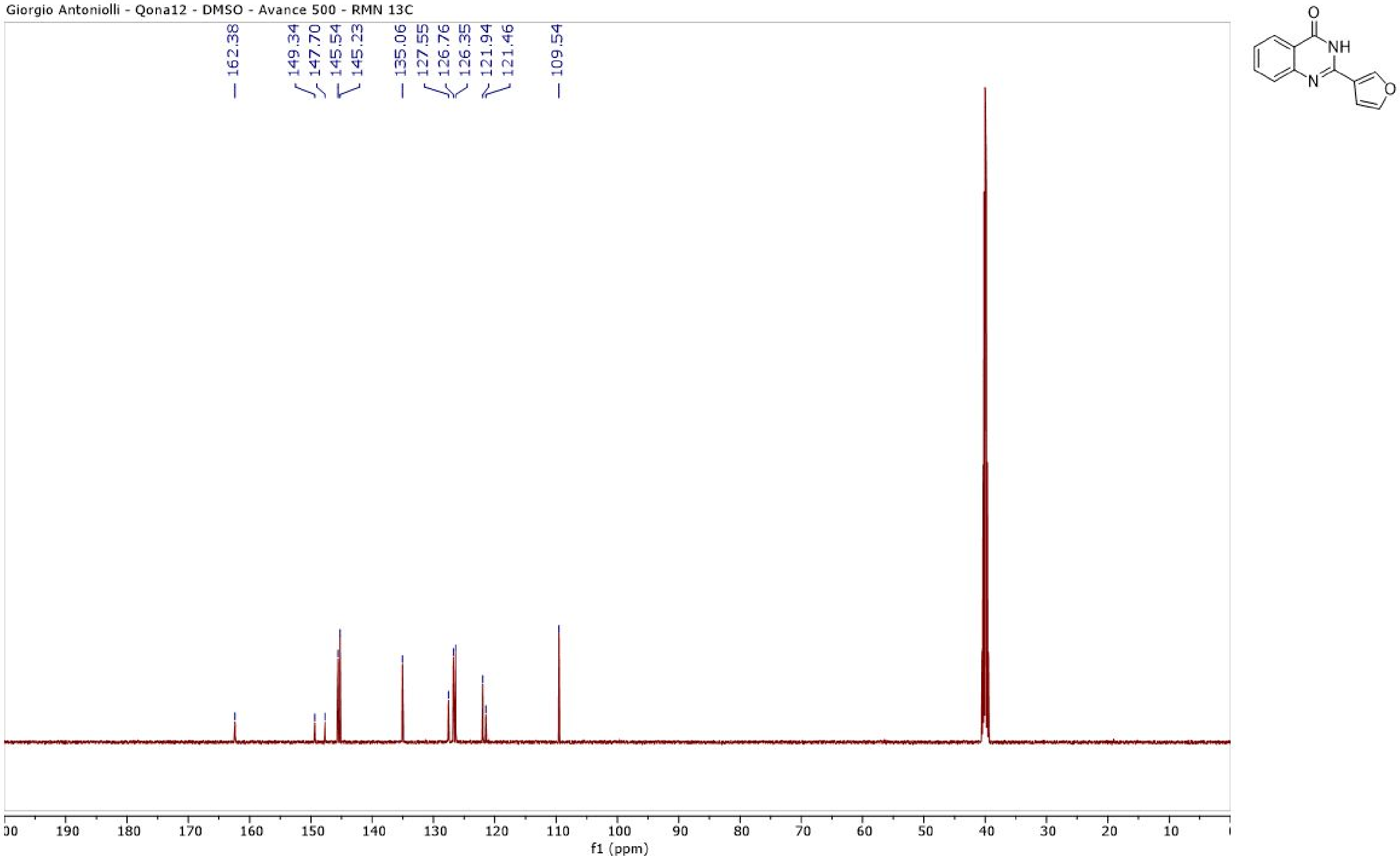

Figure 46S. ^{13}C NMR (d_6-DMSO, 125 MHz) of 2-(furan-3-yl)quinazolin-4(3*H*)-one, **12**.

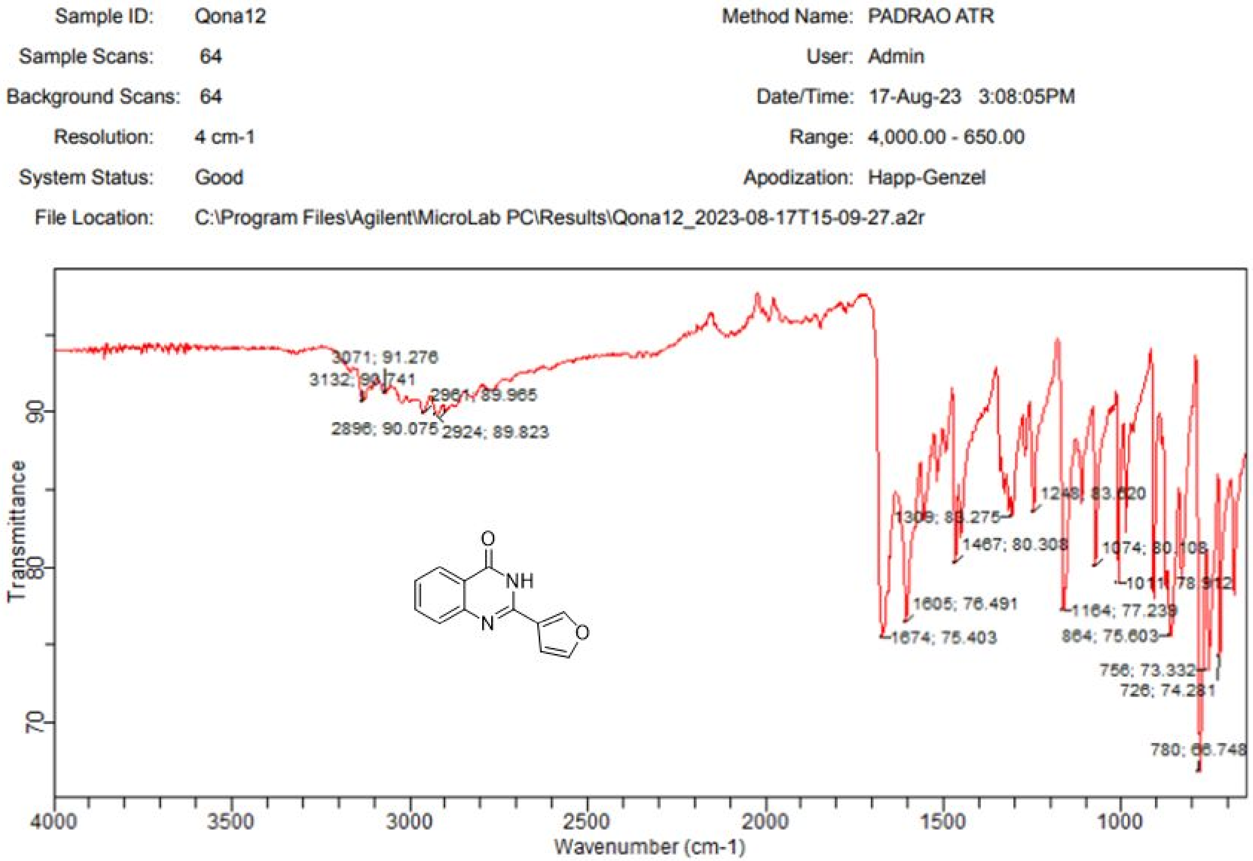

Figure 47S. IR (ATR, ν_{max}, cm^{-1}) of 2-(furan-3-yl)quinazolin-4(3*H*)-one, **12**.

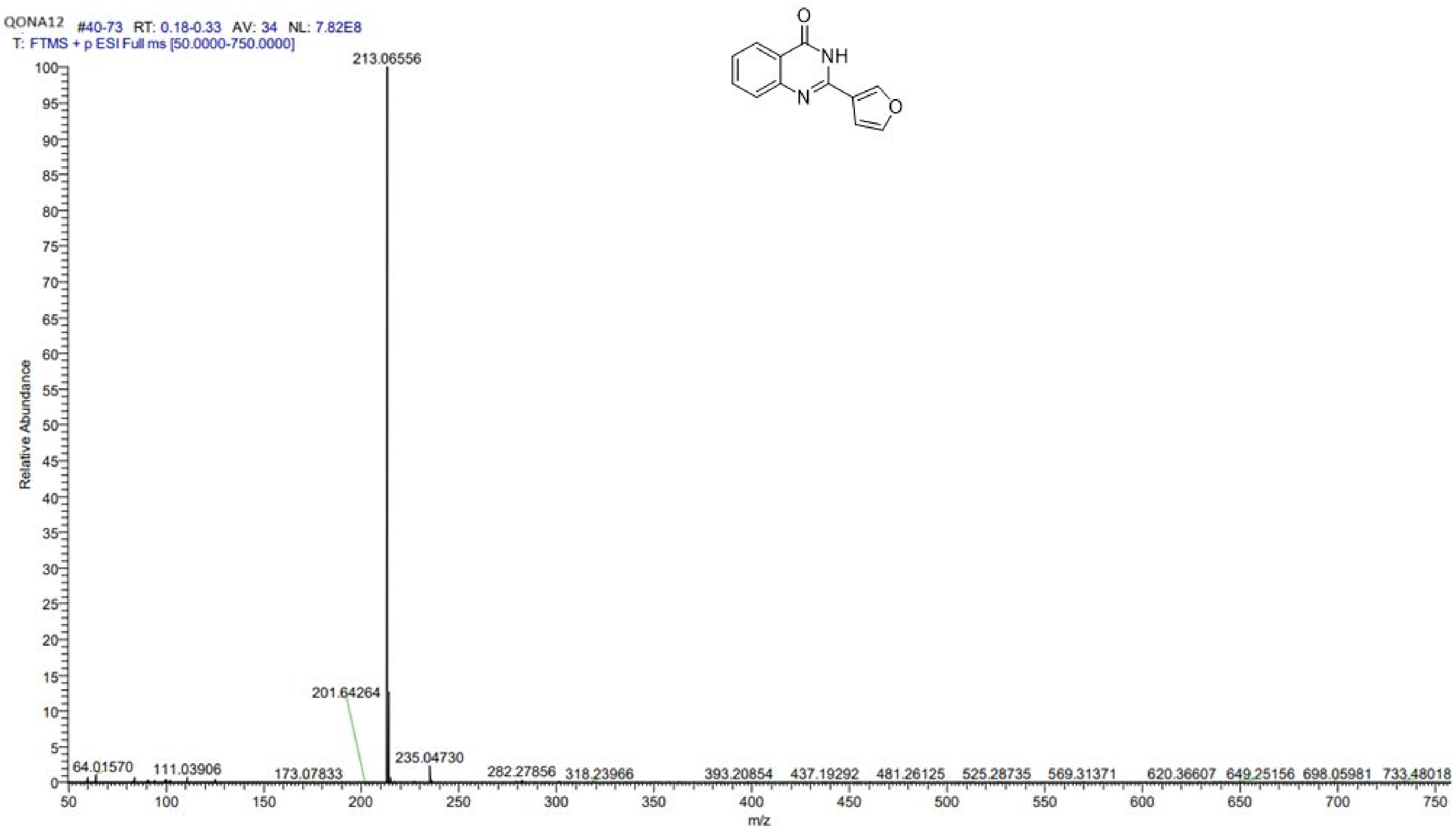

Figure 48S. MS of 2-(furan-3-yl)quinazolin-4(3*H*)-one, **12**.

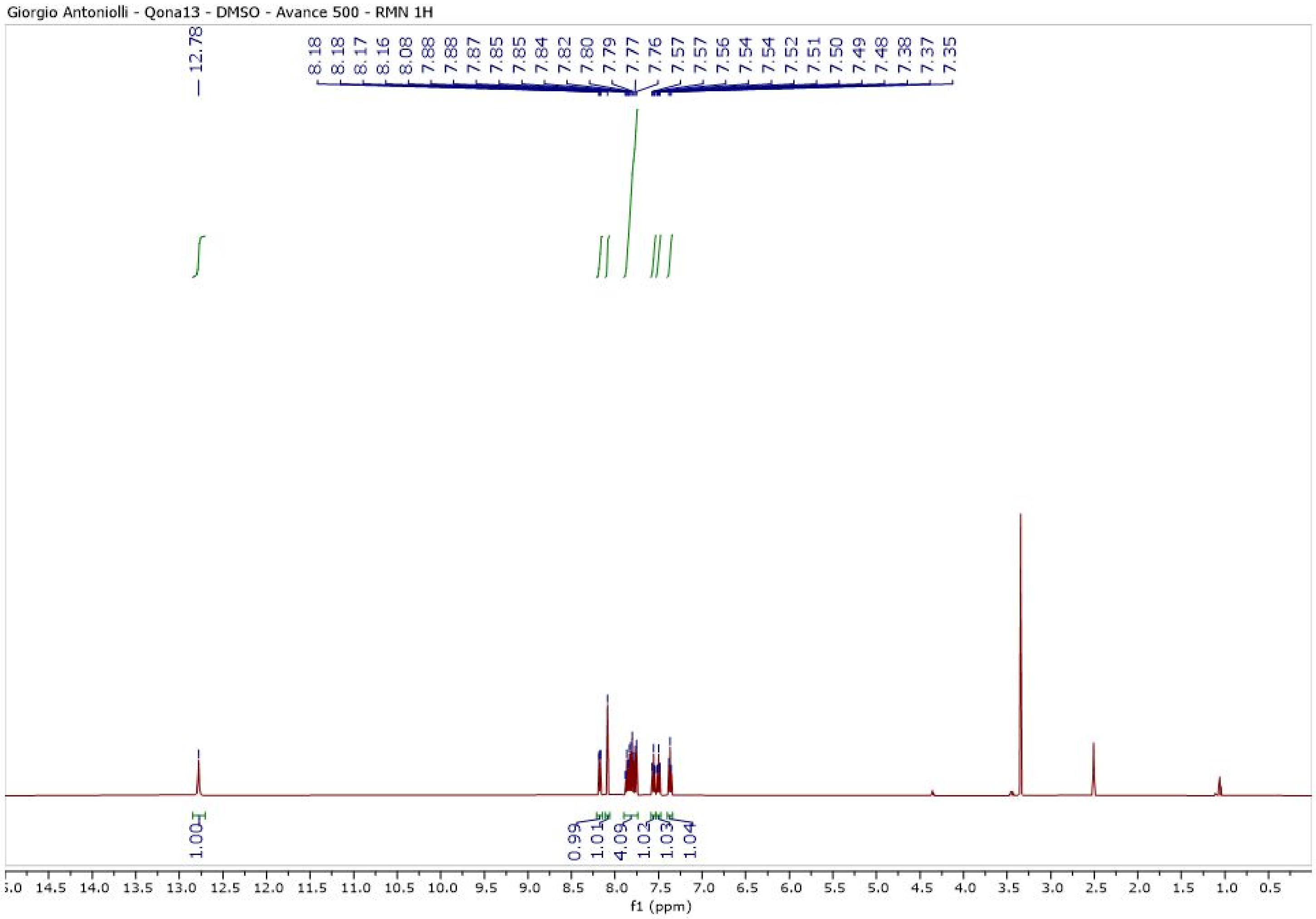

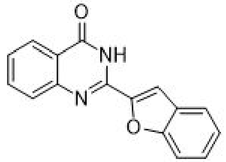

Figure 49S. ^{1}H NMR (d_6-DMSO, 500 MHz) of 2-(benzofuran-2-yl)quinazolin-4(3*H*)-one, **13**.

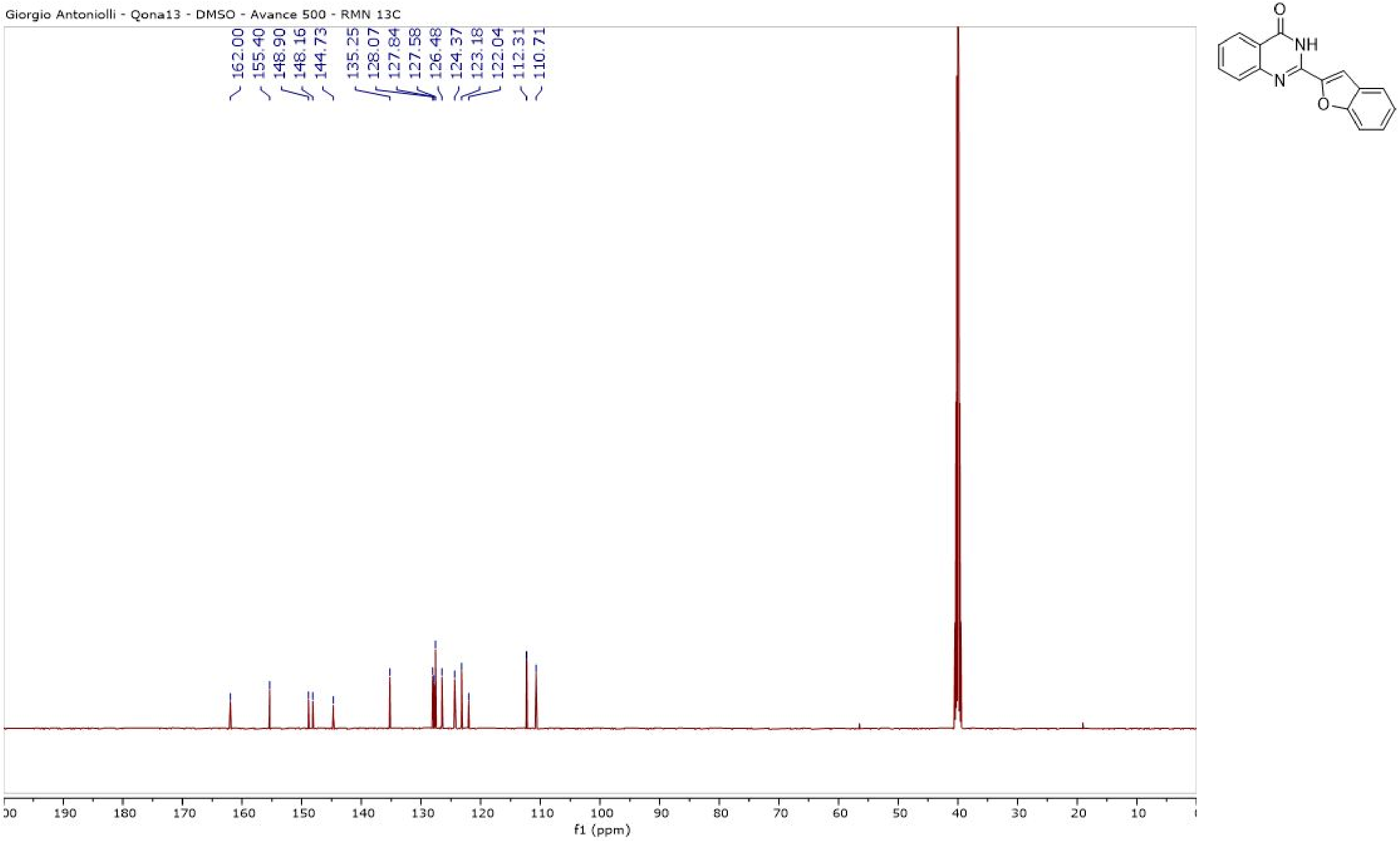

Figure 50S. ^{13}C NMR (d$_6$-DMSO, 125 MHz) of 2-(benzofuran-2-yl)quinazolin-4(3*H*)-one, **13**.

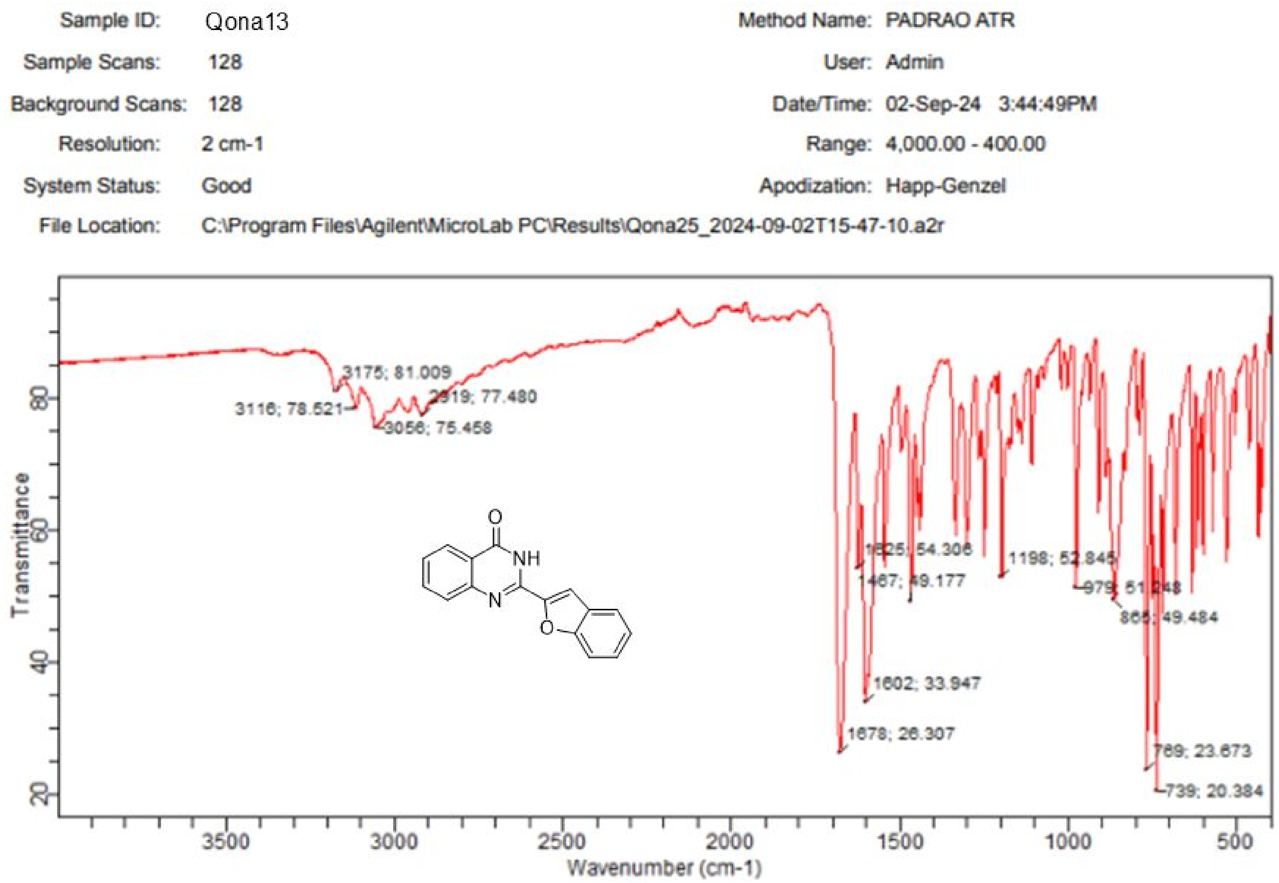

Figure 51S. IR (ATR, ν_{max}, cm^{-1}) of 2-(benzofuran-2-yl)quinazolin-4(3*H*)-one, **13**.

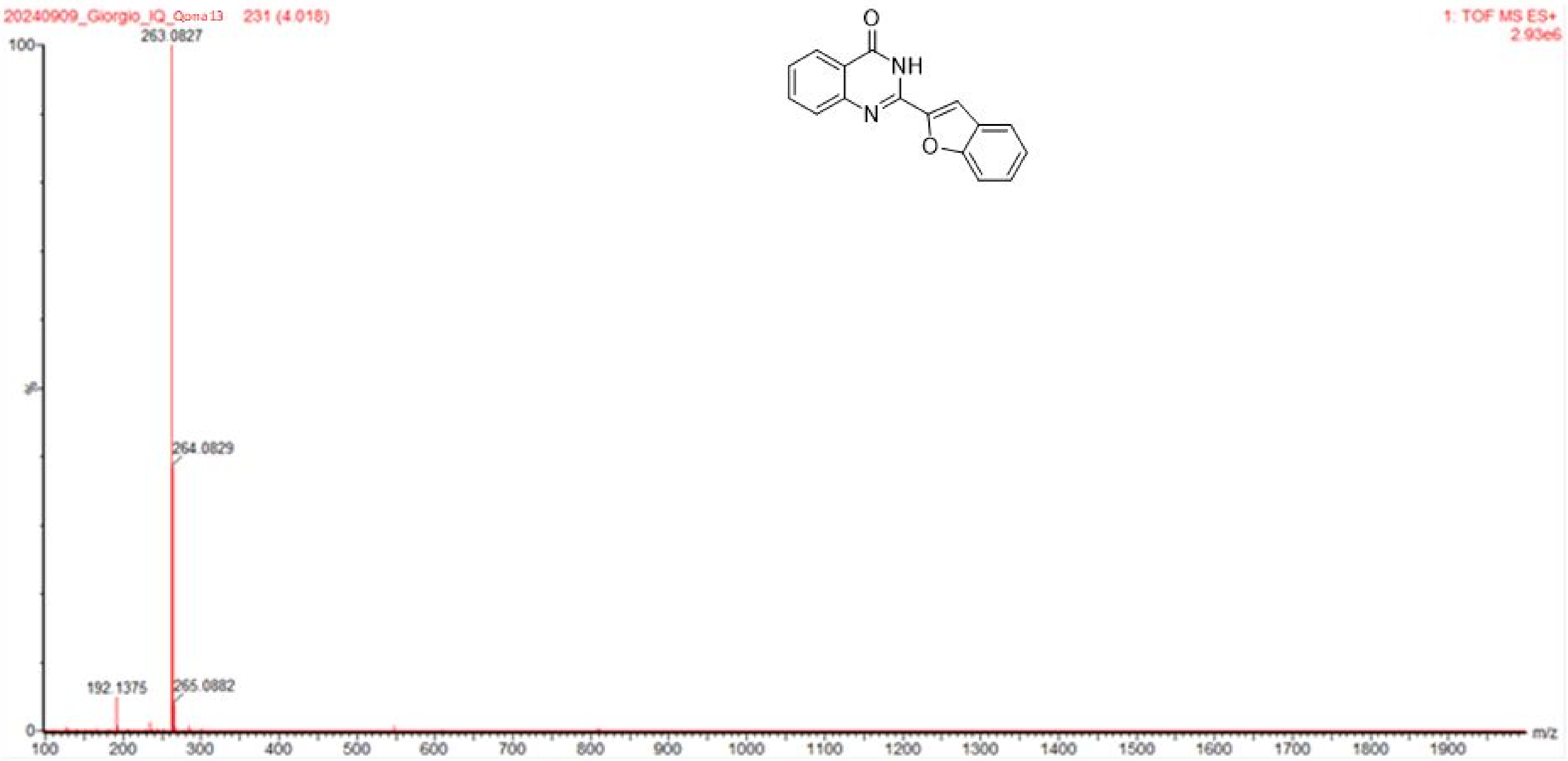

Figure 52S. MS of 2-(benzofuran-2-yl)quinazolin-4(3*H*)-one, **13**.

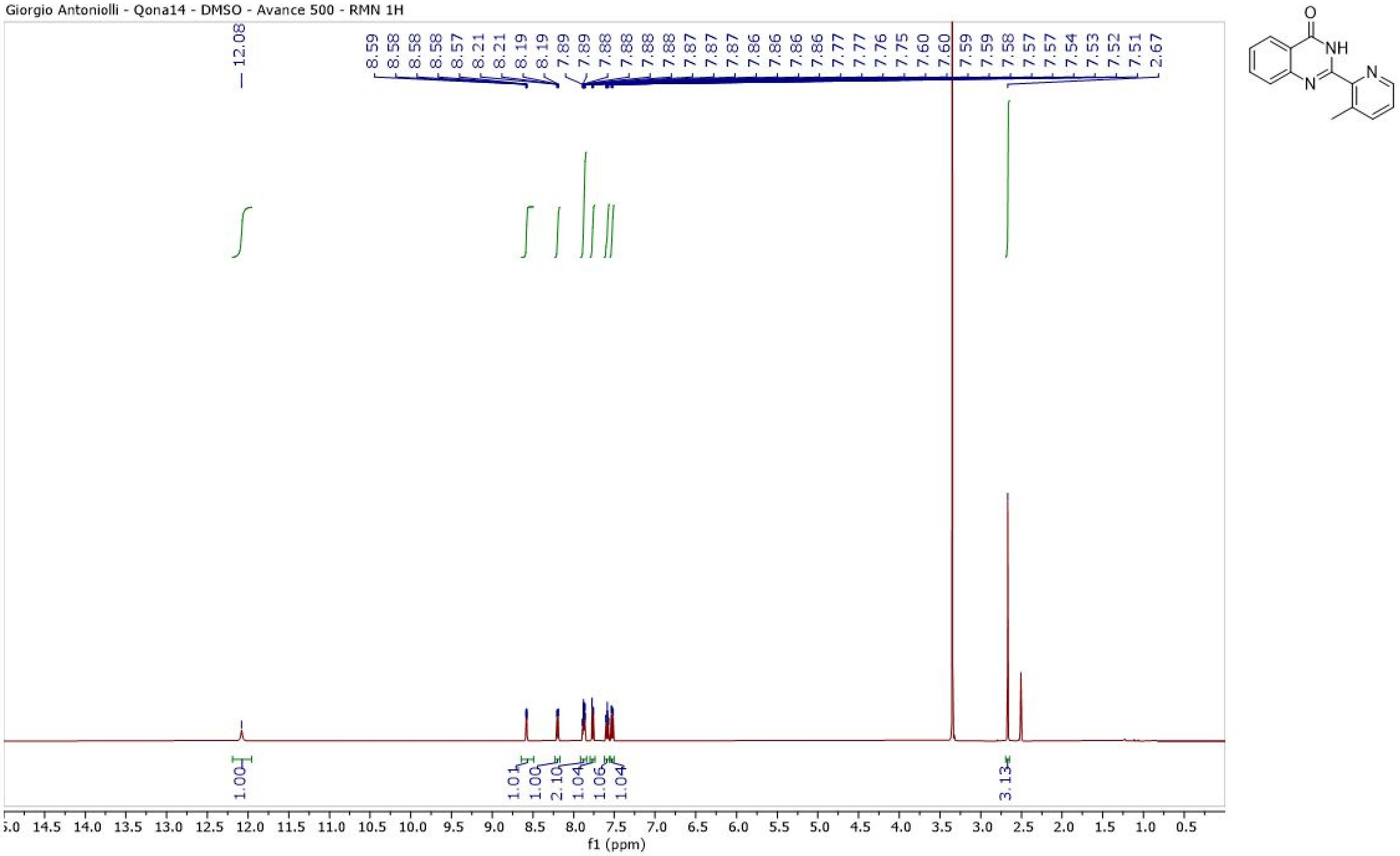

Figure 53S. ^{1}H NMR (d_6-DMSO, 500 MHz) of 2-(3-methylpyridin-2-yl)quinazolin-4(3*H*)-one, **14**.

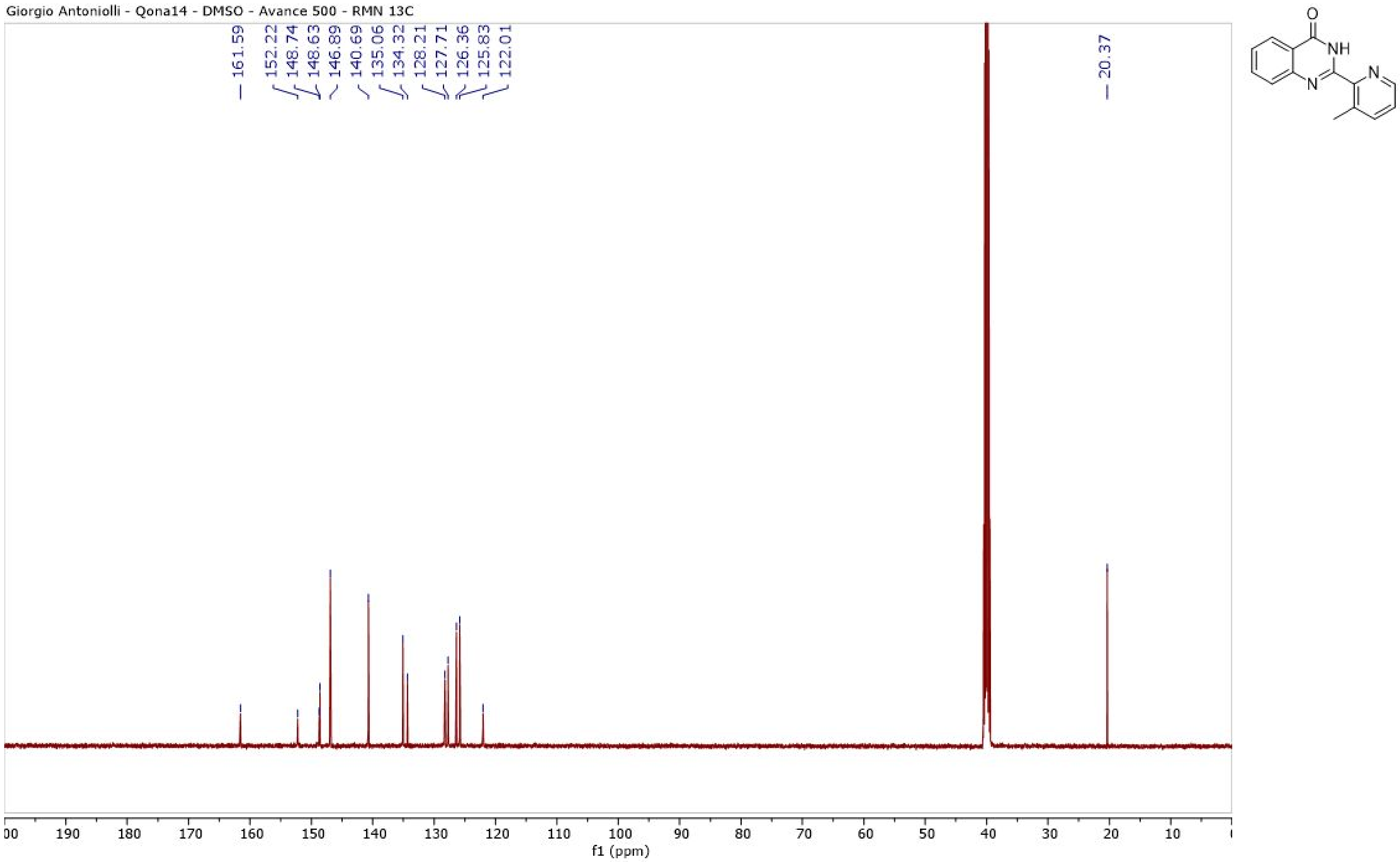

Figure 54S. ^{13}C NMR (d$_6$-DMSO, 125 MHz) of 2-(3-methylpyridin-2-yl)quinazolin-4(3*H*)-one, **14**.

Sample ID:	Qona14	Method Name:	PADRAO ATR
Sample Scans:	64	User:	Admin
Background Scans:	64	Date/Time:	17-Aug-23 3:13:47PM
Resolution:	4 cm-1	Range:	4,000.00 - 650.00
System Status:	Good	Apodization:	Happ-Genzel
File Location:	C:\Program Files\Agilent\MicroLab PC\Results\Qona14_2023-08-17T15-14-41.a2r		

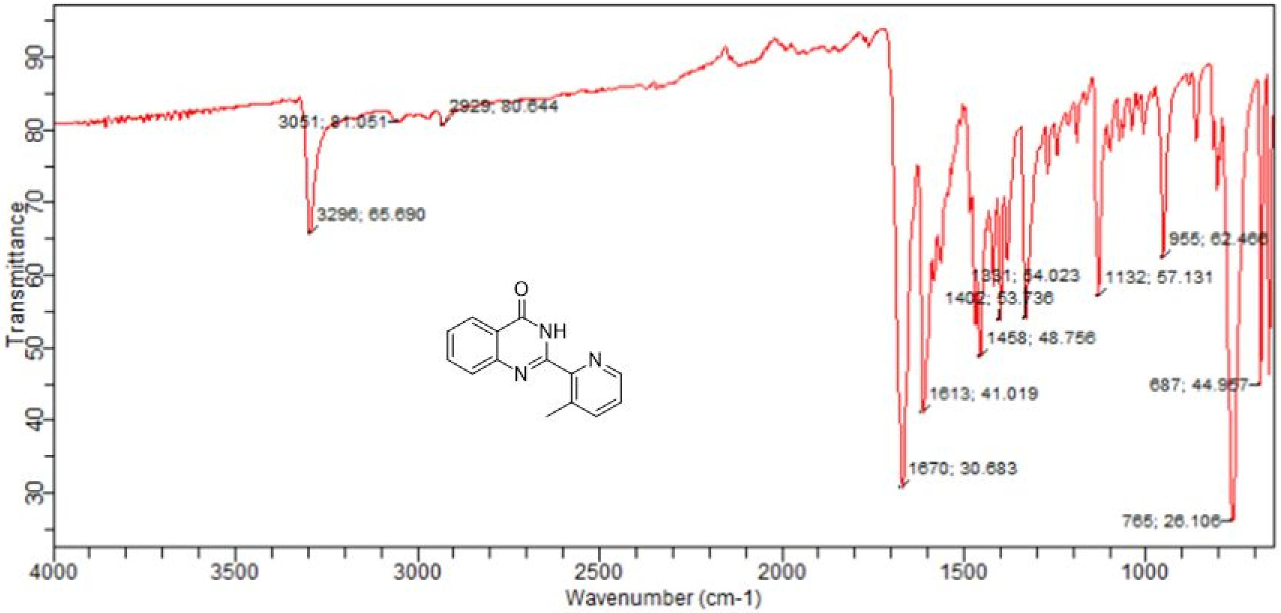

Figure 55S. IR (ATR, ν_{max}, cm^{-1}) of 2-(3-methylpyridin-2-yl)quinazolin-4(3*H*)-one, **14**.

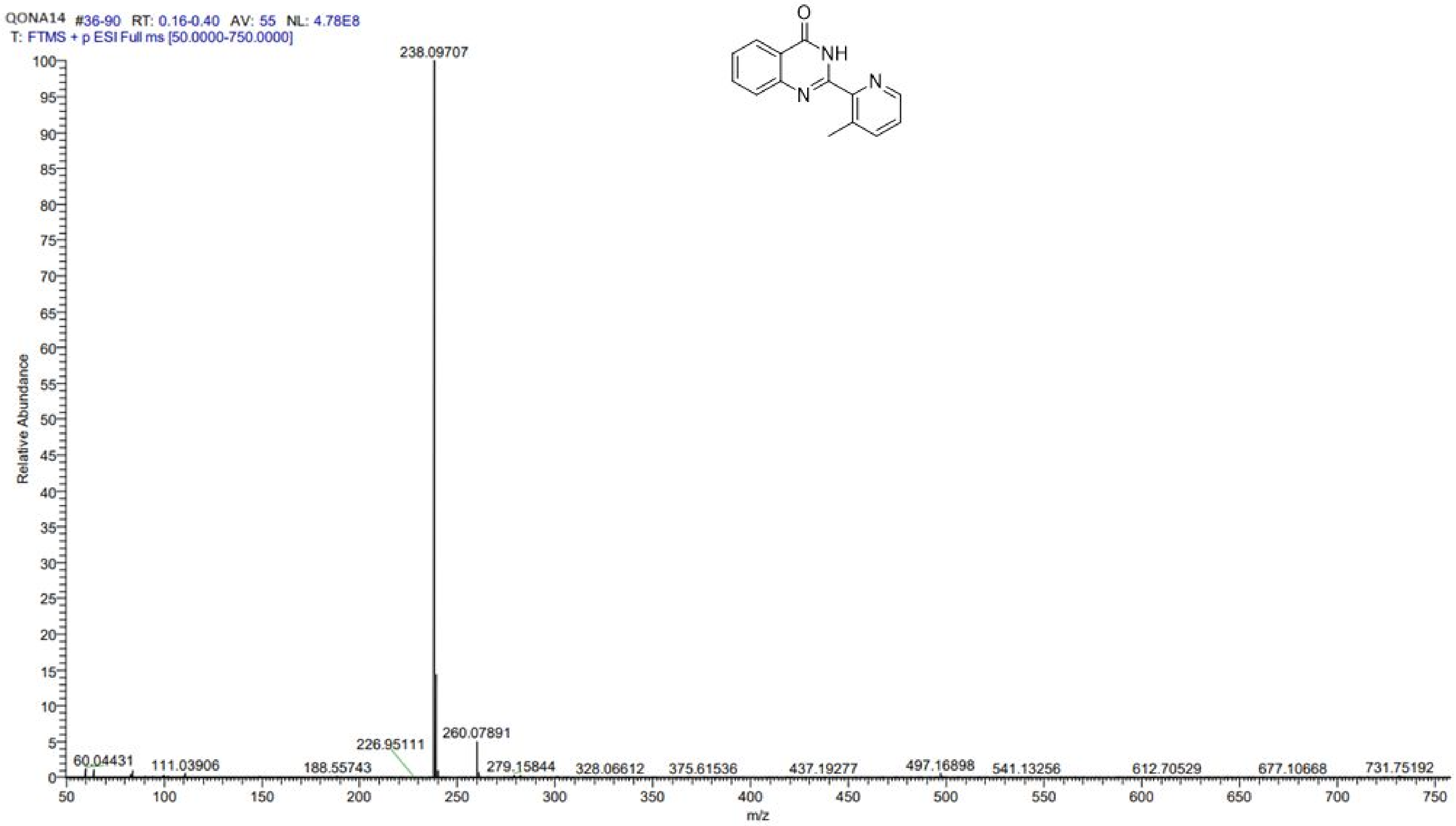

Figure 56S. MS of 2-(3-methylpyridin-2-yl)quinazolin-4(3*H*)-one, **14**.

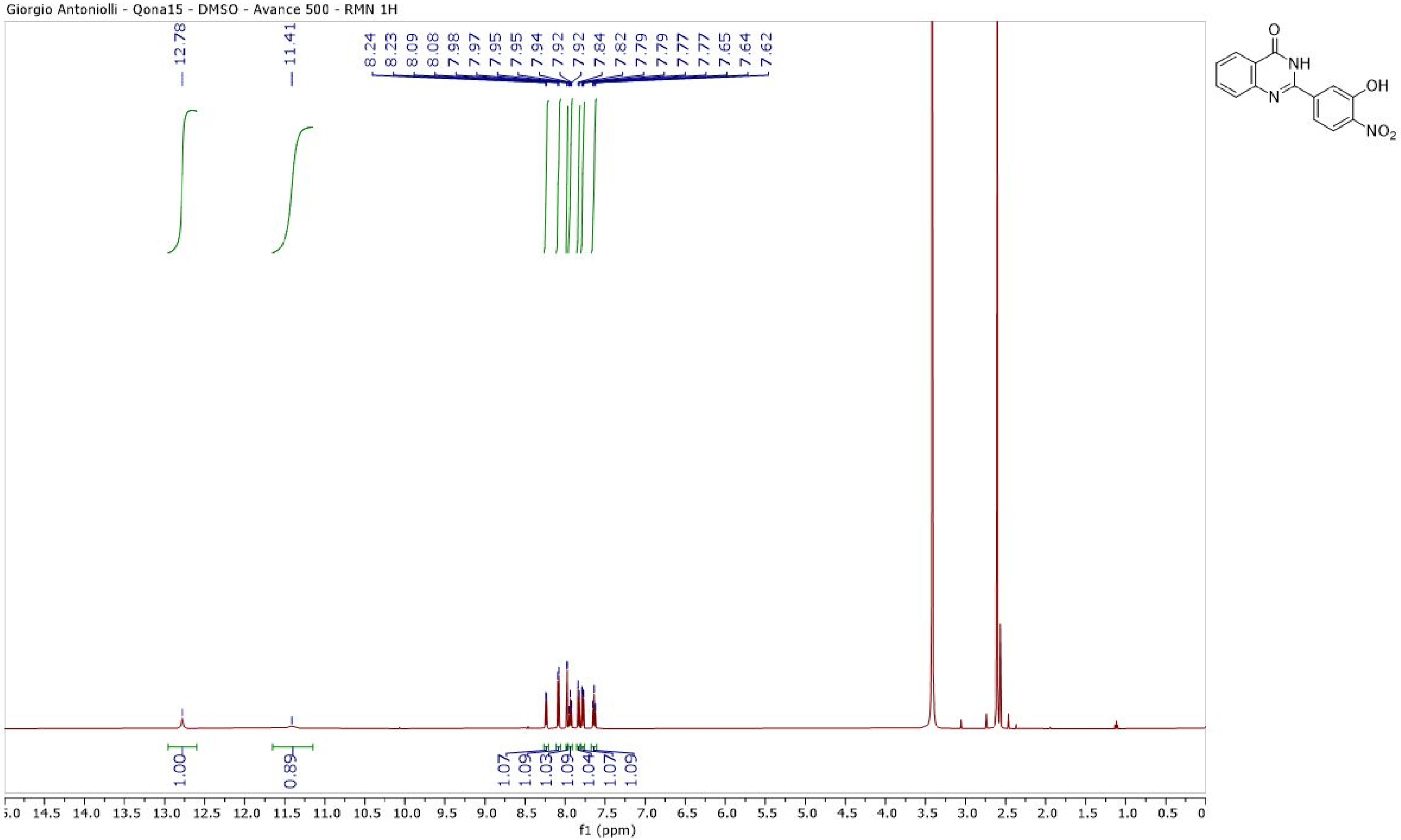

Figure 57S. ^{1}H NMR (d$_6$-DMSO, 500 MHz) of 2-(3-hydroxy-4-nitrophenyl)quinazolin-4(3*H*)-one, **15**.

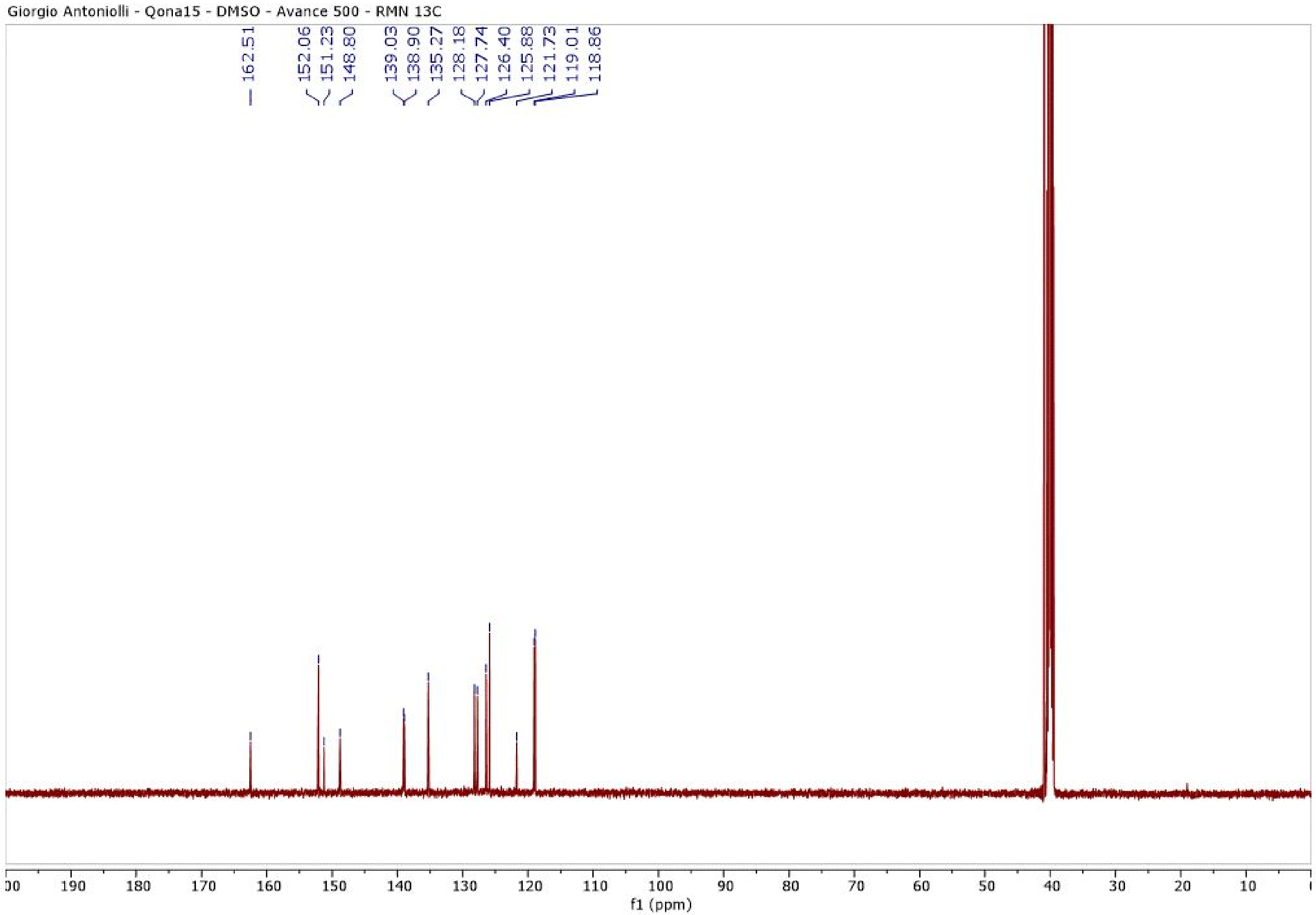

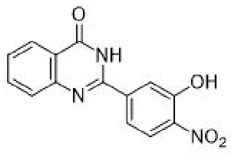

Figure 58S. ^{13}C NMR (d_6-DMSO, 125 MHz) of 2-(3-hydroxy-4-nitrophenyl)quinazolin-4(3*H*)-one, **15**.

Sample ID: Qona15

Sample Scans: 128

Background Scans: 128

Resolution: 2 cm-1

System Status: Good

File Location: C:\Program Files\Agilent\MicroLab PC\Results\Qona15_2024-06-28T12-52-46.a2r

Method Name: PADRAO ATR

User: Admin

Date/Time: 28-Jun-24 12:50:01PM

Range: 4,000.00 - 400.00

Apodization: Happ-Genzel

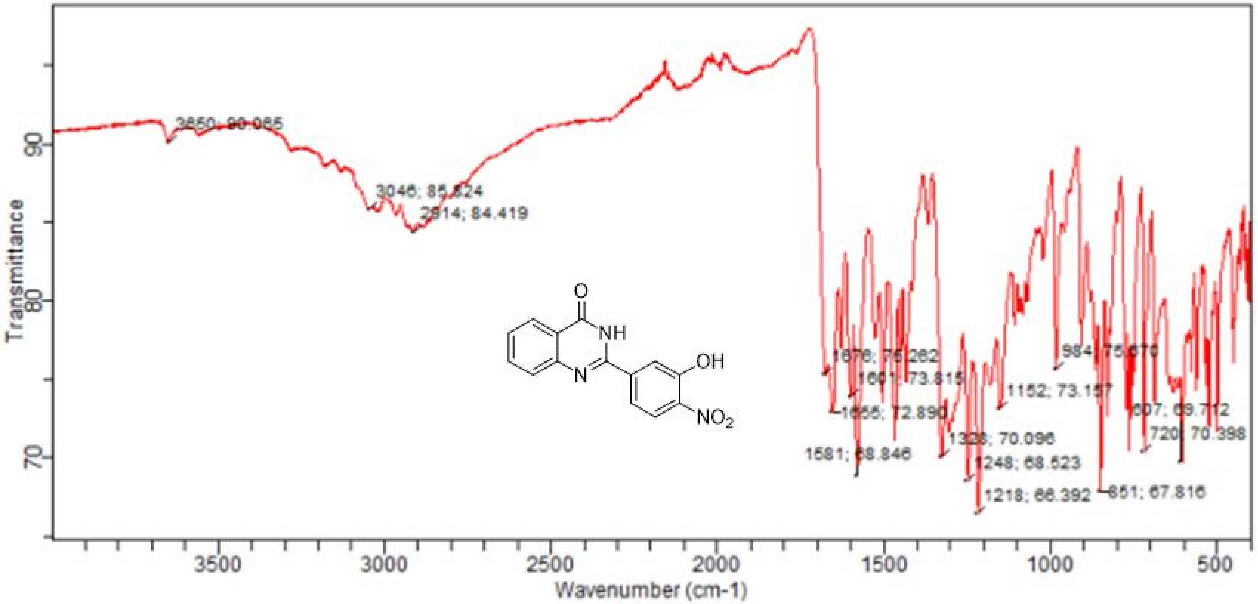

Figure 59S. IR (ATR, ν_{max}, cm^{-1}) of 2-(3-hydroxy-4-nitrophenyl)quinazolin-4(3*H*)-one, **15**.

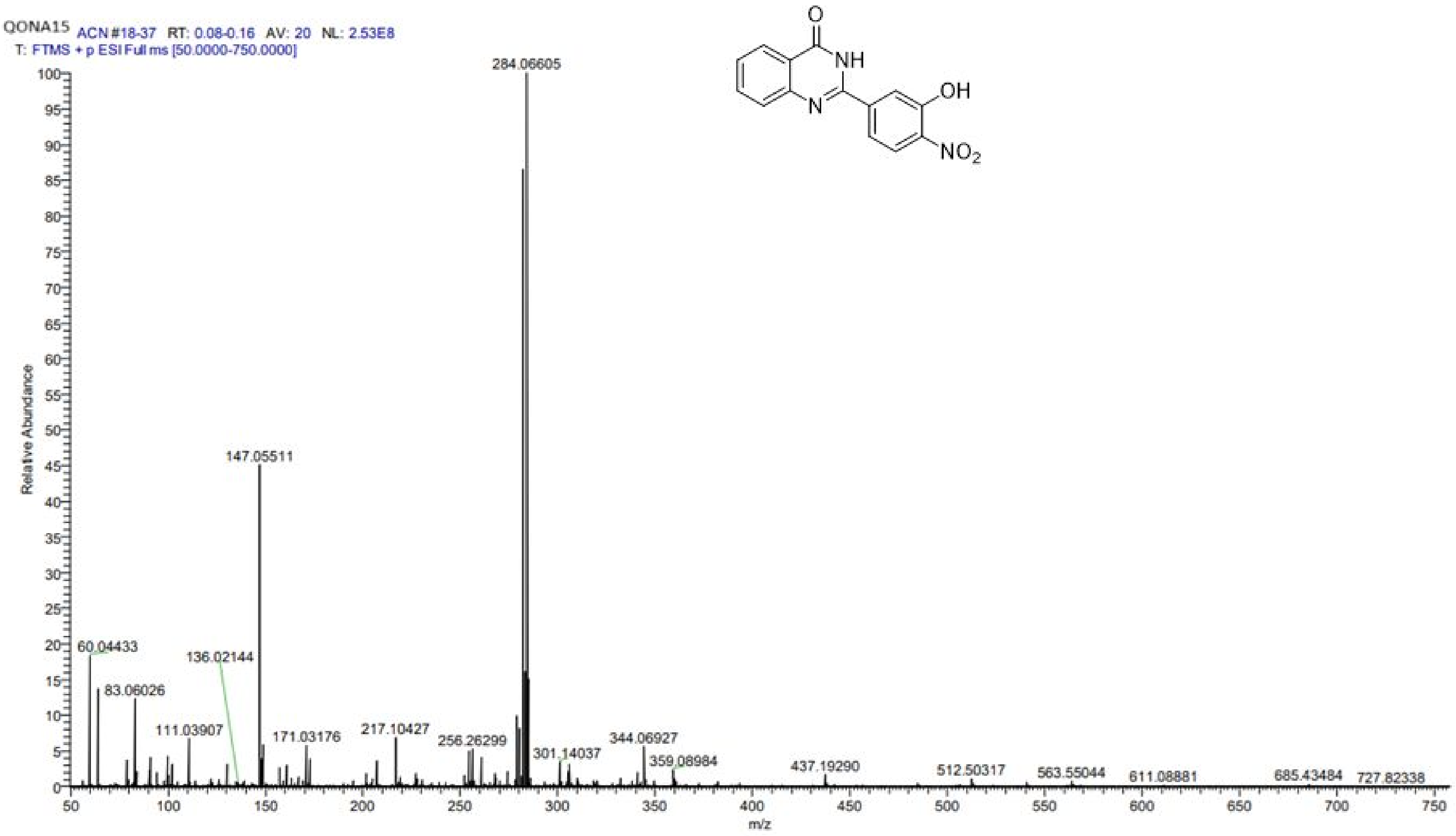

Figure 60S. MS of 2-(3-hydroxy-4-nitrophenyl)quinazolin-4(3*H*)-one, **15**.

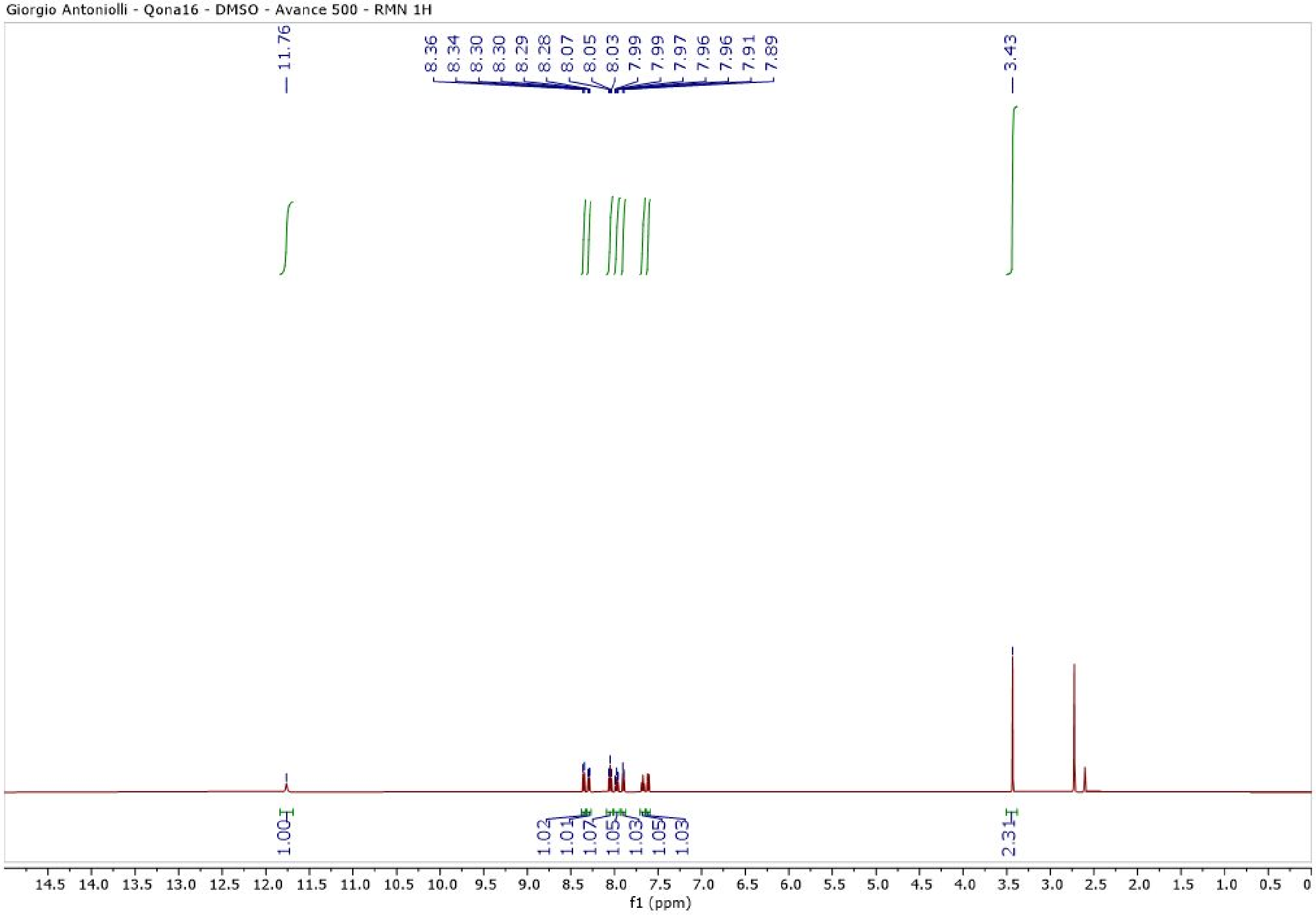

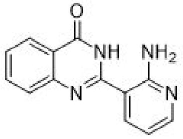

Figure 61S. ^{1}H NMR (d_6-DMSO, 500 MHz) of 2-(2-aminopyridin-3-yl)quinazolin-4(*3H*)-one, **16**.

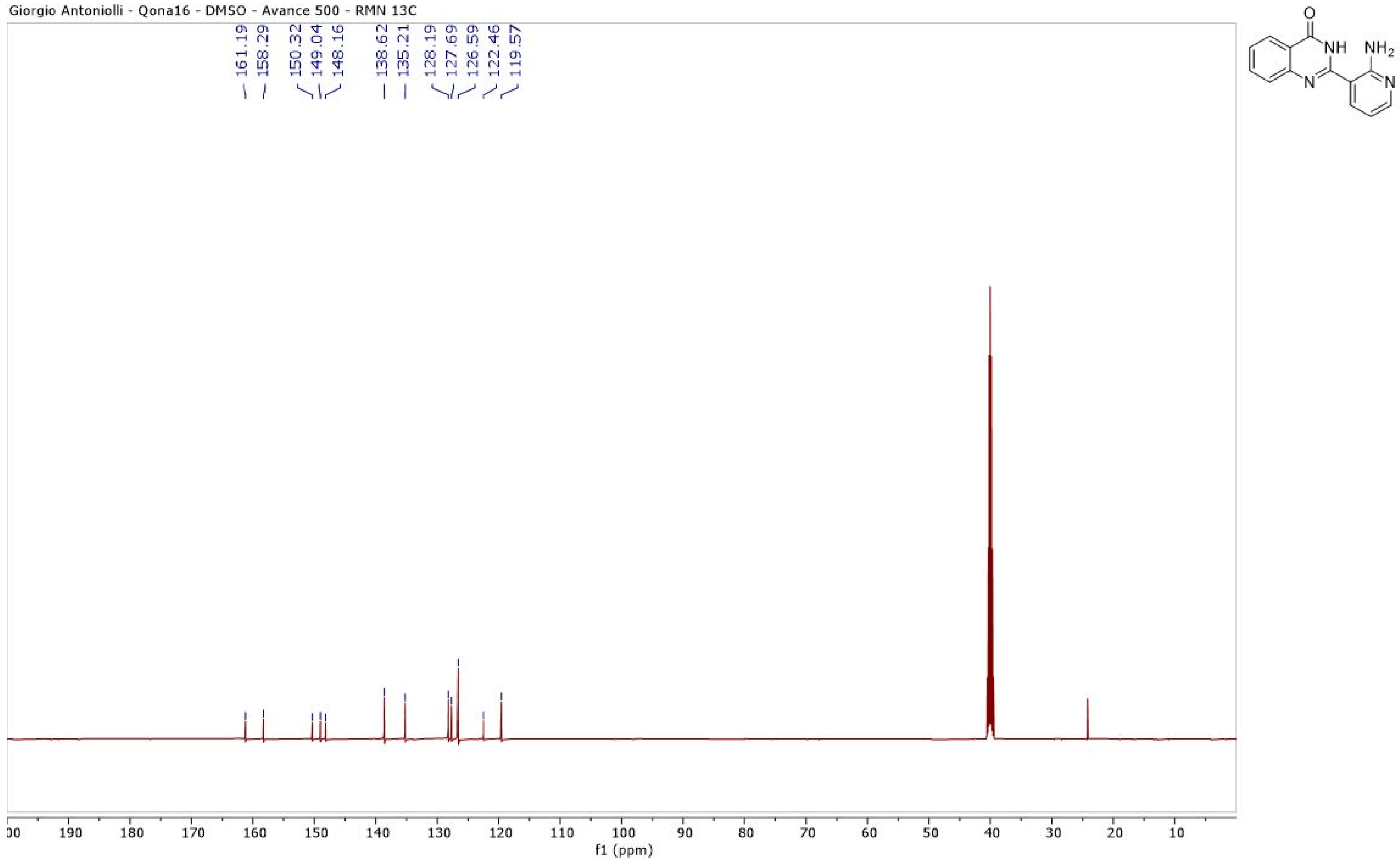

Figure 62S. ^{13}C NMR (d_6-DMSO, 125 MHz) of 2-(2-aminopyridin-3-yl)quinazolin-4(*3H*)-one, **16**.

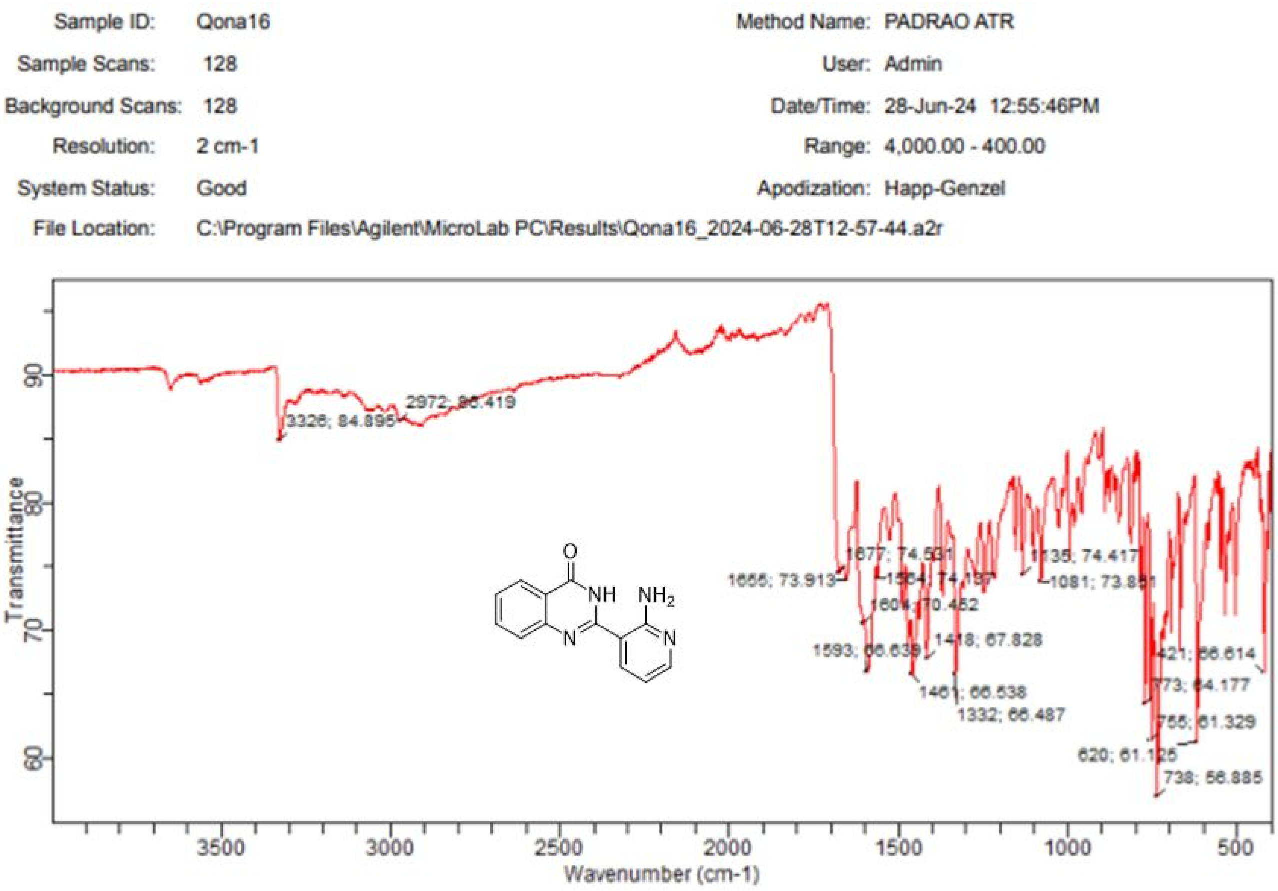

Figure 63S. IR (ATR, ν_{max}, cm^{-1}) of 2-(2-aminopyridin-3-yl)quinazolin-4(*3H*)-one, **16**.

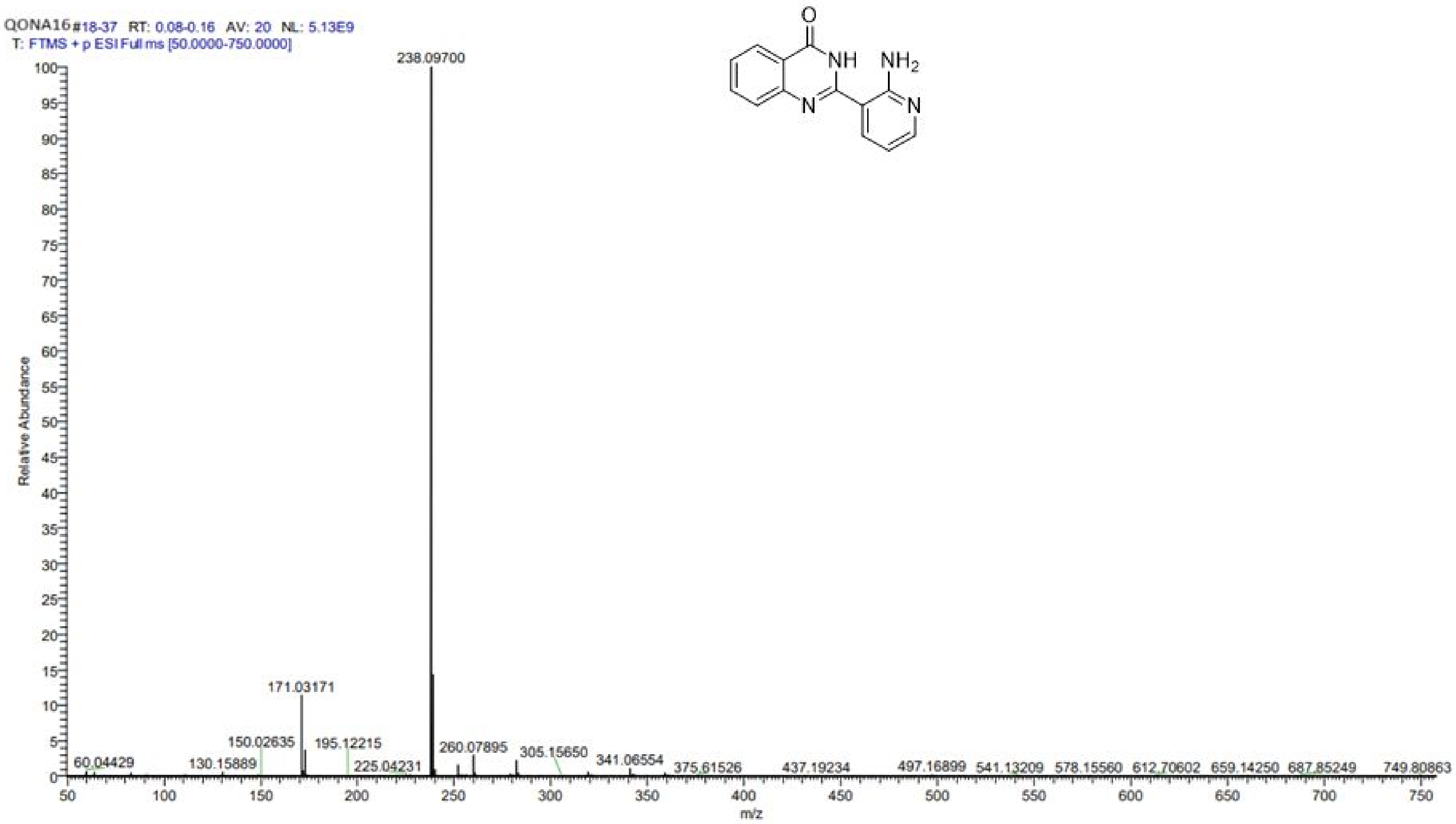

Figure 64S. MS of 2-(2-aminopyridin-3-yl)quinazolin-4(*3H*)-one, **16**.

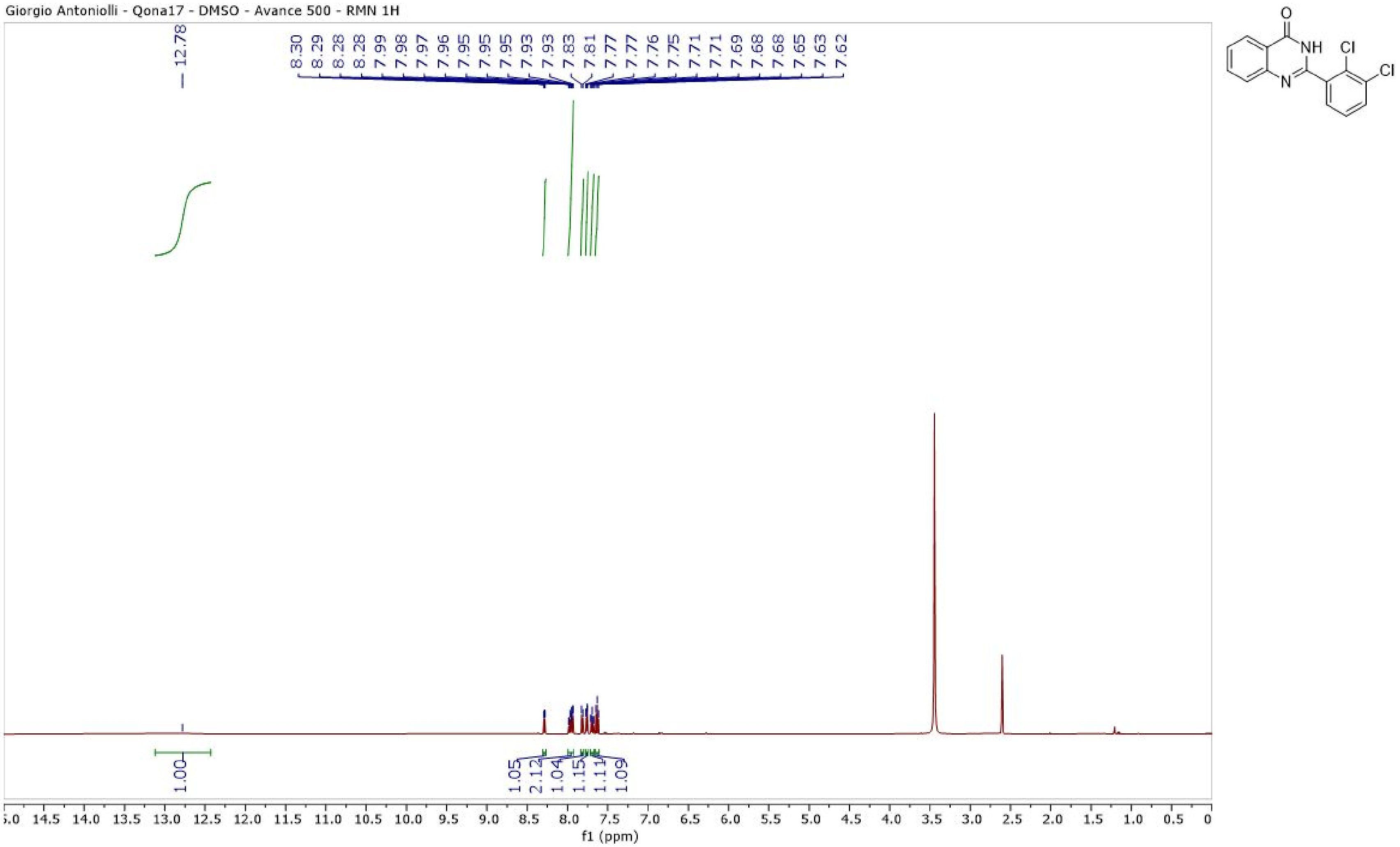

Figure 65S. ^{1}H NMR (d_6-DMSO, 500 MHz) of 2-(2,3-dichlorophenyl)quinazolin-4(3*H*)-one, **17**.

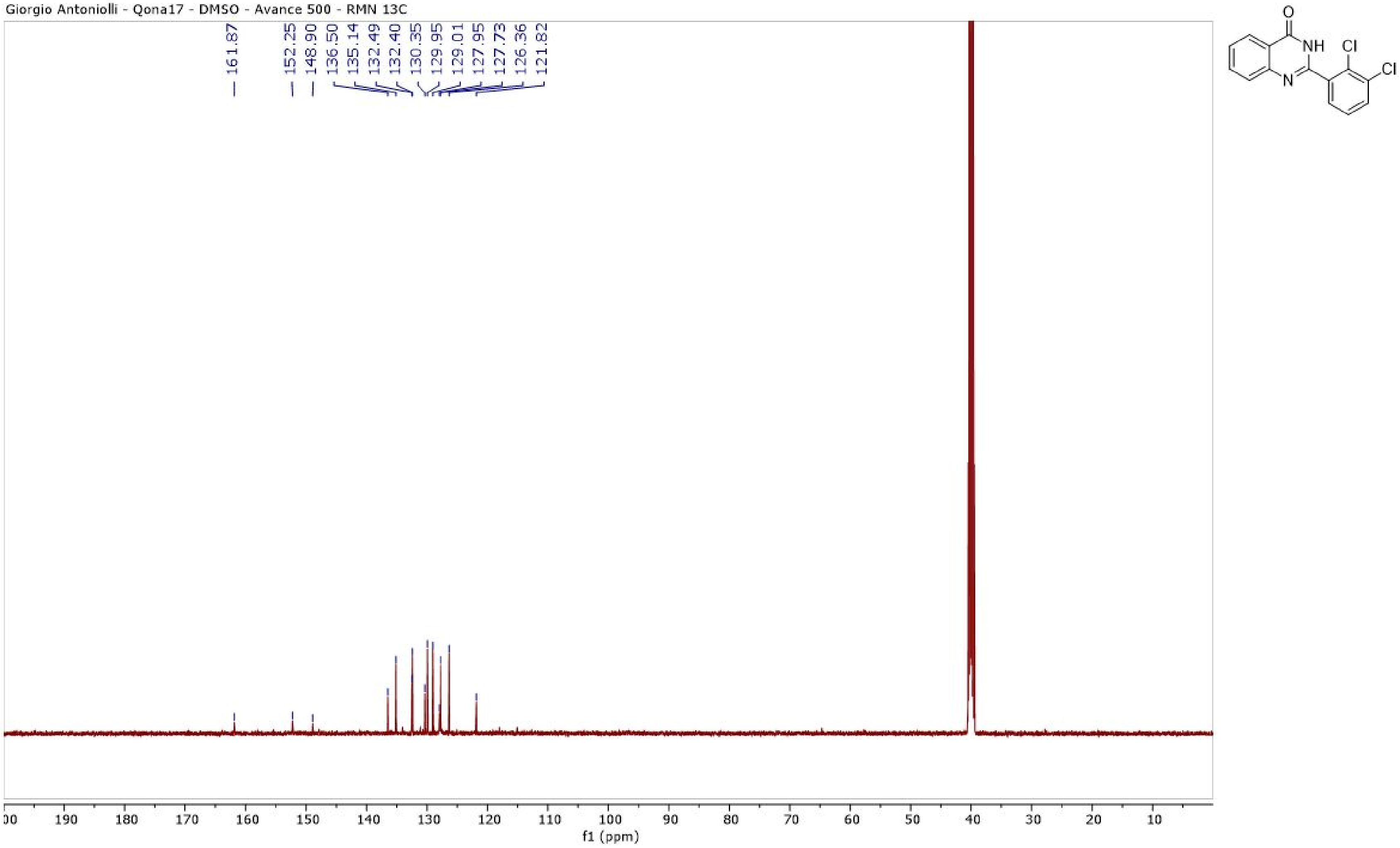

Figure 66S. ^{13}C NMR (d$_6$-DMSO, 125 MHz) of 2-(2,3-dichlorophenyl)quinazolin-4(3*H*)-one, **17**.

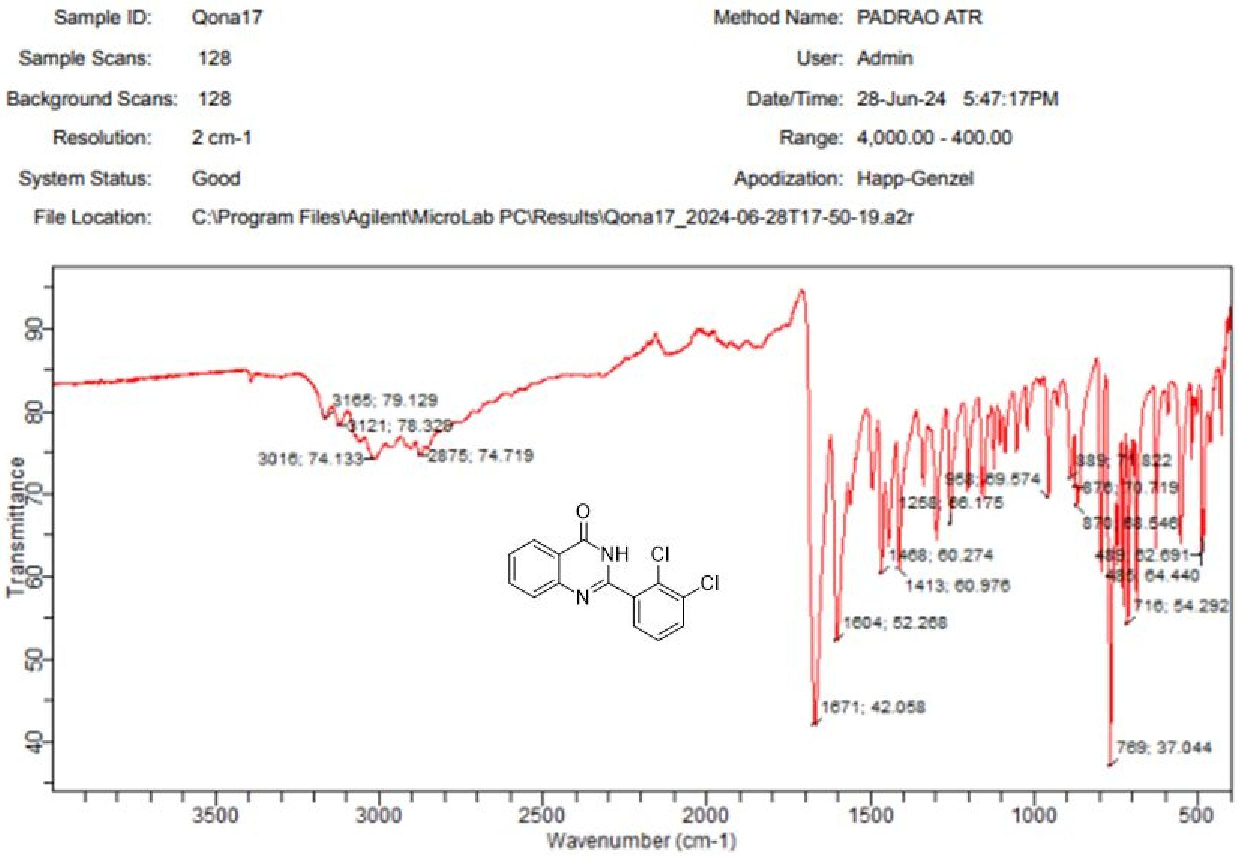

Figure 67S. IR (ATR, ν_{max}, cm^{-1}) of 2-(2,3-dichlorophenyl)quinazolin-4(3*H*)-one, **17**.

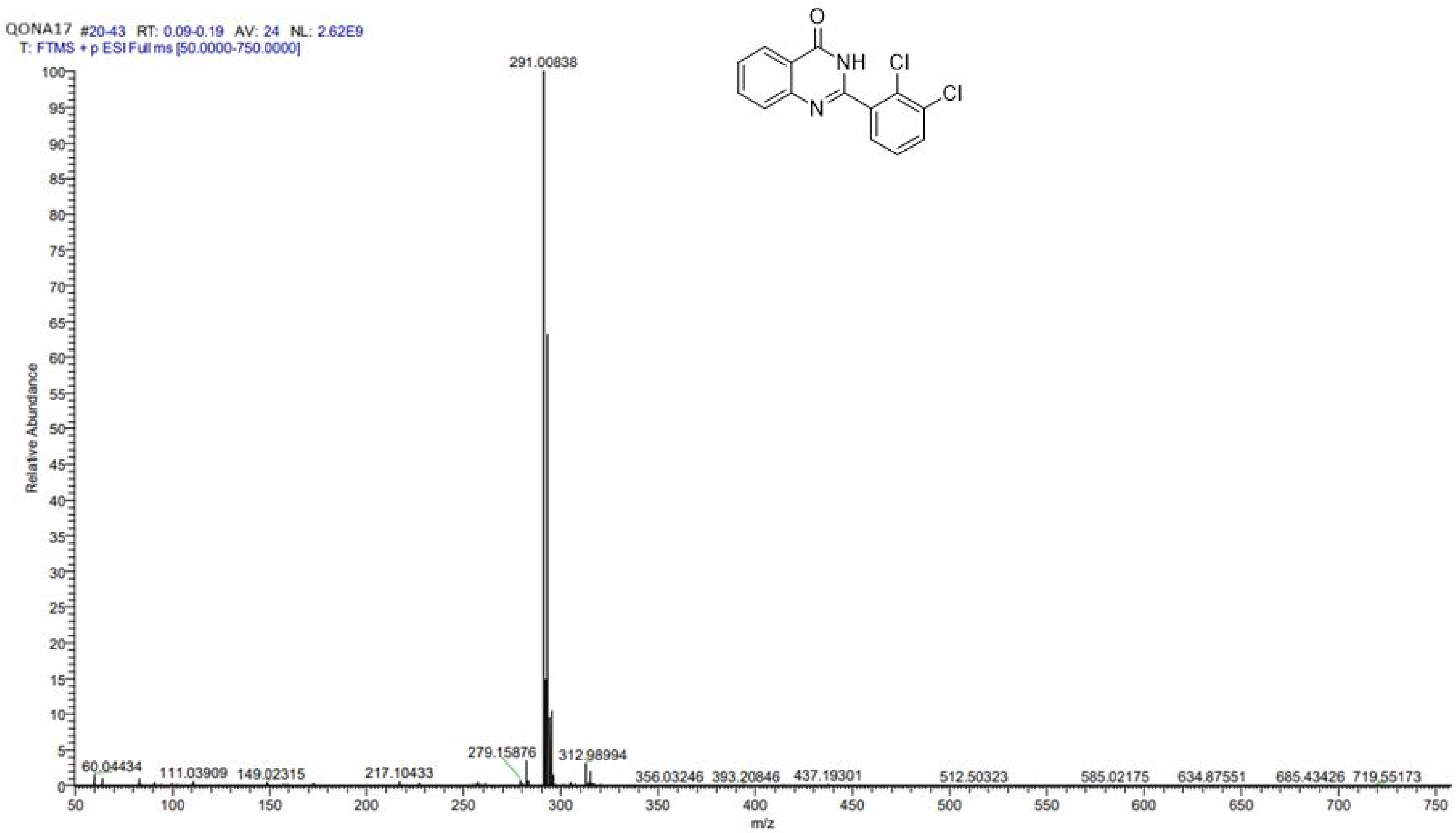

Figure 68S. MS of 2-(2,3-dichlorophenyl)quinazolin-4(3*H*)-one, **17**.

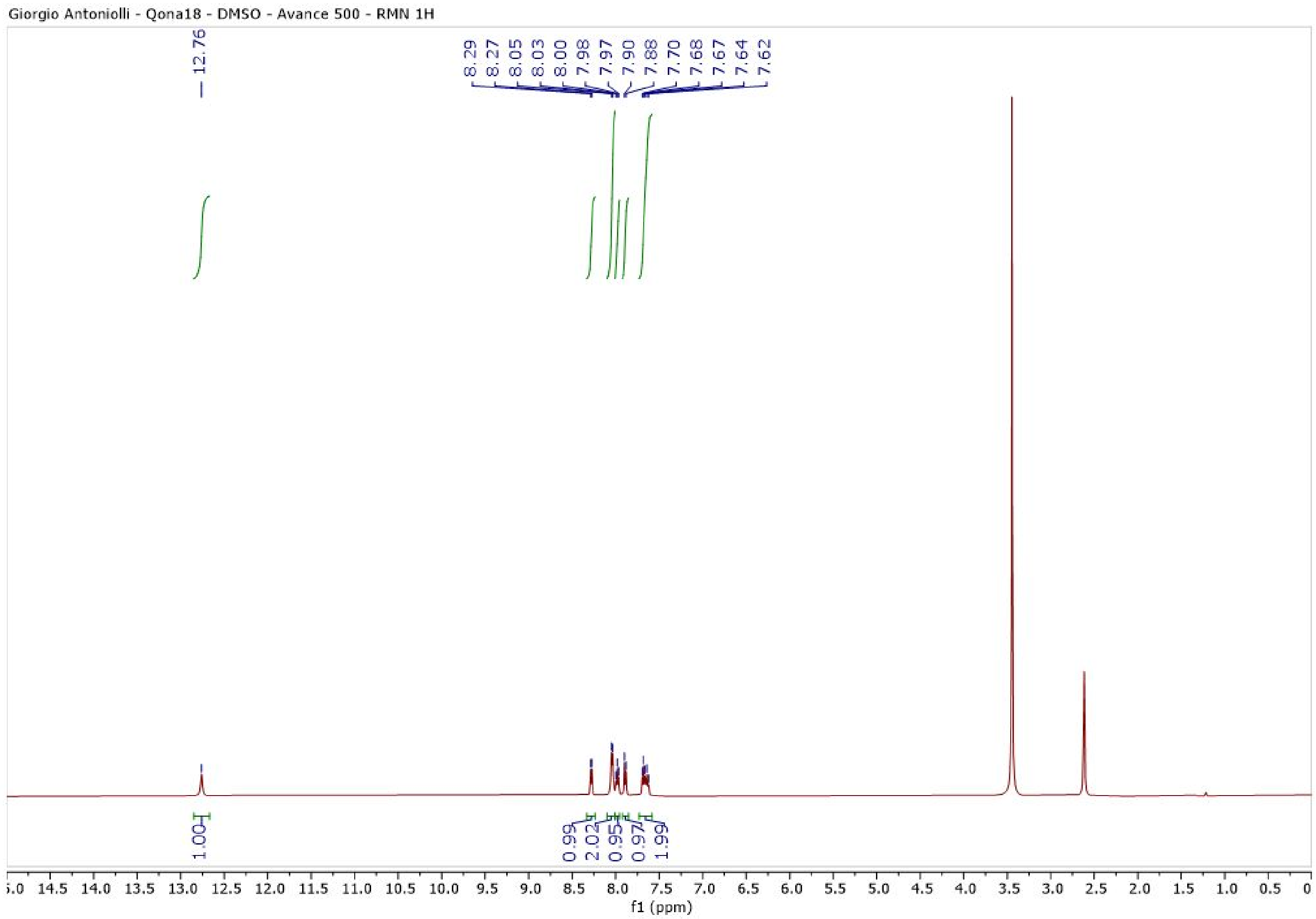

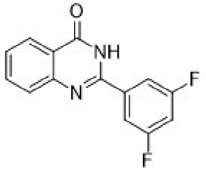

Figure 69S. ^{1}H NMR (d_6-DMSO, 500 MHz) of 2-(3,5-difluorophenyl)quinazolin-4(3*H*)-one, **18**.

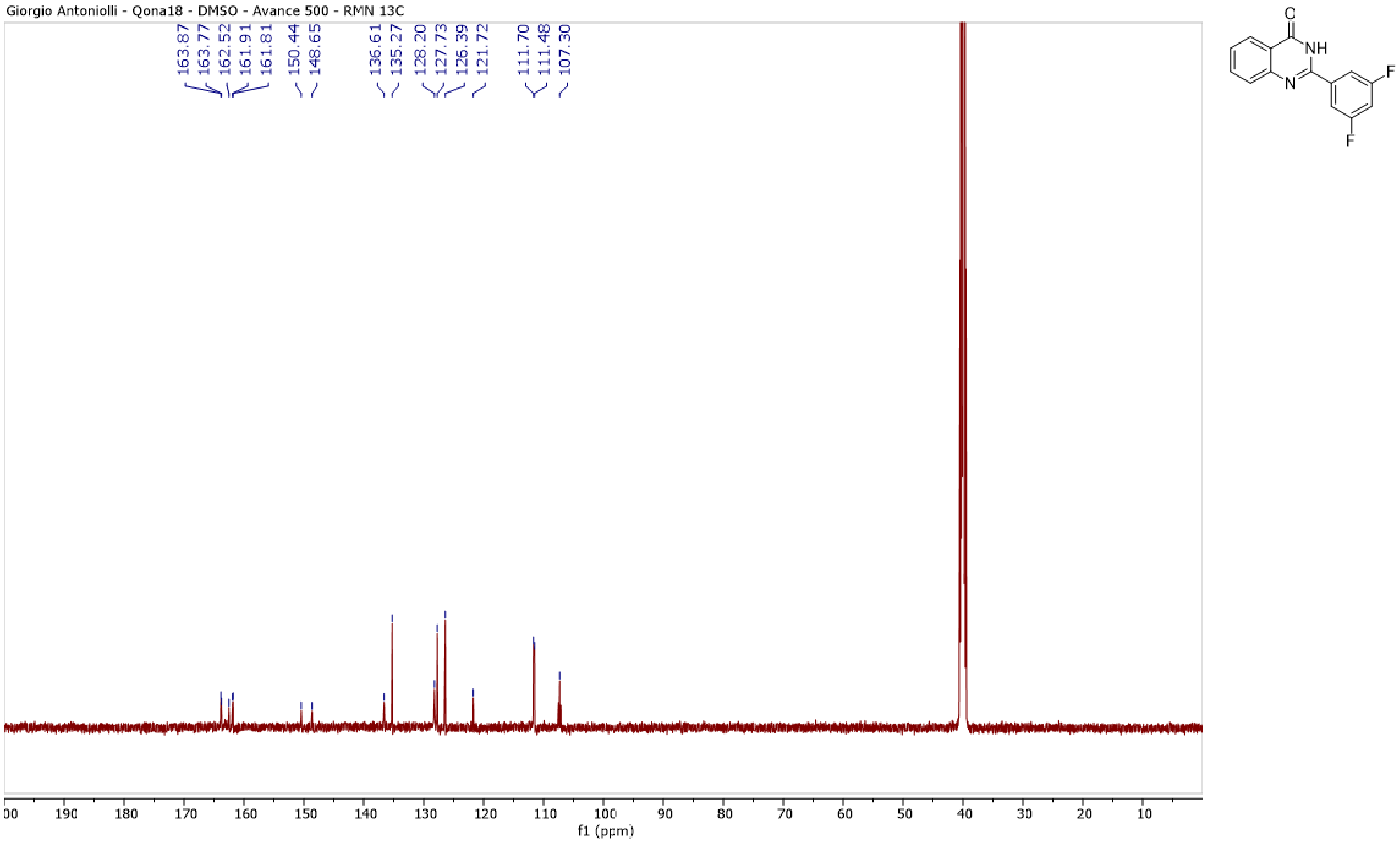

Figure 70S. ^{13}C NMR (d_6-DMSO, 125 MHz) of 2-(3,5-difluorophenyl)quinazolin-4(3*H*)-one, **18**.

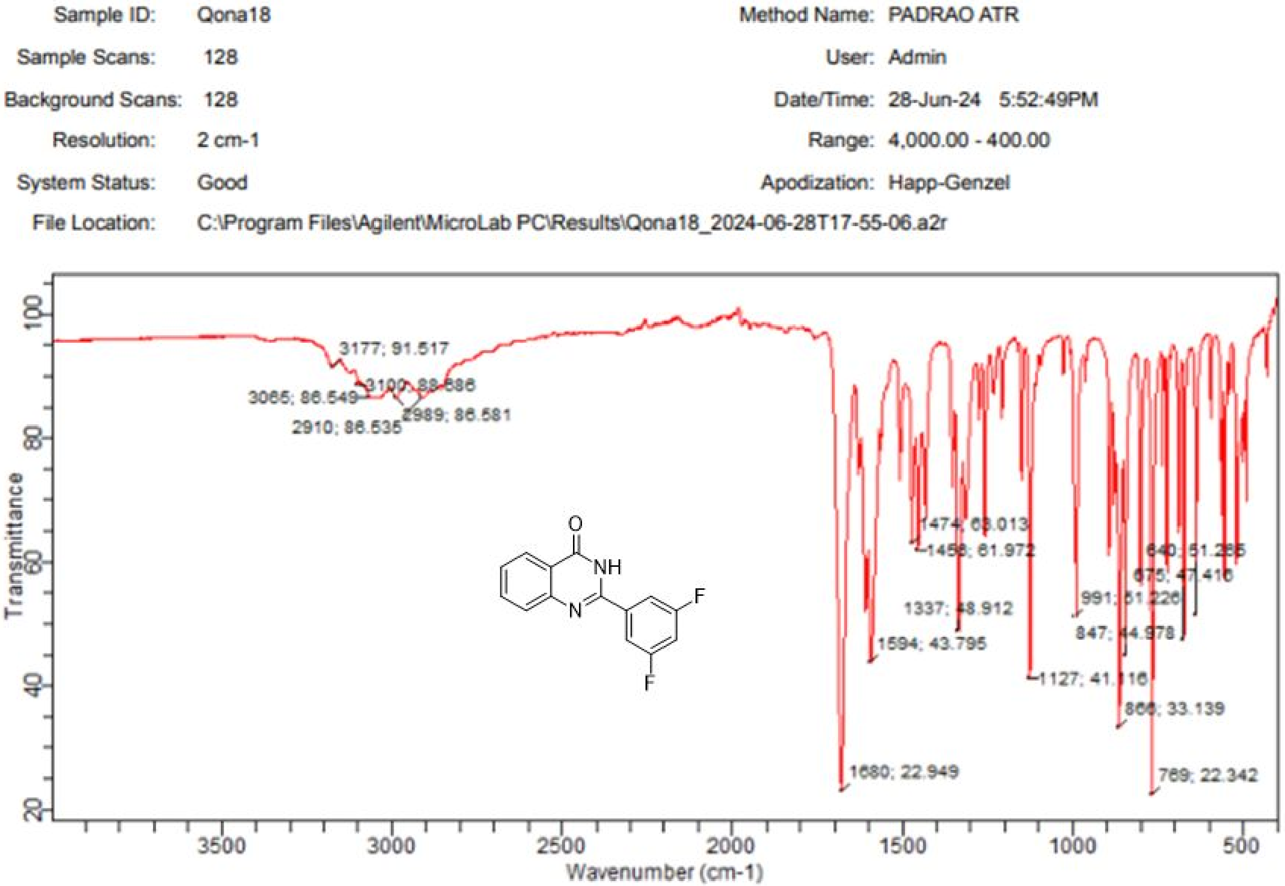

Figure 71S. IR (ATR, ν_{max}, cm^{-1}) of 2-(3,5-difluorophenyl)quinazolin-4(3*H*)-one, **18**.

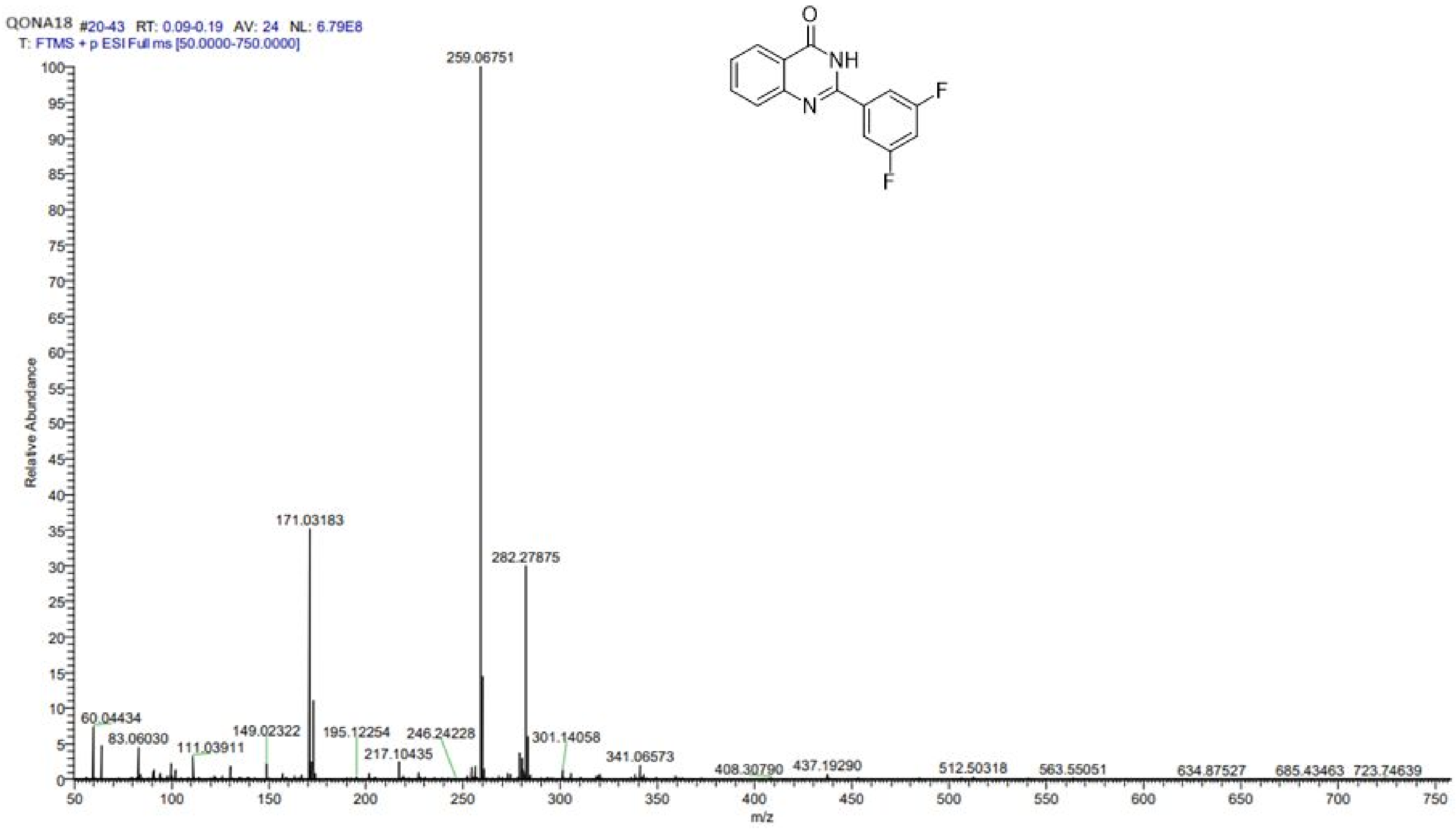

Figure 72S. MS of 2-(3,5-difluorophenyl)quinazolin-4(3*H*)-one, **18**.

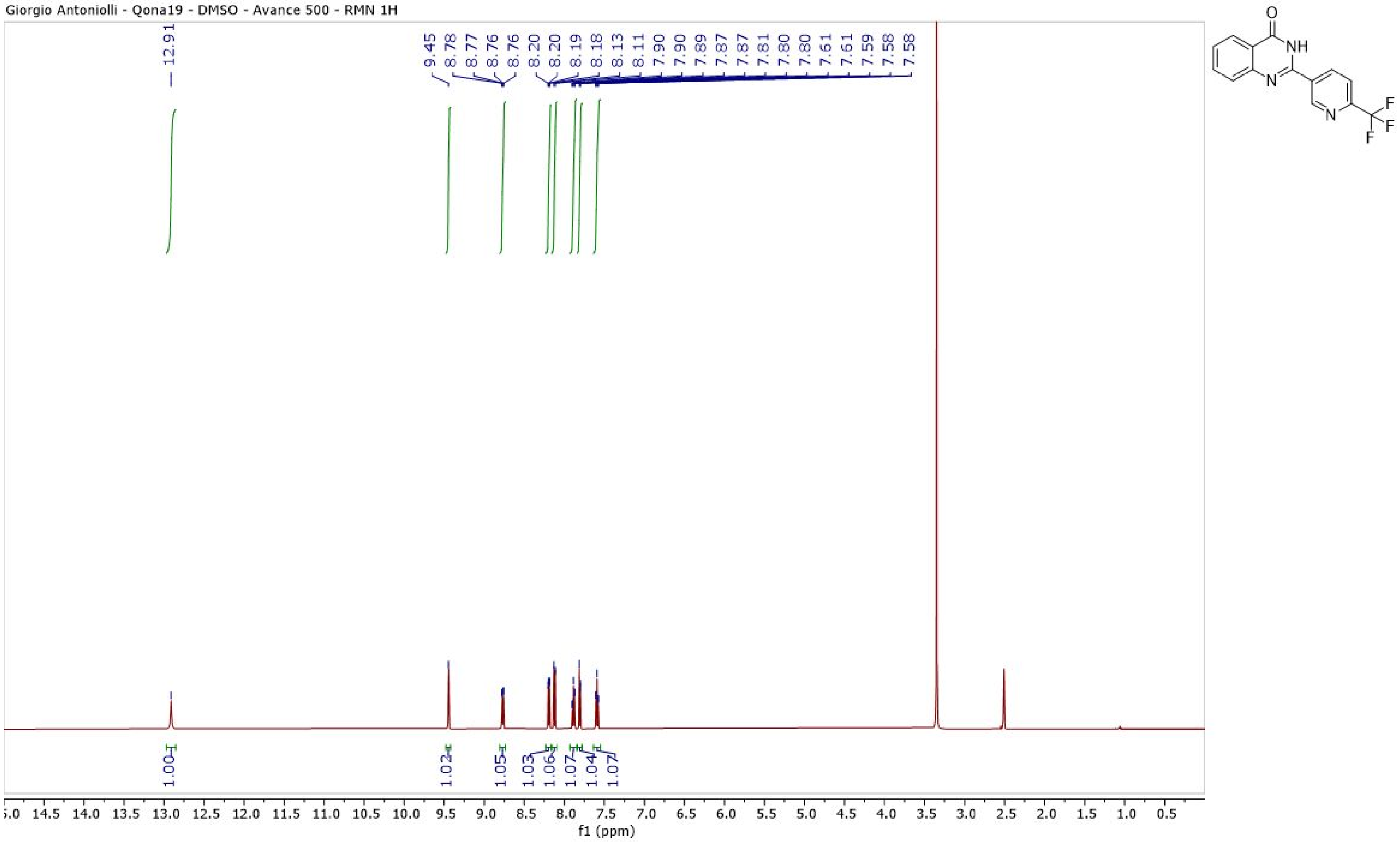

Figure 73S. ^{1}H NMR (d$_6$-DMSO, 500 MHz) of 2-(6-(trifluoromethyl)pyridin-3-yl)quinazolin-4(3*H*)-one, **19**.

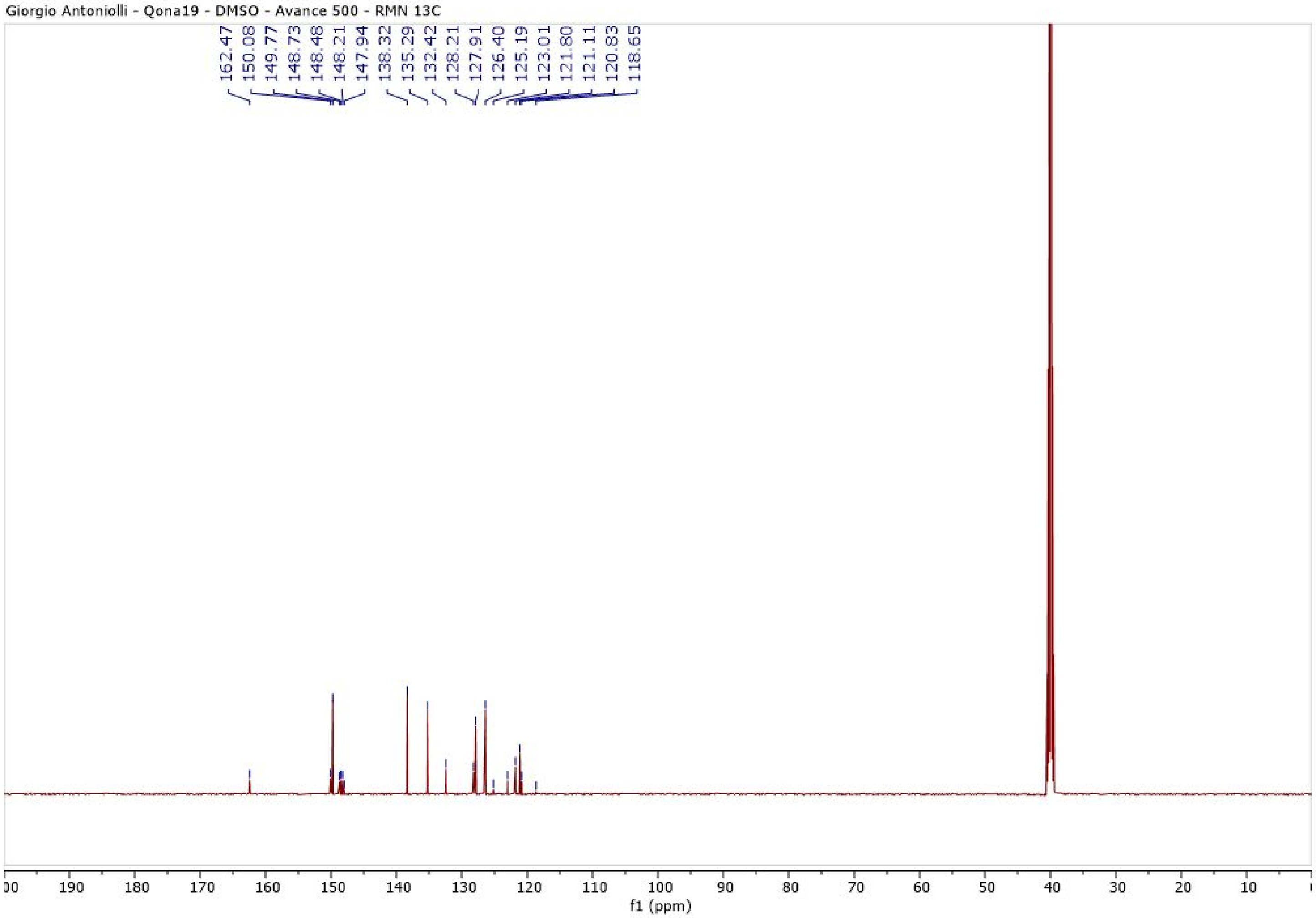

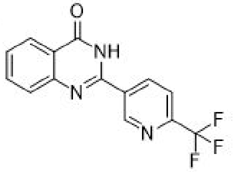

Figure 74S. ^{13}C NMR (d_6-DMSO, 125 MHz) of 2-(6-(trifluoromethyl)pyridin-3-yl)quinazolin-4(3*H*)-one, **19**.

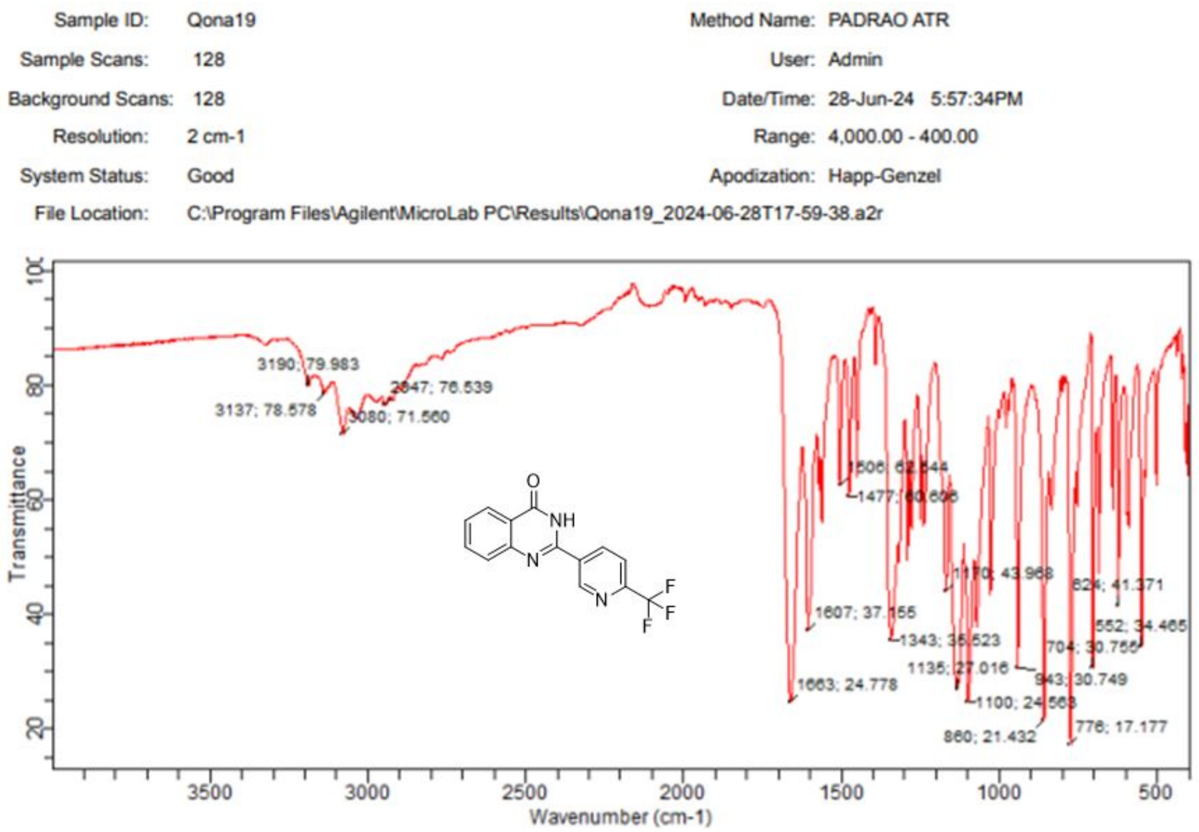

Figure 75S. IR (ATR, ν_{max}, cm^{-1}) of 2-(6-(trifluoromethyl)pyridin-3-yl)quinazolin-4(3*H*)-one, **19**.

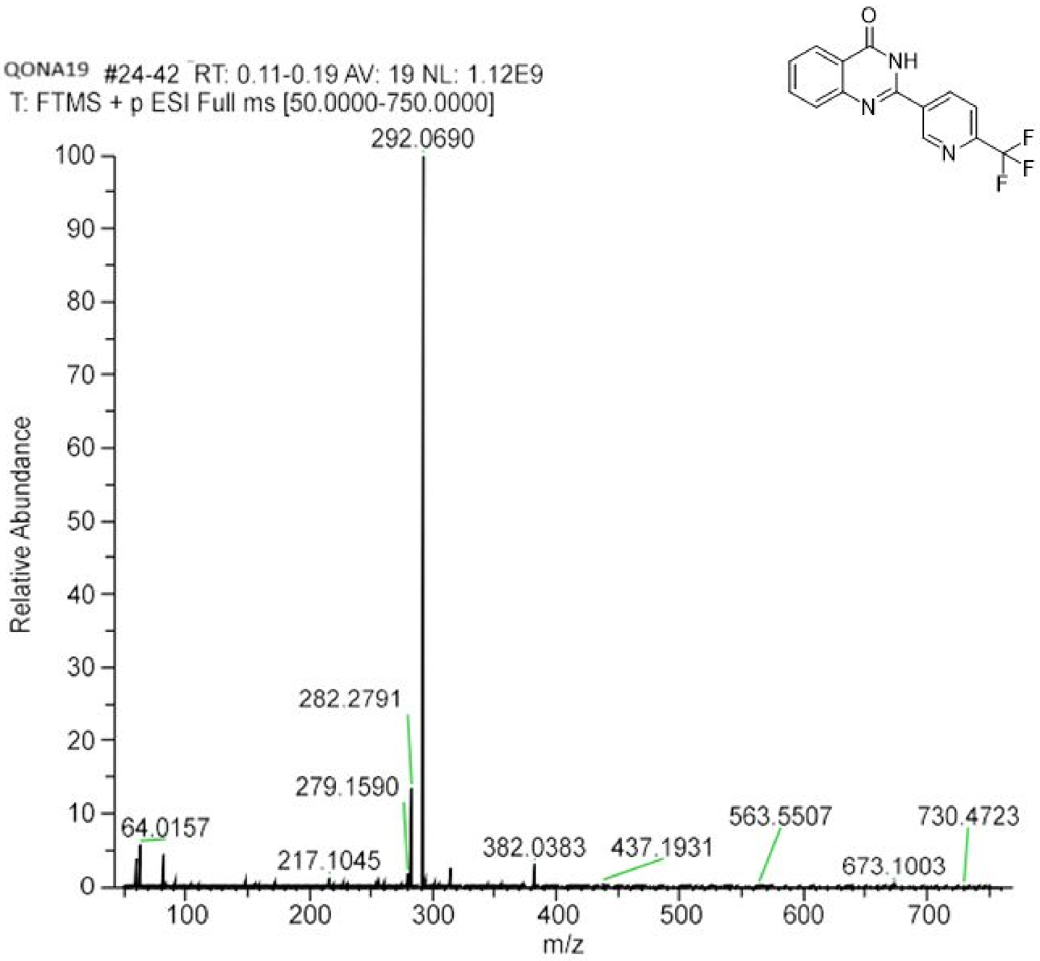

Figure 76S. MS of 2-(6-(trifluoromethyl)pyridin-3-yl)quinazolin-4(3*H*)-one, **19**.

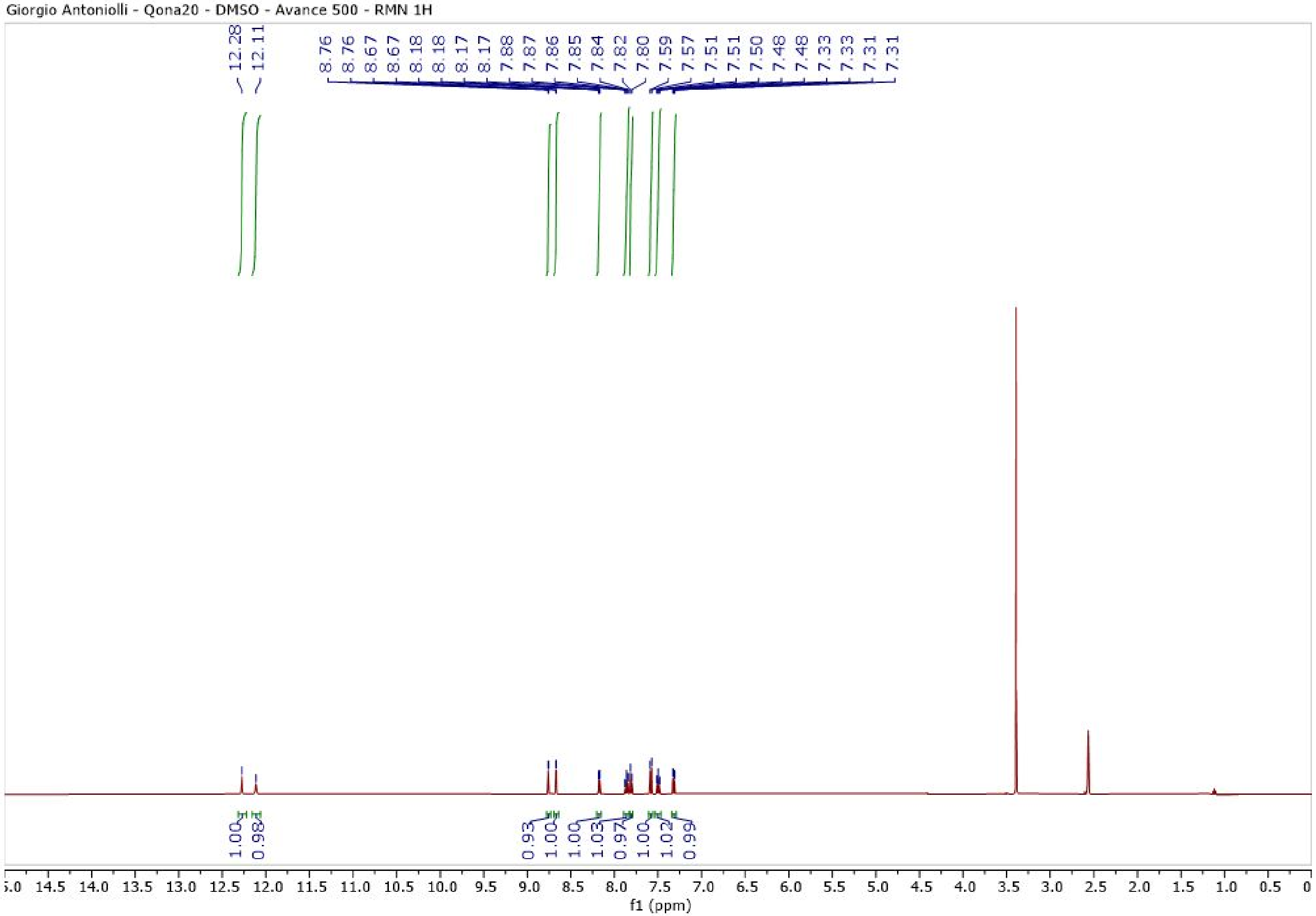

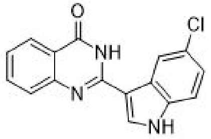

Figure 77S. ^{1}H NMR (d_6-DMSO, 500 MHz) of 2-(5-chloro-1*H*-indol-3-yl)quinazolin-4(3*H*)-one, **20**.

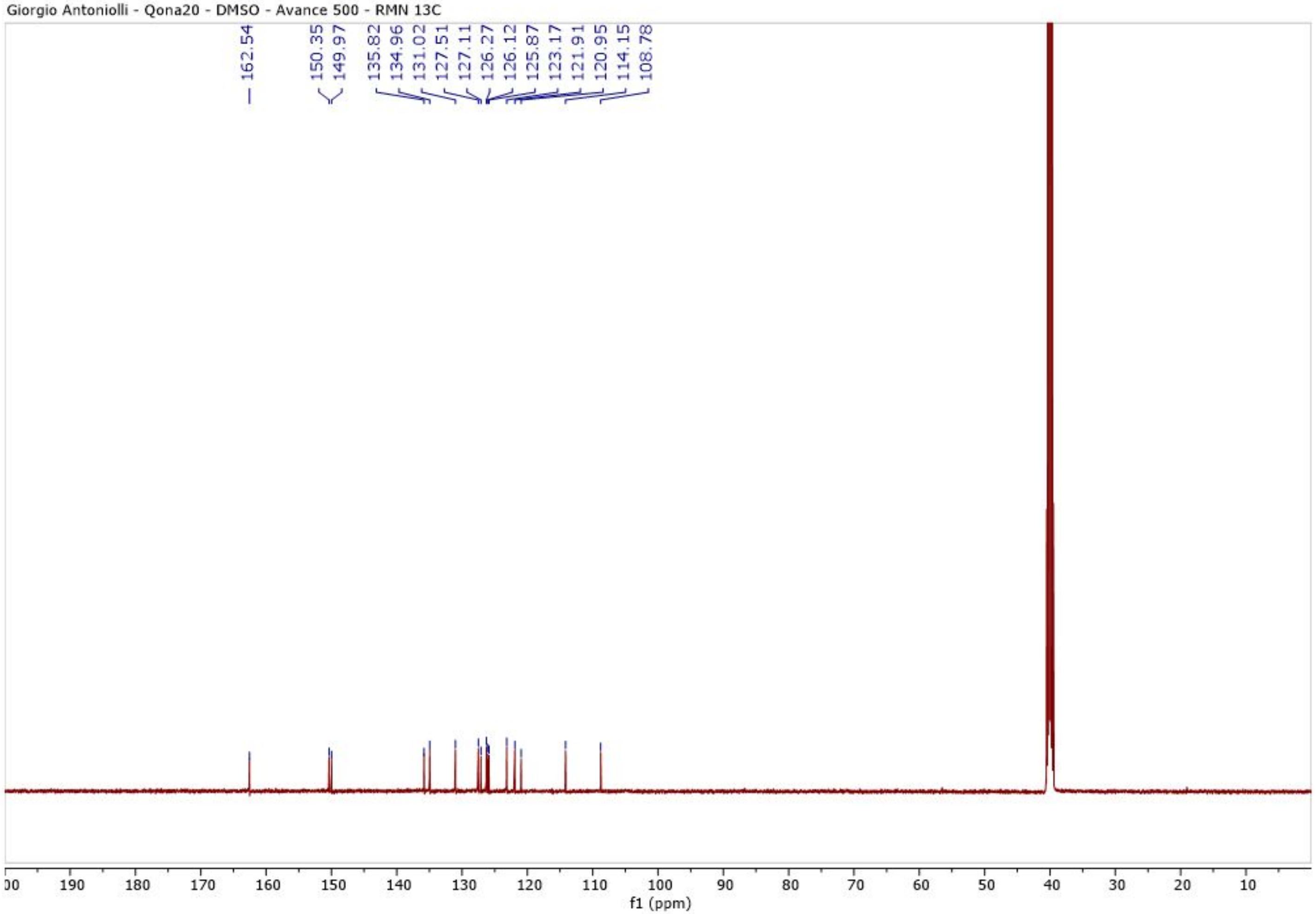

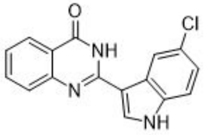

Figure 78S. ^{13}C NMR (d$_6$-DMSO, 125 MHz) of 2-(5-chloro-1*H*-indol-3-yl)quinazolin-4(3*H*)-one, **20**.

Sample ID:	Qona20	Method Name:	PADRAO ATR
Sample Scans:	128	User:	Admin
Background Scans:	128	Date/Time:	28-Jun-24 6:02:13PM
Resolution:	2 cm-1	Range:	4,000.00 - 400.00
System Status:	Good	Apodization:	Happ-Genzel
File Location:	C:\Program Files\Agilent\MicroLab PC\Results\Qona20_2024-06-28T18-04-30.a2r		

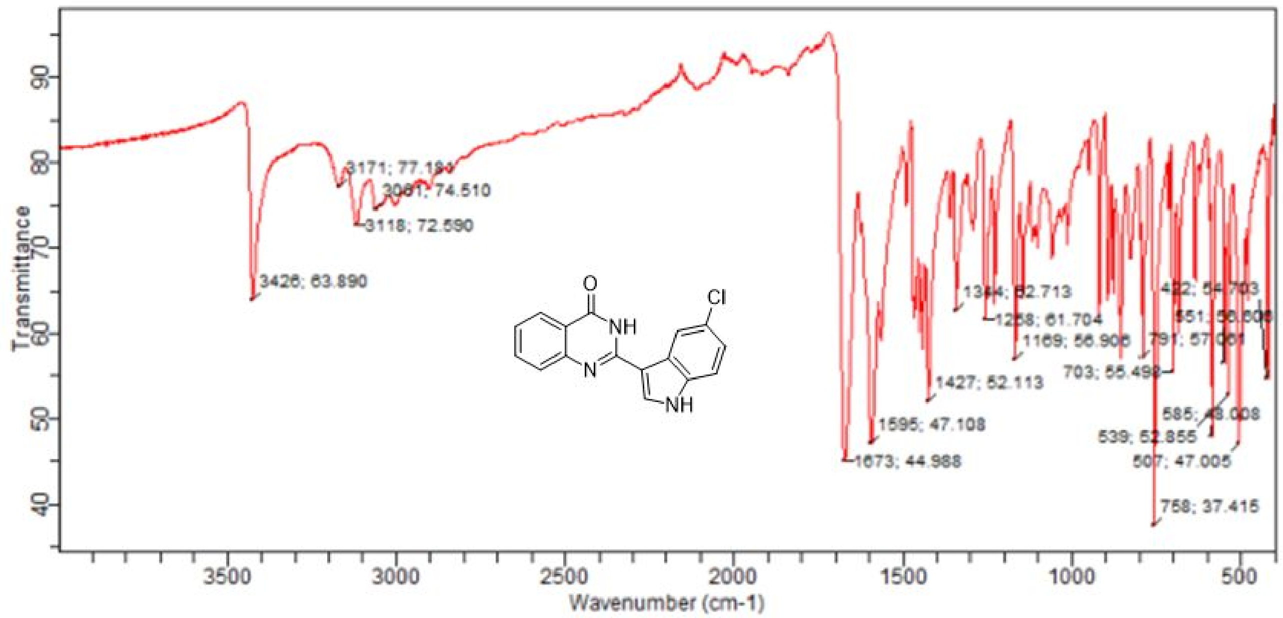

Figure 79S. IR (ATR, ν_{max}, cm^{-1}) of 2-(5-chloro-1*H*-indol-3-yl)quinazolin-4(3*H*)-one, **20**.

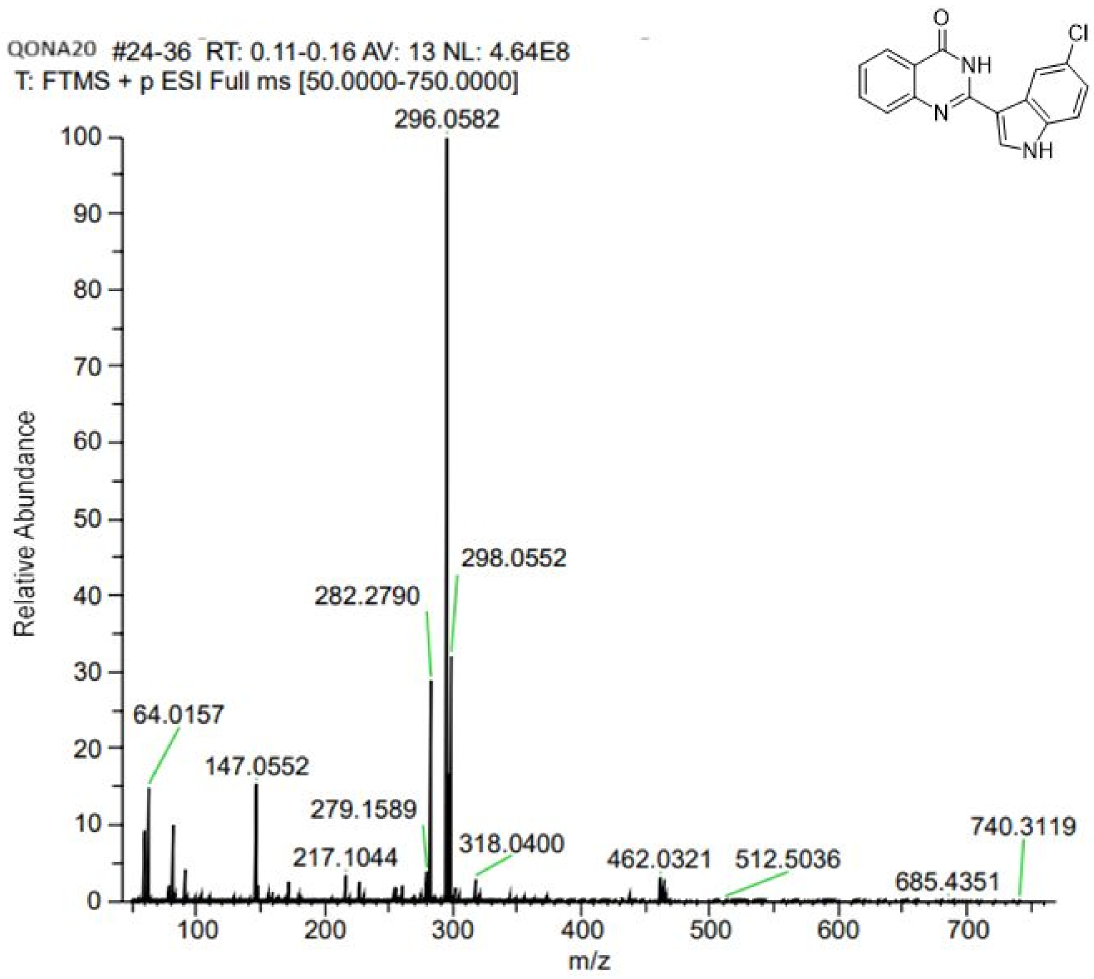

Figure 80S. MS of 2-(5-chloro-1*H*-indol-3-yl)quinazolin-4(3*H*)-one, **20**.
